# Medicinal plant use by the Tujia people in northeastern Guizhou, China: an ethnobotanical study

**DOI:** 10.3389/fphar.2025.1522456

**Published:** 2025-03-28

**Authors:** Jian Xie, Pan Wang, Qin Jiang, Qiuyi Chen, Min Xiao, Wanke He, Xuqiang Nie, Sha Liu, Yongxia Zhao, Guisen Zheng, Zhixun Bai, Shuo Li, Faming Wu

**Affiliations:** ^1^ School of Pharmacy, Zunyi Medical University, Zunyi, China; ^2^ School of Pharmacy, School of Pharmacy, Gansu Health Vocational College, Lanzhou, China; ^3^ School of Pharmacy, Gansu University of Chinese Medicine, Lanzhou, Gansu, China

**Keywords:** Tujia, eastern Guizhou, traditional herbal medicine, ethnobotany, wild plants

## Abstract

**Introduction:**

This study focuses on the traditional medical knowledge of the Tujia ethnic minority in Guizhou, particularly the use of traditional herbs with potential for development. The research documents and aims to protect this knowledge, which is vital for preserving the cultural heritage and enhancing the medicinal resources of the Tujia people.

**Methods:**

This study was conducted in the Tongren region of eastern Guizhou, the primary settlement area of the Tujia people. We used ethnobotanical and ethnoecological research methods, including field surveys, interviews with local herbal doctors, and documentation of medicinal plant species.

**Results:**

A total of 168 traditional Tujia medicinal plants from 70 families were recorded. The most represented families included Liliaceae (12 species), Crassulaceae (9 species), Asteraceae (9 species), Orchidaceae (9 species), Rosaceae (7 species), and Apiaceae (7 species). Among these, the three most frequently recorded medicinal plant species by our 124 informants were *Artemisia lavandulifolia* Salisb., *Pyracantha* (L.) Voss, and *Urtica japonica* Thunb. These plants are primarily used to treat injuries, insect and snake bites, rheumatic pain, and gastrointestinal diseases, reflecting the local climate and ecological conditions. We identified seven primary herbs that are also traditional wild edible plants crucial to the Tujia people’s daily lives. However, issues such as the aging of traditional Tujia herbal doctors, lack of successors, and unsystematic and unsafe medicinal practices were also identified.

**Conclusion:**

The findings provide essential information for preserving the traditional cultures and developing the medicinal resources of the Tujia people. There is a need for systematic documentation and training to ensure the transmission of traditional knowledge to future generations. Future research should focus on the in-depth study and development of the valuable herbs identified in this study, aiming to integrate traditional knowledge with modern scientific approaches for better healthcare solutions.

## Introduction

Traditional Chinese Medicine (TCM) is renowned globally, while traditional ethnic medicine from China, though rich in cultural heritage, remains less widely recognized. These diverse ethnic medical systems reflect unique practices and knowledge passed down through generations, each contributing to the broader landscape of ethnomedical traditions in China. Among China’s fifty-six ethnic groups, nearly every minority has developed a distinct medical culture. Preliminary surveys conducted among 35 ethnic minorities have cataloged extensive medicinal resources. These surveys have documented nearly 3,000 Tibetan medicine, approximately 1,300 Mongolian medicine, approximately 700 Zhuang medicine, approximately 600 Uyghur medicine, and close to 500 Miao medicine (Sun et al., 2020). Notably, several ethnic groups have established complete medicinal systems, including Tibetan (Luo, 2015), Mongolian (Zhang et al., 2015), Uyghur, Miao (Wang et al., 2022), and Gelao medicine (Peng and Hu, 2016).

In particular, Miao medicine features a diagnostic model encompassing two categories, five meridians, thirty-six syndromes, and seventy-two diseases. Its theoretical foundation includes theories such as ontogeny, the five basic elements, the three realms, the theory of three victories, the nine-frame structure, the essential elements of qi, blood, and water, the theory of interaction, the four major channels, the theory of the soul, and the toxic air theory, which assert that disorder causes disease. Due to its unique advantages and continuously advancing research, Miao medicine has rapidly developed in recent years and is now a mature and well-established independent discipline (Wang et al., 2022). With centuries of empirical accumulation, Tibetan medicine possesses unique therapeutic experiences and medicinal formulas, making it the second-largest traditional Chinese medicine system rich in experience for treating various diseases (Pan et al., 2021). Moreover, some smaller minority groups, despite lacking a systematic ethnomedical culture, have accumulated valuable medicinal experiences through long-term struggles with disease. The “Catalog of Zhuang Medicinal Resources,” published in 2013, lists 2,285 Zhuang medicine originating in the Western Zhou period. It is known for its distinctive ethnic and regional characteristics and is commonly used to enhance physical strength, improve immunity, and increase resistance (Zhou, 2019). The Yao people have established their medicinal knowledge base, with records for treating trauma and orthopedic, dermatological, rheumatic, and digestive system diseases. Common treatments include decoctions, pastes, moxibustion, and cupping (Lu et al., 2022). With a history of more than a thousand years, Hui medicine is a crucial part of traditional Chinese medicine. The Hui, the most widely distributed minority group in China, incorporates elements from Persian, Arab, Han, Mongolian, Uyghur, and other ethnicities, making Hui medicinal practices distinct (Zhang, 2017).

Tujia, an ancient ethnic group with a rich history exceeding a millennium, predominantly inhabits the Wuling Mountains, covering approximately 100,000 square kilometers at the eastern terminus of the Yunnan-Guizhou Plateau in China. As of the 2020 national census, the Tujia population totaled 9,587,732, ranking them as the eighth largest ethnic group in China—following the Han, Zhuang, Hui, Manchu, Uighur, Miao, and Yi—and the fifth largest in Guizhou Province (Duab et al., 2023). Tujia self-identifies as “Biz” or “Biji,” terms that may share etymological links with the self-designations of the Tibetan (“བོད་ bod”) and Pumi (“phrin/phron”) peoples, who traditionally connote “white people.” This nomenclature reflects the cultural significance of the color white among the Tujia, as well as among the Tibetan, Qiang, and Pumi ethnic groups (Tian, 2005). Historical accounts suggest that the ancestry of Tujia may be traced to the ancient Ba and Bapu peoples, likely resulting from the amalgamation of several ethnicities (Chen et al., 2016).

In China’s Wuling Mountain area, the traditional medical culture of the Tujia exhibits uniqueness and deep historical roots. Tujia predominantly resides in the provinces of Hunan, Hubei, Chongqing, and Guizhou (Tian, 2005). The Tongren area in northeastern Guizhou, a cradle of traditional Tujia culture, accounts for 17.82% of the national Tujia population and 32% of Guizhou’s Tujia population (Li and Yang, 2016).

Over the last 2 decades, traditional Tujia medicine has undergone substantial advancements. Scholars have organized and summarized traditional Tujia medicine, gradually evolving it from experiential knowledge to systematic theory. Central to Tujia medical theory is the “Three Elements Theory,” which is deeply influenced by concepts from the “I Ching” (Book of Changes). This theory incorporates the Taiji (the supreme ultimate), symbolizing the dual principles of Yin and Yang, the numerological systems of the Luo River, ancestral totems, celestial numerology, and a philosophy grounded in primitive naturalism. According to Tujia medical philosophy, human anatomy is segmented into three corresponding cosmic sections: the brain, heart, and lungs represent the heaven (upper element); the spleen and stomach align with the Earth (middle element); and the liver and kidneys correspond to the water (lower element). This framework also includes a medical theory where Yang governs Yin. Human bodily substances consist of three elements: Qi (vital energy), blood, and essence. Pathogenic factors include pathogenic Qi (or toxic Qi, encompassing external and internal toxins), emotions, and physical injuries. Pathological changes result from imbalances in Qi and blood, temperature dysregulation, and disturbances in Qi, blood, and essence. Diagnostically, Tujia practitioners employ the “Five Diagnostic Methods”: inspection, inquiry, auscultation, pulse analysis, and palpation. Clinical treatment approaches are comprehensive and include internal treatments—medications taken orally—and external or nonpharmacological traditional therapies. The eight principles of medication use include warming the cold, cooling the heat, tonifying deficiencies, purging excesses, opening blockages, reducing swelling, calming shocks, and eliminating dampness. The seven therapeutic methods include inducing sweating, purging, expelling, stopping, tonifying, warming, and clearing. Traditional external treatments, known as the “Unified Five Techniques,” integrate knife techniques, needle techniques, fire therapy, water therapy, and herbal therapy (Chen et al., 2017).

Tujia medicinal practitioners classify medicinal substances according to three thermal properties—cool (cold), warm (hot), and neutral (mild)—and nine distinct flavors: bitter, sour, astringent, numbing, spicy, salty, sweet, bland, and slippery. These classifications are integrally linked, where the flavors of the medicines correlate strongly with their thermal properties. Specifically, medicines characterized as bitter, sour, or astringent are generally considered cool or cold, while those with numbing, spicy, or salty flavors are classified as warm or hot. Sweet, bland, or slippery medicines are typically deemed neutral. This categorization aligns with the three elements theory, reflecting the tripartite nature of medicinal properties in Tujia pharmacology (Xu and Yu, 2011). In the practice of Tujia medicine, when the flavor of a medicine is fixed, it is a composite. A single medicine can have multiple flavors, which may alter its properties. Therefore, the medical theory of Tujia determines a medicine’s properties based on its primary flavor (Chen et al., 2016). The relationship between the flavors, properties, and functions of medicines in Tujia medicine is the culmination of long-term clinical practice. Medicines with bitter flavors are used to expel toxins, reduce inflammation, and alleviate food stagnation. Sour and astringent flavors are employed to consolidate body fluids, constrict body tissues, and provide relief from excessive sweating and diarrhea. Numbing and spicy flavors are utilized to dispel pathogenic influences such as wind cold and wind heat, alleviate pain, and strengthen yang energy. Salty flavors are indicated for their ability to detoxify and dissolve nodules and soften hardened masses. Sweet and bland flavors are known to nourish and soothe, addressing pain and urgency, while medicines that are both bland and slippery support the maintenance of primal essence and yang, enhance longevity, detoxify, and promote the body’s natural purification processes (Tang, 2019).

The medical culture of the Tujia ethnic group differs significantly from that of other ethnic medicines in China in terms of the species of herbs used, processing methods, and concepts of efficacy. For example, compared to the strict categorization of medicinal materials and standardized processing procedures in traditional Chinese medicine, Tujia medicine emphasizes the immediate collection and use of herbs, reflecting its direct dependence on natural resources and profound ecological wisdom (Ke et al., 2021). Additionally, the flexibility of the selection and use of medicinal materials in Tujia medicine contrasts sharply with that of other ethnic medicines, such as Tibetan and Mongolian medicines.

Tujia ethnomedicine represents the culmination of traditional medical practices among the Tujia people, characterized by a distinct medical theory that has evolved from long-standing folk practices and is uniquely Tujia. As the Tujia people have their own spoken language but no written script, there are no ancient texts of Tujia medicinal knowledge recorded in their language. However, scattered records of folk medicine can be found in historical documents written in Chinese characters from the Ming and Qing dynasties, such as “The Materia Medica of Tu Wang (Gzfwz, 2022) and “Yi Xue Cui Jing” from the Qing dynasty (woodblock edition) (Lin et al., 2018). Through a process of oral tradition and generational enrichment over centuries, this knowledge has been passed down in the unique form of oral documentation. By collecting and organizing these oral documents and folk medical manuscripts, it was discovered that these folk medicines and local therapies have been refined by generations of herbalists through practical exploration and experience, resulting in a form of Tujia medicine that is distinctive in style, broad in content, and rich in ethnic flavor.

Ethnopharmacological systems around the world exhibit distinct characteristics shaped by local ecosystems, cultural traditions, and historical contexts. For example, in India, particularly among minority tribes inhabiting forests, traditional medicine has evolved through diverse customs, with knowledge and practices transmitted across generations. These systems not only sustain the livelihoods and health of these communities but also reflect the deep ecological integration and cultural diversity of India (Sureshkumar et al., 2018). In Ethiopia, traditional medicine plays a crucial role in addressing health issues in humans and livestock, especially given the limited access to modern healthcare services. This practice is deeply entrenched in the local culture, highlighting a long-standing reliance on indigenous knowledge (Kefalew et al., 2015). Pakistan’s traditional medical system also emerges from its historical and economic backdrop, where medical knowledge is largely derived from personal experience and ancestral wisdom. This has led to the development of unique medicinal practices adapted to local needs (Ahmad et al., 2015). Similarly, in Iran, traditional medicine continues to play an essential role in healthcare, with several ancient methods still actively used to manage various health conditions (P et al., 2020). Similar to Tujia medicine, these ethnopharmacological systems rely on a profound understanding of the local ecosystem, incorporating rich cultural and spiritual elements into their practices. However, differences in ecological environments, cultural traditions, and historical contexts among regions contribute to differences in the selection of medicinal plants, treatment methods, and the connotations of medical culture among these ethnic groups. These variations not only reflect different human adaptations to nature but also enrich the diversity of global medicinal plant cultures.

As urbanization progresses and modern medical technologies rapidly develop, the medicinal culture of the Tujia ethnic group faces the risk of extinction. Our research on traditional Tujia medicine has revealed significant regional disparities. Most existing research has focused on the Enshi area of Hubei (primarily by researchers from Hubei University for Nationalities) (Yang, 2008) and the Xiangxi area of Hunan (Wang et al., 2022). In contrast, the literature on traditional Tujia medicinal practices in the Guizhou region is scarce and nearly nonexistent. Therefore, we conducted an in-depth investigation into the traditional herbal medicine of the Tujia people in Guizhou, aiming to uncover, document, and organize the cultural knowledge of traditional medicine in this region. This work not only supplements and enriches the traditional medicinal culture system of the Tujia people but also holds significant importance for preserving the traditional medicinal cultural knowledge of northern Guizhou.

Through an in-depth study of the traditional use of herbal medicines by the Tujia people in the Tongren area, we identified 168 different herbs from various plant families, demonstrating the rich knowledge and utilization of natural resources by the Tujia people. These herbs are used not only to treat a wide range of diseases but also to reflect the Tujia people’s philosophy of living in harmonious coexistence with nature. This article will explore the species, uses, historical background, and cultural significance of these herbs, providing a comprehensive perspective for understanding and preserving this unique ethnic medicinal culture.

## Materials and methods

### Study area

**FIGURE 1:**
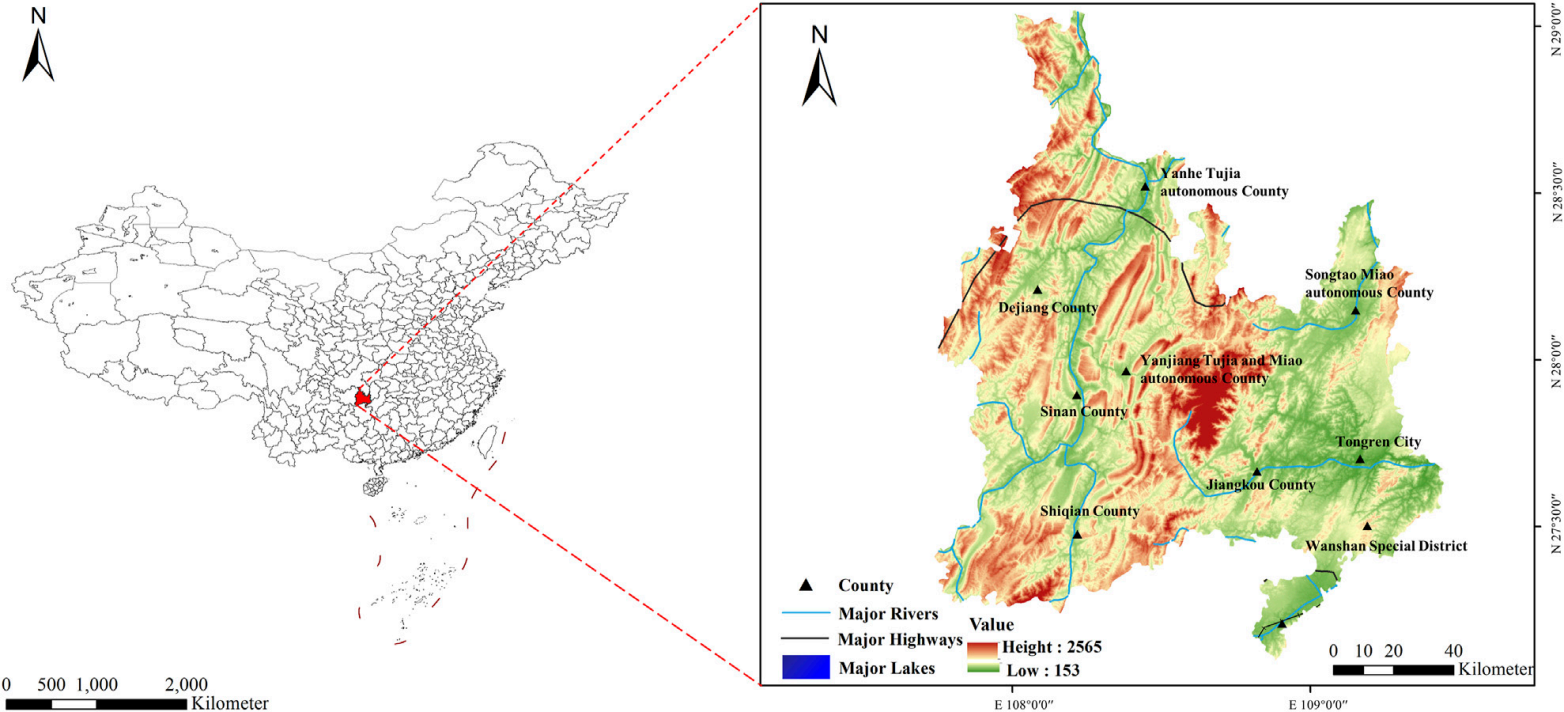
Survey area.

Our research focused on the systematic investigation of traditional knowledge used by the Tujia people in Guizhou for disease prevention, treatment, and health maintenance using wild plant resources. This study covers the areas of Yanhe, Yinjiang, Sinan, Dejiang, and Shiqian in the eastern part of Tongren, Guizhou. Tongren is located in eastern Guizhou Province, China, spanning from 27°18′to 28°26′N and 108°14′E to 109°53′E, with elevations ranging from 2,572 to 205 m (Figure 1) (Yang, 2017). The region experiences a humid subtropical monsoon climate, with an average annual temperature of 16.2°C (Wen et al., 2014), characterized by hot, humid summers and cold, dry winters (Supplementary Table 1).

The counties of Yanhe, Yinjiang, Sinan, Dejiang, and Shiqian in Tongren boast beautiful environments and rich ecological resources. The landscape features large tracts of primary forests and grand canyons, providing a diverse and unique ecosystem. In terms of fauna and flora, in addition to rare species such as the giant salamander and the Guizhou golden monkey, a variety of medicinal and wild edible plants grow here, providing abundant natural resources for the local Tujia people to treat diseases and maintain daily healthcare (Yang et al., 2014).

Culturally, the Tongren area has a long history and a rich diversity of ethnic cultures (Dai and Jiang, 2012). It is a multiethnic settlement where the Tujia, Miao, and Han are the predominant ethnic groups. Tujia is one of the major ethnic groups in the region, with four Tujia autonomous counties and 30 Tujia autonomous townships, comprising nearly 30% of the local population (Ni et al., 2025). Tujia integrate themselves with the mountains, creating unique ethnic settlements, architecture, and farming cultures. They continuously absorb advanced cultures from various ethnic groups, forming a rich Tujia ethnic flavor and creating a variety of folk arts. These include the elegant Tujia hand-waving dance, the humorous Tujia lantern festival, the passionate Tujia mountain songs, the rustic Tujia boatman’s call, the deeply moving “crying marriage song,” the “grass-pulling drum,” closely linked with singing mountain songs and drumming, the Tujia Nuo opera known as the “living fossil of Chinese drama,” aesthetically pleasing and functional Tujia stilt houses, a unique culinary culture, exquisite handicrafts, and legendary marriage customs and festival activities (He et al., 2022).

Economically, the region is rich in mineral resources, such as mercury and manganese, which are the most distinctive local industries (Que et al., 2025). Tourism is also a key pillar of the local economy. The innovation and enthusiasm of ethnic peoples attract numerous tourists for sightseeing, making them a resource with great economic and cultural potential, such as Fanjing Mountain in Yinjiang (Yang et al., 2024).

### Fieldwork methodology and data collection

During the fieldwork phase of our study on traditional Tujia herbal medicine, we employed a diverse set of ethnographic research methods to ensure a comprehensive collection of in-depth information. The primary techniques included key informant interviews, semistructured interviews, and participatory observation (Silveira et al., 2012), all of which were structured around the “5 W+1H” interview framework (Who, What, When, Where Why, and How) (Umay et al., 2022). This framework facilitated a detailed exploration of the inheritance and application of herbal knowledge among the Tujia people.

**FIGURE 2:**
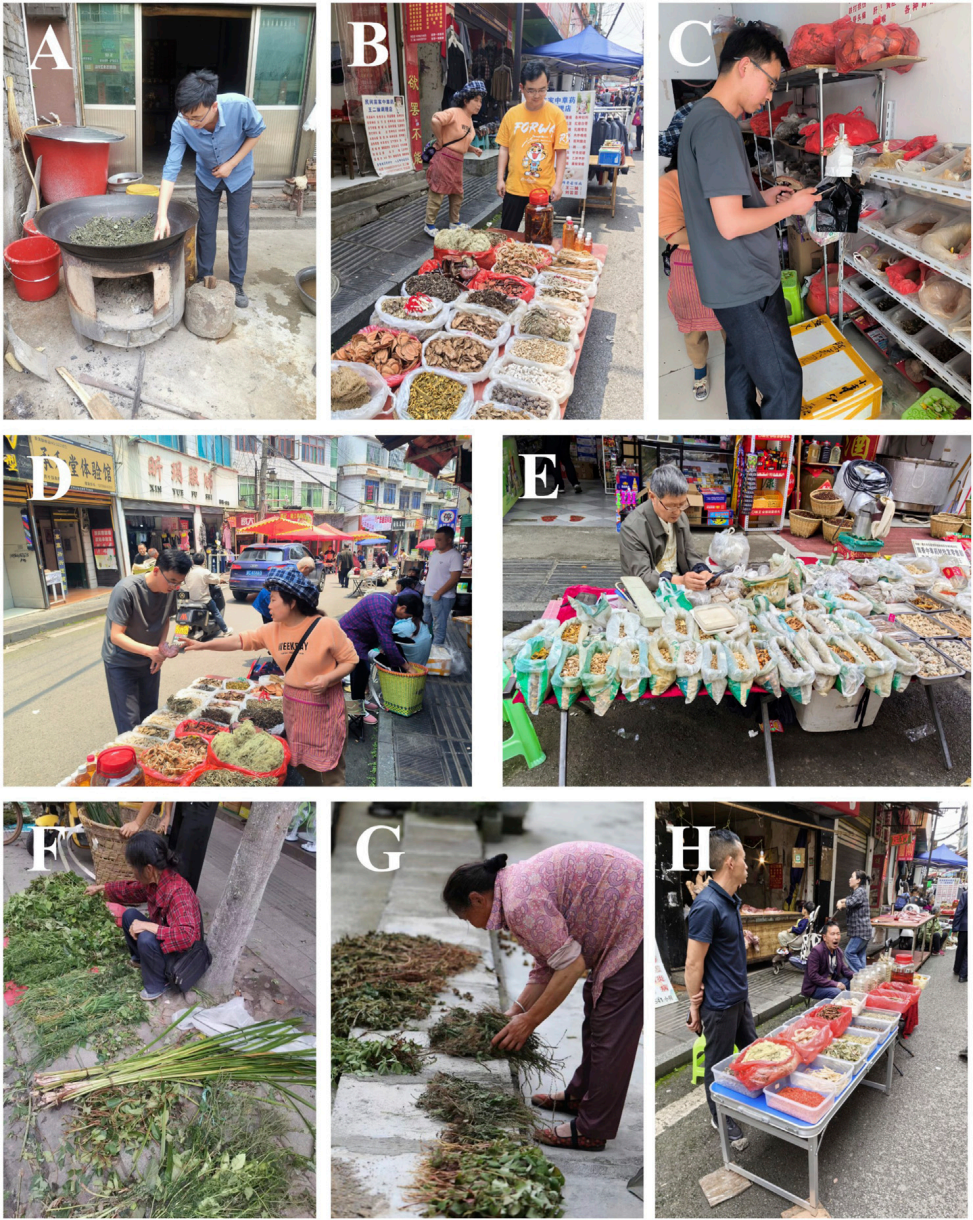
Ethnobotanical Investigation of the Tujia Ethnic Group. **(A–D)** Authors conducting field research at local herbal stalls. **(E–H)** Local herbal doctors selling medicinal herbs on the market.

We assembled a team of eight Tujia traditional herbal medicine information collectors, all of whom were descendants of Tujia and undergraduate students at Zunyi Medical University studying Pharmacy and Chinese Medicine. These collectors had a profound understanding of the cultural background of the Tujia people and possessed the necessary professional knowledge to support the study. Throughout the research process, we strictly adhered to ethical guidelines, including compliance with the Nagoya Protocol and securing field authorization from local authorities. Before data collection, we obtained prior informed consent from all participants, who were informed of the study’s objectives and the intended use of the collected information. They were assured that the research was purely for academic purposes. Through in-depth interviews and surveys, the collectors interacted with locals with rich traditional knowledge, including herbal doctors, herb gatherers, and herbal merchants, gathering insights into Tujia traditional herbal medicine (Figure 2). The collected information, such as local names, medicinal parts, processing methods, efficacy, and safety, was meticulously documented. To ensure that the community benefits from the research, we plan to share the study’s findings with the participants and local organizations, contributing to the preservation and application of their traditional knowledge.

To enhance their practical skills and knowledge, the collectors apprenticed with local herbal doctors and farmers, learning firsthand how to identify and authenticate medicinal plants. Guided by local guides, the collectors personally gathered local herbs; meticulously recorded details about the herbs’ growing environments, collection locations, and times; and prepared the herbal specimens. After comparison with the literature and expert verification, these specimens and related data confirmed the botanical classification and medicinal value of the herbs, including their specific usage and dosage, providing a scientifically accurate reference for the study.

By employing a comprehensive research approach, we aim to understand and document the traditional knowledge of the Tujia people in the Tongren region of Guizhou regarding the use of wild plant resources for disease prevention, treatment, and health maintenance. This study seeks to contribute to the preservation and transmission of this valuable intangible cultural heritage.

### Quantitative evaluation system for ethnobotany

Our study developed a comprehensive quantitative evaluation system that integrates methodologies from ethnobotany (Magurran, 1998; Wang, 2010) and ethnoecology (Troussellier and Legendre, 1981). This system is designed to assess various dimensions of medicinal plant use within different villages. It includes the following indices:

The **Simpson index (*D*)** was used to assess the evenness of medicinal information obtained from different villages: ***D =* ∑*Pi*** ^***2***^, where *D* is the evenness index, *S* is the number of medicinal species, and *P*_*i*_ is the proportion of informants reporting on medicine i out of the total number of medicinal reports.

**The Shannon‒Wiener index (*H′*)** measures the richness of medicinal information obtained from different villages: ***H′* = -∑*Pi ?lnPi*,** where *P*_*i*_ is the probability of the first informant in a village mentioning medicine i, *P*_*i*_ = *N*_*i*_/*N*, *N*_*i*_ is the number of informants for medicine i in the village, and *N* is the total number of informants for all medicines in that village (Feng et al., 2021).

**The Utilization Frequency (HUF)** evaluates local adaptation strategies to the environment and the degree of utilization of medicinal resources: ***f = N***_***m***_ ***/N***_***i***_, where *f* is the utilization frequency, ***N***_***m***_ is the number of informants who mentioned the medicine, and ***N***_***i***_ is the total number of informants.

The **National Cultural Significance Index (NFSI)** assesses the importance of each medicine in the lives of local residents: ***NFSI = FQI × AI × FUI × PUI × MFI × CEI × DSI × 10*** ^***–2***^, where *FQI* is the frequency quotient index (number of informants mentioning a medicine), *AI* is the availability index, *FUI* is the frequency of use index, *PUI* is the part used index, *MFI* is the multifunctional use index, *CEI* is the efficacy index, and *DSI* is the safety index (Xie et al., 2023b).

### Specimen identification

Throughout the survey, species identification was rigorously conducted by Professor Faming Wu using authoritative resources, including the full electronic version of the “Flora of China” (Iplant, 2024), the “Hengduan Mountains Flowering Plants Atlas” (Niu and Sun, 2021), and the “Field Guide to Common Plants of China - Hengshan Volume” (He, 2017). Identification involved observing the morphological characteristics of plants such as leaves, flowers, and fruits and consulting the botanical literature for comparison. This process resulted in the classification of species and specimen preparation, with the collected data organized and analyzed according to research objectives and illustrations drawn. Each specimen was assigned a unique voucher number, with detailed records of the collection location, time, environmental conditions, and collector information. These specimens were preserved in the specimen room of the School of Pharmacy at Zunyi Medical University.

## Results

### Demographic analysis of informants

Our field survey encompassed 11 Tujia villages and included 124 effective informants (those who could provide information on the use of plant medicine by the Tujia people, primarily Tujia herbal doctors and herbal merchants). Among these participants, 57 had experience utilizing traditional Tujia herbal medicine to treat diseases, with some still operating herbal shops. The information provided by these informants was generally more detailed and enriched compared to other participants, particularly in terms of specific plant uses, preparation methods, and therapeutic effects.

**FIGURE 3:**
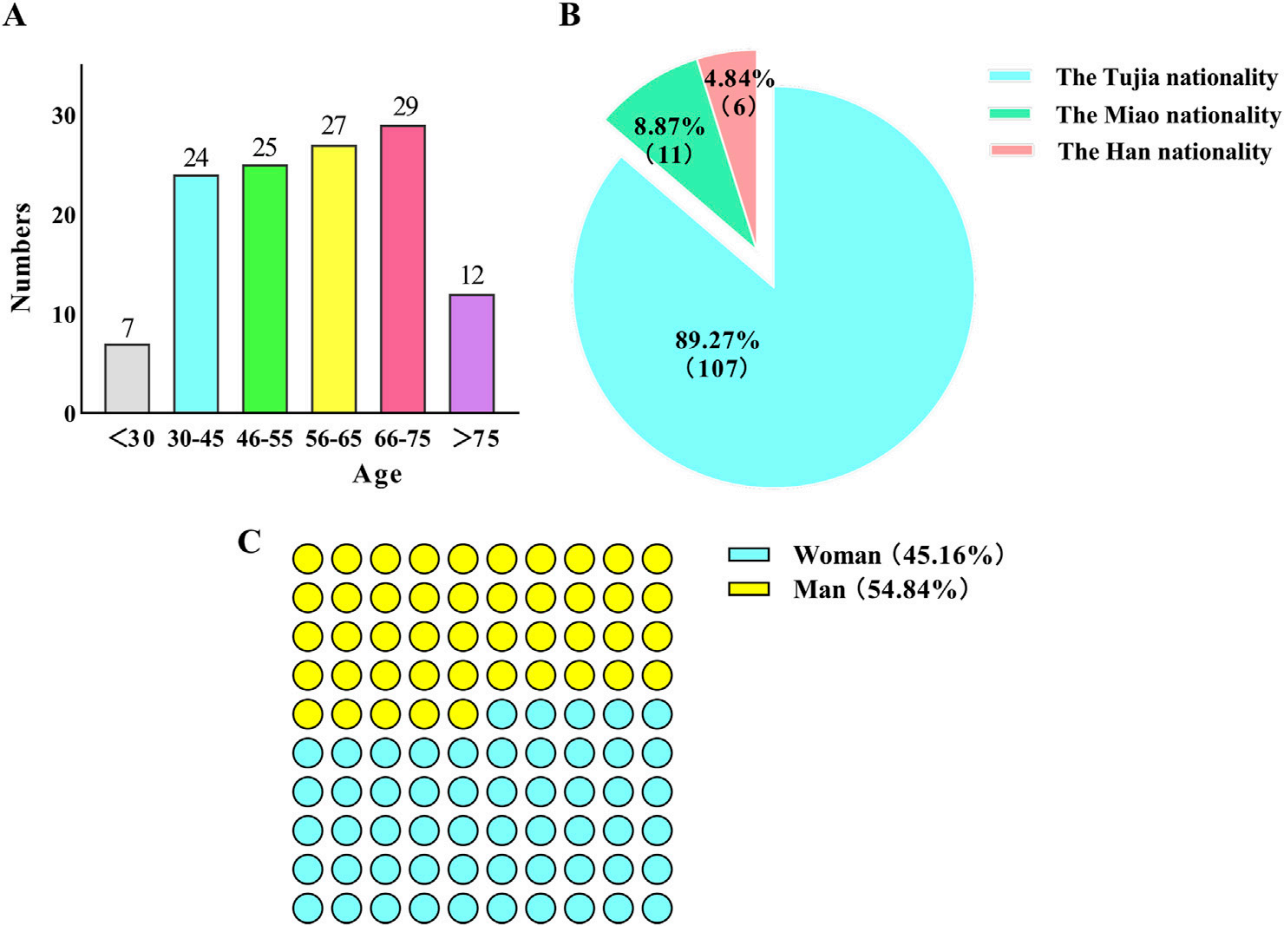
Demographic profile of the informants. **(A)** Age structure of the informants. **(B)** Gender ratio of informants. **(C)** Ethnicity ratio of the informants.

A detailed statistical breakdown of the informant demographics revealed a broad age range from 25 to 89 years. The age distribution included 7 individuals under 30 years, 24 between 30 and 45 years, 25 between 46 and 55 years, 27 between 56 and 65 years, 29 between 66 and 75 years, and 12 over 75 years. This distribution suggested that while the age range of the informants was wide, the majority were middle-aged or older. The gender representation consisted of 68 males and 56 females, resulting in a male-to-female ratio of 1.2:1. An ethnic breakdown revealed that 107 informants were Tujia (86.3% of the total), 11 were Miao, and 6 were Han, indicating that Tujia individuals were the primary contributors to this study (Figure 3).

Our findings indicate that older individuals possessed richer knowledge of traditional medicines, while those under age 30 often provided less detailed information. Most informants who engaged in traditional Tujia medical practices were over 55 years old and typically had a low level of formal education, generally possessing only basic literacy skills. Furthermore, males provided more information on traditional Tujia medicine than females did, which might be related to the traditional custom of passing medicinal knowledge to males rather than females, although this viewpoint has largely disappeared today. Traditional Tujia herbal doctors, often elderly individuals with invaluable knowledge of local medicinal plants and healing practices, are increasingly facing challenges in passing on their expertise. The aging of these practitioners is compounded by the societal shifts brought about by modernization and urbanization. Many younger Tujia people are migrating to urban areas for better economic opportunities, leading to a generational gap in the transmission of this traditional knowledge. Moreover, the growing preference for modern medicine and the lack of structured training opportunities for young people have resulted in diminishing interest in traditional herbal practices.

### Source and diversity of traditional Tujia herbal medicine

**FIGURE 4:**
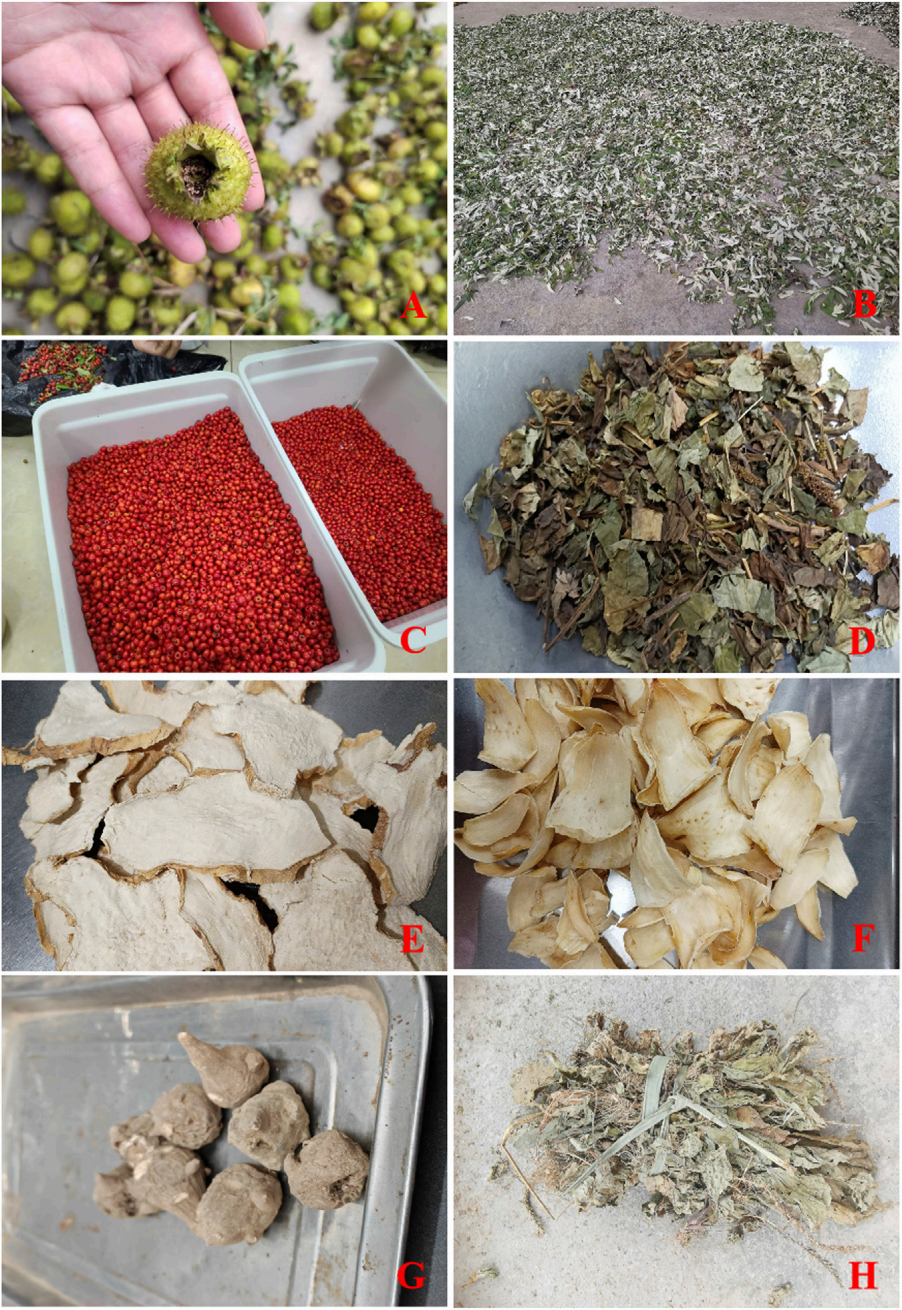
Representative Herbal Medicines of the Tujia Ethnic Group. **(A)** Ros*a roxburghii* Tratt. **(B)** *Artemisia argyi* H. Lév. and Vaniot **(C)** *Pyracantha* (L.) Voss. **(D)** *Houttuynia cordata* Thunb. **(E)** *Polygonatum sibiricum* Redouté. **(F)** *Lilium brownii* var. *Viridulum* Baker. **(G)** *Aconitum sinomontanum* Nakai. **(H)** *Plantago asiatica* L.

**FIGURE 5:**
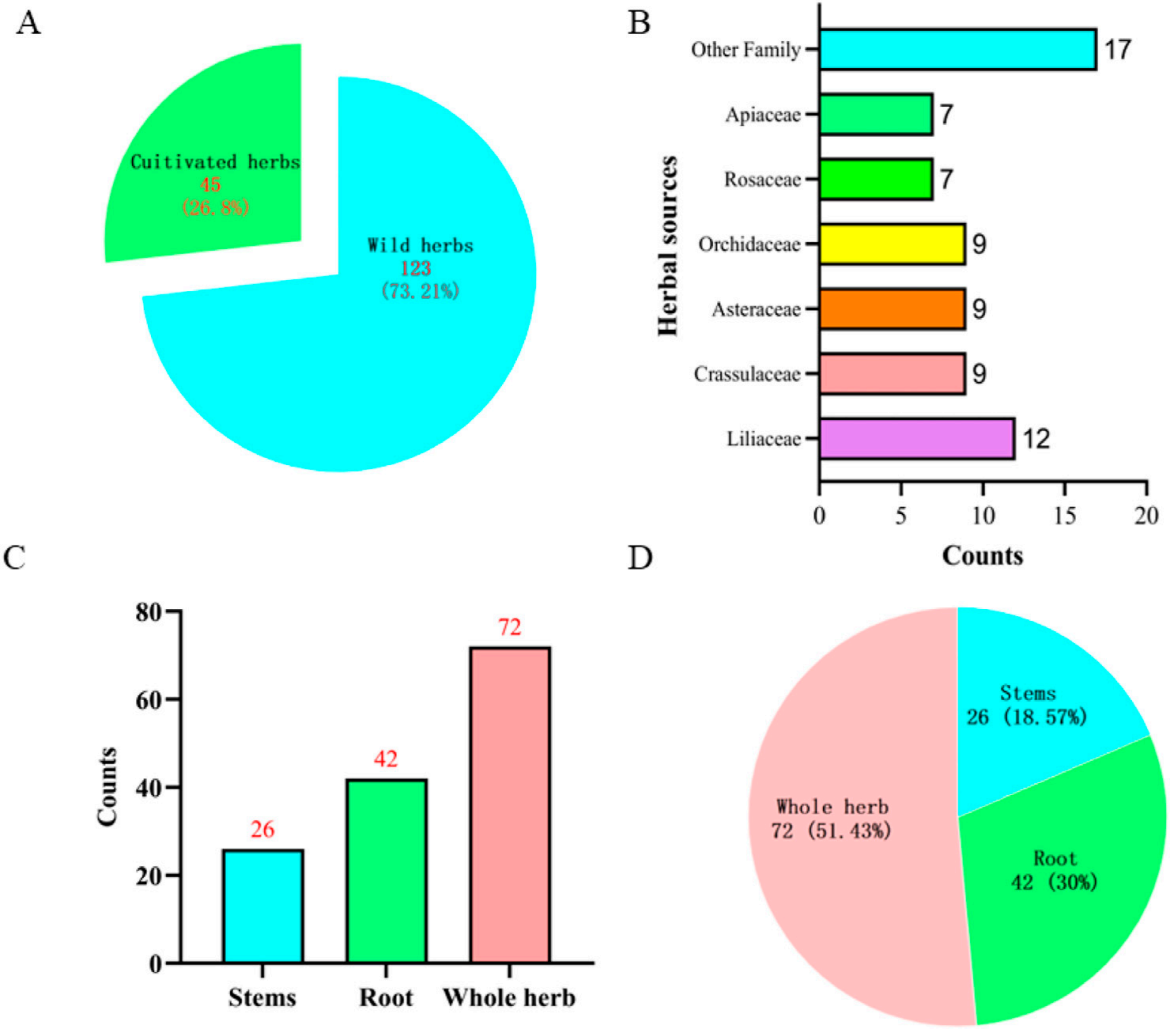
Information on traditional herbal medicine of the Tujia ethnic.

Our survey in the Tongren region identified 168 different species of herbs traditionally used by the Tujia people, primarily harvested directly from the natural environment (Supplementary Table 2) (Figure 4). Of these, 123 are wild herbs, highlighting the region’s biodiversity, while 45 are cultivated and are typically found in front of or behind residents’ homes (Figure 5A). Both cultivated and wild forms of these plants coexist locally, indicating a deep understanding of and self-sufficiency in medicinal resources by the Tujia people.

The specimens collected were found primarily at elevations ranging from 650 to 2000 m. These specimens were sourced from diverse environments, including local residents’ home gardens, roadside edges, nearby mountains, valleys, and farmlands.

Our analysis revealed that these medicinal herbs belong to 70 different plant families. The most represented families were Liliaceae, Crassulaceae, Asteraceae, Orchidaceae, Rosaceae, and Apiaceae, with 12, 9, 9, 7, and 7 varieties, respectively. The majority of these plants are herbaceous, with a total of 114, followed by vines, shrubs, trees, ferns, mosses, algae, and aquatic plants (Figure 5B).

In terms of the medicinal parts used, the whole plant is most commonly utilized, with 72, followed by roots and rhizomes at 42 and 26, respectively. Often, multiple parts of a plant are used either separately or in combination (Figures 5C, D).

The preparation methods for these herbs are generally straightforward and typically involve harvesting, washing, drying, and basic cutting. Fresh herbs are typically mashed and used on the spot. Compared to the complexity of traditional Chinese medicine preparation processes, the treatment of Tujia herbal medicine is more straightforward.

In Tujia traditional medicine, the use and identification of herbs are often more diverse than those prescribed in formal pharmacopoeias. For example, the herb Ai Cao (*Artemisiae* argyi folium) is not limited to the species *Artemisia argyi* H. Lév. and Vaniot, which is specified in the Chinese Pharmacopoeia. Other closely related species, such as *Artemisia lavandulifolia* DC. and *Artemisia indica* Willd., are also commonly used as Ai Cao. Once the leaves are processed into moxa floss, distinguishing between these species becomes nearly impossible.

Indeed, the significant differences in the information collected from each village, as revealed by our study, are likely related to the availability of herbal resources and the transmission of medicinal knowledge within local communities. To gain a deeper understanding of these differences, we analyzed the uniformity, richness, and similarity of medicinal information collected from 11 villages.

**FIGURE 6:**
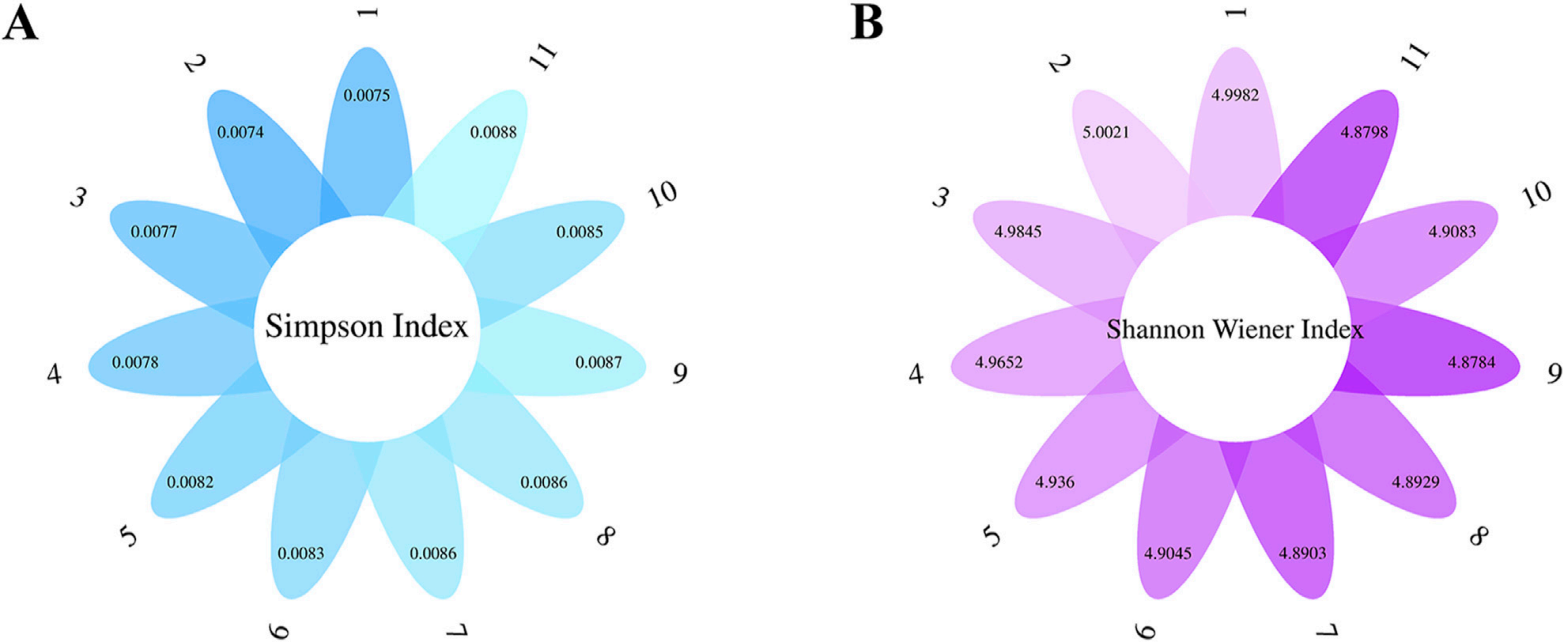
Uniformity, richness, and similarity of medical information in the survey area. **(A)** The Simpson index measures diversity and evenness. **(B)** The Shannon‒Wiener index was used to assess ecological diversity.

The Simpson index values for the medicinal information from these villages ranged from 0.0074 to 0.0088 (Figure 6A). The Simpson index measures the diversity and evenness of categories within a sample, with values closer to 0 indicating greater uniformity. This suggests that while there is a relative abundance of herbs in these villages, the distribution of different herbs is uneven.

Additionally, the Shannon‒Wiener indices were between 4.8784 and 5.0021 (Figure 6B). This index is typically used to assess ecological diversity, where higher values indicate greater information entropy and thus greater diversity and richness of medicinal species. Overall, the information gathered from the 11 villages shows low concentration and relatively high dispersion, which may be related to the diversity of the herbs obtained.

Specifically, Banxi town exhibited the lowest Simpson index and the highest Shannon‒Wiener index, indicating that the information gathered was the most dispersed and evenly distributed among the herbs. Conversely, Hongqi Street had the lowest Shannon‒Wiener index and the second-highest Simpson index, suggesting lower dispersion and a greater concentration of information. Despite the richness of medicinal species, certain herbs may be reported more frequently, indicating potential differences in knowledge sharing among informants.

These findings highlight significant regional disparities in the dissemination and application of medicinal plant knowledge among the Tujia villages. Understanding these differences is crucial for the further protection and utilization of local herbal resources, ensuring that such practices are maintained and adapted sustainably within community contexts and biodiversity conservation efforts.

### Extensive utilization of traditional Tujia herbal medicines

**FIGURE 7:**
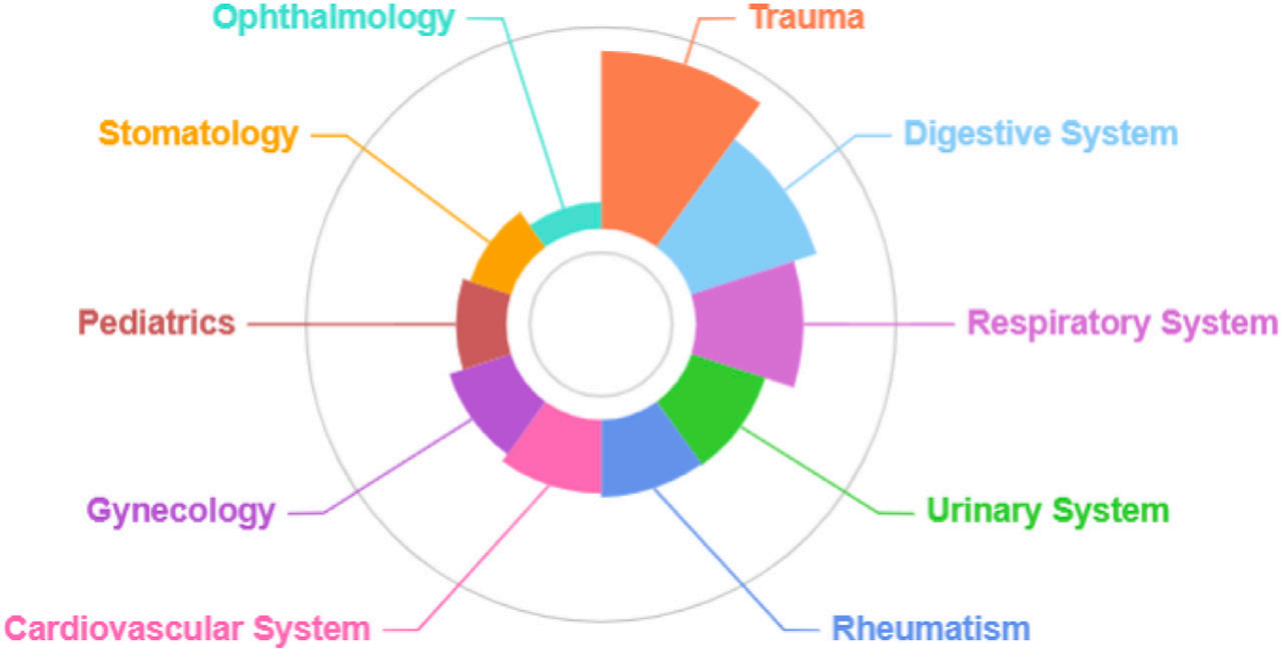
Diseases treated with Tujia medicinal plants.

Our investigation of 168 traditional Tujia herbal medicines in northern Guizhou revealed their primary use in treating more than a dozen disease categories, reflecting the diverse medicinal needs and environmental challenges of the region. Significantly, 134 of these herbs, accounting for 80% of the total, are used for treating external injuries such as bruises, bleeding from falls, and bites from insects and snakes. This indicates a strong local focus on trauma-related treatments, reflecting the environmental challenges of the region, such as dense forests, abundant rainfall, and difficult terrains where insect and snake bites are common. In addition to trauma care, there are 75 herbs used for digestive system ailments and 50 for respiratory system disorders, showcasing the traditional disease spectrum’s emphasis on these categories. Other significant areas included treatments for urinary system diseases (26 species), rheumatic conditions (25 species), cardiovascular issues (23 species), gynecological ailments (18 species), pediatric conditions (11 species), oral diseases (8 species), and ophthalmological problems (3 species) (Figure 7).

Living in rugged, mountainous areas with challenging access has influenced the medicinal practices of Tujia. Locals often use fresh medicinal materials, especially for the immediate treatment of injuries or bites. Freshly gathered herbs are commonly mashed or chewed and applied topically as a first aid measure. Internal medications are typically prepared by decoction or infusion of alcohol, with some being mashed with rice water, vinegar, or alcohol to extract juice. For external applications, fresh plants are crushed into a paste, sometimes mixed with alcohol or rice water, and applied to the affected areas or acupoints. Potentially toxic medicines are ingested by plants wrapped in rice, fruit peels, or eggs to mitigate toxicity. Skin diseases often involve washes made from herbal decoctions, while rheumatic pains and injuries frequently involve the use of alcohol-soaked or decocted herbs mixed with alcohol for both internal and external use. Fresh herb application is also common for treating these conditions.

Tujia traditional doctors display a certain flexibility in their use of medicinal quantities, often using imprecise measures such as a handful, a spoonful, a piece, or a plant. This reliance on experience rather than precise measurement reflects the informal nature of traditional medicine practices in the area, where sanitary conditions in herbal medicine facilities are also typically substandard.

Interestingly, the local population has a rich understanding of the dual use of wild plants for both food and medicine. Nonmedical informants (ordinary residents) are more aware of such dual-use plants than purely medicinal residents are. They know how to use the tender leaves and shoots of medicinal plants such as *Pteridium aquilinum* var. *Latiusculum* (Desv.) Underw. Ex A. Heller, Aster indicus L.*, Urtica fissa* E. Pritz.*, Bupleurum scorzonerifolium* Willd. And fruits such as *Akebia trifoliata* (Thunb.) Koidz.*, Rosa roxburghii* Tratt.*, Rosa rubus* H. Lév. and Vaniot as snacks. They also make soups from various plant roots, such as Codonopsis and *Dioscorea opposita* Thunb., showcasing a comprehensive utilization of natural resources.

### Evaluation of the importance of traditional herbal medicine of the Tujia ethnic

**FIGURE 8:**
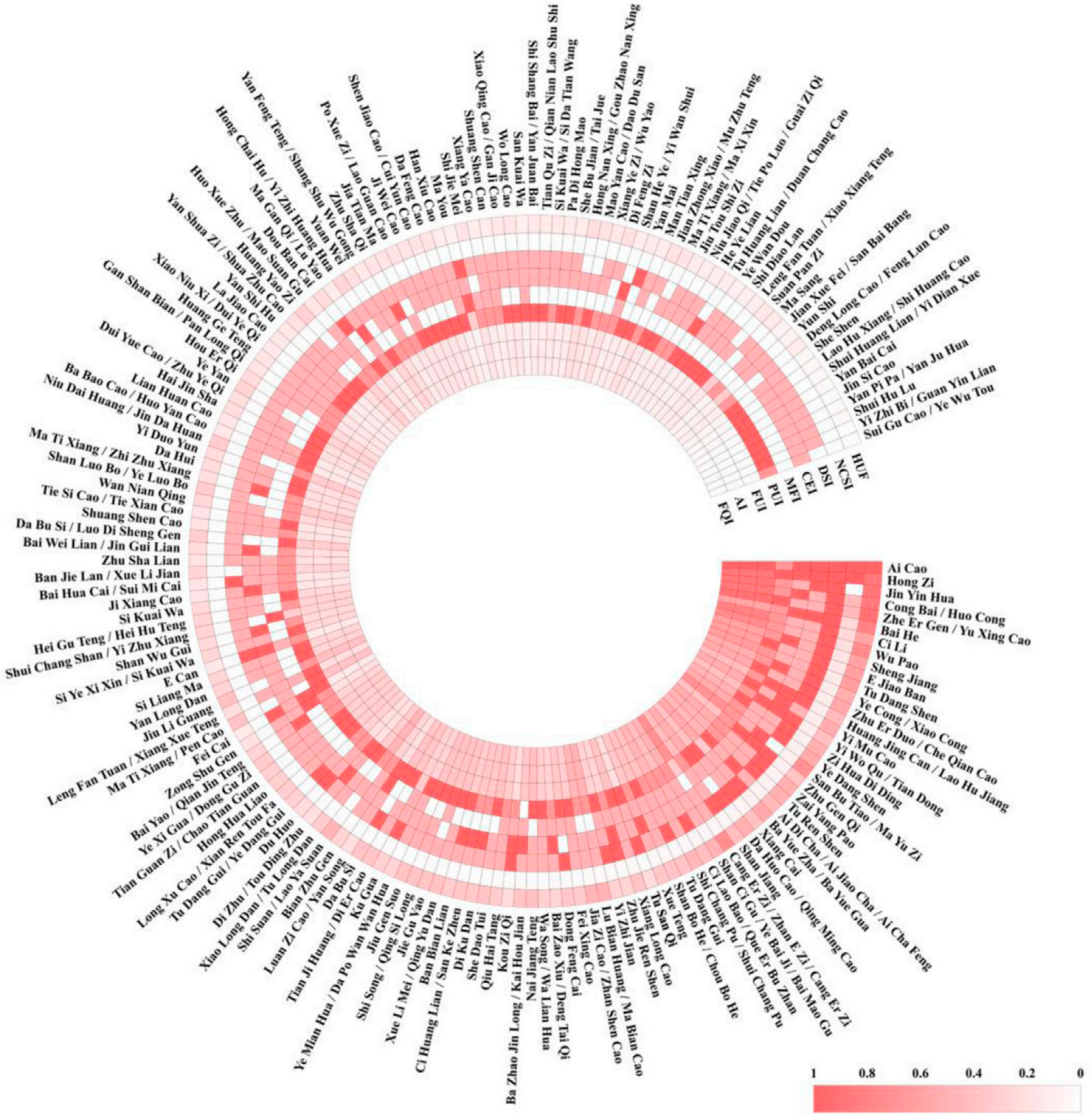
Importance index evaluation of the tujia ethnic.

We conducted a quantitative analysis of the importance of 168 species of medicinal plants traditionally used by the indigenous Tujia people in the Tongren region. The comparison results of the National Cultural Significance Index (NFSI) for the traditional use of medicinal plants by the Tujia people are shown in Figure 8. Based on the NFSI, we clustered the medicinal plants used by the Tujia people to identify the widely used, high-value herbs that play an important role in traditional healthcare among the local population.

The first important sequence (NFSI≥500) included seven traditional medicinal plants: *A. lavandulifolia* Salisb.*, Pyracantha* (L.) *Voss, Urtica japonica* Thunb.*, Allium fistulosum* L.*, Houttuynia cordata* Thunb.*, R. roxburghii* Tratt and *Lilium brownii* var. *Viridulum* Baker. This category of plants is both medicinal and edible, is widely distributed and easily accessible in the region, and continues to play vital roles in the daily lives of the Tujia people.

The second most important sequence (500 > NFSI ≥100) included 20 traditional medicinal plants, with representative species such as *Zingiber officinale* f. *Rubens* (Makino) M. *Hiroe, Campanumoea javanica Blume, Polygonatum sibiricum Redouté, and Pinellia ternata* (Thunb.) Makino, except for *P. ternata* (Thunb.) Makino. The other 19 species are also edible and relatively easy to obtain. However, the overall distribution and consumption frequency of this sequence of herbs were lower than those of the first sequence, resulting in a relatively lower importance index.

The third most important sequence (100 > NFSI ≥10) included 63 traditional medicinal plants. This sequence has a wide variety of plants, with representative species such as *Xanthium strumarium* L.*, Cremastra appendiculata* (D. Don) *Makino, Ardisia crenata* Sims/*Ardisia crispa* (Thunb.) A. DC. Although these plants are generally effective, they are mostly nonedible and relatively scarce.

The fourth most important sequence (10 > NFSI) included 77 traditional medicinal plants. Information on these plants is relatively sparse, and some plants, such as *Aconitum sinomontanum Nakai, Glochidion puberum* (L.) Hutch.*, Dioscorea bulbifera* L., have unclear reports of toxicity.

The Utilization Frequency (HUF) for these plants varied from 0.07 to 0.80, indicating that even the most commonly used herbs were not mentioned by all informants. The top ten plants in terms of utilization frequency were *A. lavandulifolia* Salisb.*, H. cordata* Thunb.*, U. japonica* Thunb.*, P. pyracantha* (L.) *Voss, A. fistulosum* L.*, R. roxburghii* Tratt.*, Rubus setchuenensis* Bureau & Franch.*, X. strumarium* L.*, P. ternata* (Thunb.) *Makino, C. javanica* Blume, which align with their NFSI scores. This consistency indicates the reliability of the evaluation model.

## Discussion

The evolution of ethnic minority medicine, such as that practiced by the Tujia people, is intrinsically linked to a confluence of historical, environmental, cultural, and socioeconomic factors (Zhao and Shi, 2019; Zhu, 2006). Predominantly residing in the Wuling Mountains—a region straddling the borders of Guizhou, Hubei, Hunan, and Chongqing—the Tujia community has cultivated a rich tradition of medicinal practices that mirrors the distinct ecological and cultural attributes of southern China. The historical backdrop of the Tujia, coupled with their unique living environment in a mountainous terrain, has shaped their traditional medical practices. These practices are characterized by the utilization of local flora and the application of indigenous knowledge systems that have been passed through generations. Cultural education levels within the community also play a pivotal role in the transmission and evolution of medicinal knowledge, influencing how traditional practices are maintained and adapted over time. Moreover, the degree of socioeconomic development in these regions affects the accessibility to and reliance on traditional medicine. In areas where modern medical facilities are less accessible, traditional medicine has not only continued to thrive but also evolved into a primary healthcare resource. The role of researchers in documenting and promoting this rich medicinal heritage is crucial. Through systematic study and dissemination of knowledge, researchers help in not only preserving these traditions but also integrating them with modern medical practices. This enhances the understanding and appreciation of Tujia traditional medicine both within and outside the community, promoting its sustainability and relevance in contemporary healthcare contexts.

### Evolution and integration of traditional medicine to Tujia

The medical culture of the Tujia people is not static but has evolved through interactions with TCM. Our review of the literature related to the application of Tujia traditional herbal medicine revealed that Tujia medicine has developed into a unique medical system by combining elements of TCM with local shamanistic practices (Liang et al., 2016). Historically, the Tujia people did not rely on formal medical practices. Instead, they addressed illnesses by performing rituals involving the slaughter of livestock and shamanistic ceremonies to expel evil spirits. Religious priests often served as community healers, and through prolonged battles with diseases, the early form of Tujia medicine began to take shape. With the spread of Han culture, TCM was introduced to the Tujia region, and cultural exchanges facilitated the rapid development of Tujia medicine, eventually forming a relatively complete medical system (Liang et al., 2016).

In terms of drug classification, Tujia healers categorize their medicines based on their natural properties and clinical attributes into various broad categories, often using the numerals 36 and 72. This classification method possibly originates from the traditional Chinese cultural concepts of the 36 Heavenly Spirits and the 72 Earthly Fiends. This system reflects the integration of Tujia medicine with traditional Chinese medicine. For example, they refer to herbs for dispelling heat and toxins, promoting blood circulation, reducing swelling, and relieving pain as “qi,” totaling 72. There are 72 medicines for unblocking meridians, reducing swelling and bruising, stopping bleeding, expelling heat and toxins, and dispelling wind and dampness called “huanyang”. Herbs for replenishing qi, moistening the lungs, strengthening the liver and spleen, and tonifying the heart and kidneys are called “shen,” similar to traditional TCM, where “shen” herbs have nourishing effects, totaling 72. Additionally, there were 72“lian,” 36“xue,” and 36“centipede” among the other classifications (Yuan, 2007).

However, during our field research in Tongren, the birthplace of the Tujia culture, we found that the local Tujia people’s knowledge of their traditional medicine has largely been assimilated into TCM and modern medicine. The traditional Tujia classification of medicines is poorly known, and although some traditional usages are still preserved, it is challenging to trace the cultural roots in the naming and classification of their herbal medicines.

### The shaping role of the ecological environment on traditional Tujia medicinal culture

The medicines used and the practices of ethnic doctors are directly related to the ecological environment in which they live. Generally, the medicines used by ethnic doctors, particularly local herbal doctors, are determined by the availability of medicinal resources near their homes or clinics. In China, the climate and ecological environments north and south of the Yangtze River differ significantly, leading to substantial differences in the traditional medicines used by ethnic groups in the north, such as the Mongolian (Badalahu et al., 2022), Hui (Chen et al., 2008a), and Uighur (Chen et al., 2008b), compared to those in the south, such as the Miao (Liu et al., 2023), Gelao (Liu et al., 2023), Zhuang (Xiao et al., 2021) and the Tujia people whom we studied. Our investigation of traditional medicines in the Gansu-Ningxia-Inner Mongolia border area and the Gelao traditional medicines in Guizhou revealed significant differences, except for a few widely distributed plants such as Plantago and dandelion. Most medicinal plants are distinct among these regions. For instance, common and widely used northern herbs such as ephedra, liquorice, and Astragalus are rarely seen in southern ethnic medicine. Conversely, information on orchidaceous and fern herbal medicines, which are common in southern regions, is scarce in arid northern areas. A classic example of this north‒south division in animal-derived medicines is our previous reports on the distinct regional usage of scorpions and centipedes (Xie et al., 2023a; Xie et al., 2022).

The ecology, climate, and cultural economy of the eastern Guizhou region are different from those of Hubei and Hunan, which are also part of the Wuling Mountains (Sun et al., 2013). This difference could be one of the reasons for the differences in traditional Tujia medical culture between the eastern Guizhou and Hubei-Hunan regions. For example, we found fewer than one-third of the 72 medicines classified as “lian” in Tongren, although most plant species in the Wuling Mountains are similarly distributed in both Guizhou and Hubei. There may also be influences from neighboring ethnic groups contributing to these differences.

### The impact and integration of socioeconomic and cultural development on traditional ethnic medicine

Ethnic medical culture largely relies on oral transmission and is a process enriched through generations, characterized by collectivity, orality, inheritance, and variability. In the transmission of ethnic medical culture, each generation imparts its knowledge and experiences to the next-generation. This process is inevitably influenced by the broader socioeconomic and cultural environment of the times. Mainstream cultural influences may impact ethnic medical culture to varying degrees (Wang and Xiang, 2012).

Our findings indicate that the application of traditional medicinal knowledge by the contemporary Tujia people has significantly evolved compared to that of their predecessors. They have integrated aspects of TCM and modern medical practices into their traditional medicinal knowledge and have passed this blended knowledge to the next-generation. Many younger Tujia practitioners have received systematic training in TCM or modern medicine, leading to divergent perspectives. Some believe in the unique efficacy of traditional Tujia medicine, while others are skeptical, citing perceived deficiencies in efficacy, hygiene, and safety. Consequently, the latter group is more inclined to adopt TCM or modern medical practices.

This generational shift reflects a broader trend in which traditional knowledge is being reinterpreted and adapted in light of contemporary medical understanding and societal advancements. As socioeconomic development progresses, the fusion of traditional and modern medical practices becomes more pronounced, reflecting a dynamic interplay between heritage and innovation. This not only highlights the resilience and adaptability of ethnic medical cultures but also underscores the importance of safeguarding these practices amidst ongoing cultural and scientific changes.

To safeguard Tujia traditional herbal knowledge, we recommend the establishment of a comprehensive digital database that documents the medicinal uses, preparation methods, and ecological details of local plants. This platform would serve as a crucial resource for both local communities and researchers. Additionally, integrating indigenous knowledge into the formal education system and providing financial support for herbal practitioners could foster greater engagement with traditional medicine. Community-based training programs aimed at younger generations should be developed to offer hands-on learning experiences in herbal medicine. These initiatives could be incorporated into local schools, community centers, and cultural institutions. Furthermore, cultural revitalization efforts, such as herbal medicine festivals and workshops, should be actively promoted to raise awareness and generate interest among youth.

### The role of ethnobotanical research in advancing local traditional ethnomedicine

Our research indicates that while eastern Guizhou Province, which is part of the Wuling Mountains and the birthplace of Tujia culture, has seen its traditional medical culture lose its distinct ethnic characteristics, the neighboring provinces of Hubei and Hunan have preserved and even developed their Tujia medical practices. This disparity is directly related to the efforts of ethnobotanists and pharmacological researchers. Enshi in Hubei serves as a prominent example of the development of traditional Tujia medicine, driven by research conducted by scholars from Hubei University for Nationalities (Xu, 2016) and Hubei University of Chinese Medicine (Sun, 2014). These scholars have systematically documented the traditional herbal medicines of the Tujia people in the Wuling Mountains and enhanced their traditional medical practices to form basic theories of Tujia medicine, such as three elements theory, five diagnostic methods, medicinal properties, and treatment principles. This illustrates the crucial role of ethnobotanical research in the preservation and advancement of traditional medical culture.

The traditional medical culture of Tujia in Tongren, Guizhou, urgently needs to be explored and protected by researchers. Our findings are just a starting point. The work of ethnobotanical research can provide the foundation for safeguarding and revitalizing traditional medical practices, ensuring that they remain a vital part of cultural heritage and continue to offer valuable medical insights. This research underscores the significance and impact of ethnobotanical studies in driving the development and preservation of traditional ethnic medicine.

### Modern pharmacological research on traditional Tujia herbal medicine

We conducted a literature analysis of the material basis, bioactivity, and applications of herbal medicines traditionally used by the Tujia people, including their use in traditional Chinese medicine (TCM) and among other ethnic minorities. Currently, 38 of these medicinal plants are included in the “Chinese Pharmacopoeia” as commonly used TCM herbs. Research on the material basis and bioactivity of these plants has varied. Additionally, we found that 113 of these plants are used in formulated medicines. This indicates that while only 38 of the Tujia medicinal plants are officially recognized in the Chinese Pharmacopoeia, at least 113 are in practical use as traditional Chinese medicines (Supplementary Table 3).

Regarding the use of Tujia traditional herbal medicine among other ethnic groups, we discovered a similar trend in the application of the same herbs across multiple ethnicities, especially for treating conditions such as injuries from falls, rheumatic pain, and other ailments. Many herbs with antibacterial and anti-inflammatory properties are widely used among different ethnic groups. There may be subtle differences in the applications of these herbs across ethnicities, likely due to variations in language and descriptive methods, making it challenging to distinguish these differences. Moreover, the usage and therapeutic range of these herbs are highly consistent across ethnic groups, with most treated ailments being explainable by both TCM theory and modern pharmacology. For instance, herbs used to treat snake bites are commonly believed to have heat-clearing and detoxifying effects in TCM, while these effects are attributed to the anti-inflammatory properties of modern medicine.

These findings not only validate the scientific basis of ethnomedicine but also reflect the mutual influence and complementarity between traditional Chinese medicine and modern medical science. This study provides crucial scientific evidence and practical guidance for future research and development of ethnomedicine. Additionally, it supports the preservation and transmission of these valuable intangible cultural heritages.

## Conclusion

In eastern Guizhou’s Tongren region, we collected information on 168 traditional medicinal plants used by the Tujia people. This information reflects the disease profile of the Tujia community in Tongren. With the advancement of the economy and medical technology, traditional Tujia medical culture is gradually disappearing. Our work provides primary data for the protection of Tujia medical culture and the development of traditional medicines in the region. However, during our investigation, we encountered challenges related to the accurate identification of plant species, which led to some inaccuracies and confusion in the information provided by the informants. Specifically, many informants were uncertain about the precise identification of certain medicinal plants. This confusion may stem from the loss of traditional knowledge over generations or the blending of different medicinal systems. These inaccuracies added complexity to our efforts in verifying the botanical origins and medicinal properties of the plants. To address this issue, we have emphasized the need for further validation of the origins, efficacy, and safety of these medicinal plants through additional research. Accurate identification of plant species is critical for ensuring the proper use and preservation of Tujia traditional medicine. In future studies, we will prioritize investigating the precise origins, material basis, bioactivity, safety, and clinical applications of the selected important ethnic medicines. This continued research will help to solidify the foundation for the preservation and proper utilization of Tujia traditional medical knowledge.

## Data Availability

The raw data supporting the conclusions of this article will be made available by the authors, without undue reservation.

## Abbreviations

HUF, The Utilization Frequency; NFSI, National Cultural Significance Index; FQI, Frequency Quotation Index; AI, Availability Index; FUI, Frequency of Use Index; PUI, Part Used Index; MFI, Multifunctional Use Index; CEI, Efficacy Index; DSI, Safety Index.
