# Supplementary material for: Medicinal plant use by the Tujia people in northeastern Guizhou, China: an ethnobotanical study
Source: Front Pharmacol. 2025 Mar 28;16:1522456. doi: 10.3389/fphar.2025.1522456 (PMC11985523; doi:10.3389/fphar.2025.1522456)
Supplement: Supplementary file 1 [file Table1.docx]

**Supplementary Table 1 Basic Information of the Research Area**

| **County** | **Geographical Coordinates** | **Population** | **Ethnic Groups/Religions** | **Healthcare Facilities** | **Major Towns** |
| --- | --- | --- | --- | --- | --- |
| Yinjiang Miao and Tujia Autonomous County | E 108°17′-108°48′;  N 27°35′-28°28′ | 294,490 | Tu Jia  Miao  Han | 385 healthcare institutions | Yangxi Town, |
|  |  |  |  |  | Banxi Town |
|  |  |  |  |  | Xinzhai Town |
| Yanhe Tujia autonomous County | E 108°03′-108°37′;  N 28°12′-29°05′ | 429,893 | Tu Jia  Miao  Dong  Ge Lao | 457 healthcare institutions | Qitan Town |
|  |  |  |  |  | Banchang Town |
|  |  |  |  |  | Huangtu Town |
| Sinan County | E 107°52′-108°27′;  N 27°31′-28°9′ | 457,745 | Tu Jia  Yi  Bai | 548 healthcare institutions | Yingwu Creek Town |
| Dejiang County | E 107°46′-108°00′;  N 28°00′-28°38′ | 393,400 | Tu Jia | 409 healthcare institutions | Fengxiang Creek Town |
|  |  |  |  |  | Qinglong Town |
|  |  |  |  |  | Hongqi Street |
| Shiqian County | E 107°44′-108°33′;  N 27°17′-27°42′ | 413,800 | Ge Lao  Miao  Tu Jia | 347 healthcare institutions | Tangshan Town |
