# Supplementary material for: Medicinal plant use by the Tujia people in northeastern Guizhou, China: an ethnobotanical study
Source: Front Pharmacol. 2025 Mar 28;16:1522456. doi: 10.3389/fphar.2025.1522456 (PMC11985523; doi:10.3389/fphar.2025.1522456)
Supplement: Supplementary file 2 [file Table2.docx]

**Supplementary Table 2 Catalog of Traditional Herbal Medicine for the Tujia Ethnics in Tongren**

| **Local Name** | **Scientific Name** | **Family** | **Part Used** | **Growth Form** | **Preparation** | **Medicinal Uses** | **Application Method** | **voucher number** |
| --- | --- | --- | --- | --- | --- | --- | --- | --- |
| Cong Bai/Huo Cong | *Allium fistulosum* L. | Liliaceae | Stem | Herbaceous | Fresh | Common cold, Diarrhea, Furunculosis | Decoction, Crushed for topical application | TJ-03-A-041 |
| Gan Shan Bian/Pan Long Qi | *Peliosanthes macrostegia* Hance | Liliaceae | Rhizome | Herbaceous | Dried | Rheumatic pain, Trauma, Abdominal bloating | Decoction | TJ-03-A-011 |
| Ma Gan Qi/Lu Yao | *Maianthemum japonicum* (A.Gray) LaFrankie | Liliaceae | Tuber and root | Herbaceous | Dried after scorching, Fresh | Rheumatism, Headache, Menstrual disorders, Trauma, Nutritive | Decoction, Crushed for topical application, Used in stew | TJ-03-A-039 |
| Ye Cong/Xiao Cong | *Allium macrostemon* Bunge | Liliaceae | Bulb | Herbaceous | Steamed then dried | Epigastric pain, Abdominal pain, Dysentery, Lumbar pain | Powdered for ingestion, Decoction | TJ-03-A-040 |
| Bai Zao Xiu/Deng Tai Qi | *Paris lancifolia* Hayata/P*orella japonica var. calcicola* M. Hara | Liliaceae | Rhizome | Herbaceous | Dried | Snake bites, Mumps, Trauma, Ulcers, Otitis | Powder mixed with vinegar for topical use, Egg white mixture for topical use, Human milk for ear drops | TJ-03-A-035 |
| Di Zhu/Tou Ding Zhu | *Trillium tschonoskii* Maxim. | Liliaceae | Stem, Root, Fruit | Herbaceous | Dried | Dizziness, Trauma, Bleeding injuries | Steamed egg, Alcohol infusion, Powder for topical application | TJ-03-A-037 |
| Wan Nian Qing | *Rohdea japonica* (Thunb.) Roth | Liliaceae | Root and rhizome | Herbaceous | Dried or fresh | Encephalitis, Throat pain, Edema, Snake bites, Trauma | Fresh juice, Decoction, Crushed with wine lees for topical use | TJ-03-A-144 |
| Huang Jing Can/Lao Hu Jiang | *Polygonatum* Mill. | Liliaceae | Rhizome | Herbaceous | Fully steamed and dried | Nutritive | Powdered for ingestion, Used in stew | TJ-03-A-038 |
| Yi Wo Qu/Tian Dong | *Asparagus* *cochinchinensis* (Lour.) Merr. | Liliaceae | Tuberous root | Herbaceous | Fully steamed and dried | Nutritive | Alcohol infusion, Decoction, Used in stew | TJ-03-A-010 |
| Zhu Gen Qi | *Disporopsis fuscopicta* Hance | Liliaceae | Rhizome | Herbaceous | Dried | Nutritive | Decoction | TJ-03-A-145 |
| Bai He | *Lilium brownii var. viridulum* Baker/*Cardiocrinum giganteum var. yunnanense* (Elwes) Stearn | Liliaceae | Bulb | Herbaceous | Blanching followed by drying | Pulmonary diseases | Decoction | TJ-03-A-044 |
| Ji Xiang Cao | *Reineckea carnea* (Andrews) Kunth | Liliaceae | Rhizome | Herbaceous | Dried | Trauma, Lumbar pain, Asthma | Crushed with wine for topical use, Alcohol infusion, Decoction | TJ-03-A-146 |
| Ma Ti Xiang/Zhi Zhu Xiang | *Valeriana jatamansi* Jones | Valerianaceae | Rhizome and root | Herbaceous | Dried or fresh | Stomach pain, Abdominal pain, Epigastric pain, Vomiting, Childhood nutritional disorders, Ulcers | Powdered for ingestion, Decoction, Fresh or dried crushed for topical use with vinegar | TJ-03-A-143 |
| Ban Bian Lian | *Lobelia chinensis* Lour. | Lobelia | Whole plant | Herbaceous | Dried or fresh | Insect and snake bites, High fever in children, Abdominal edema | Crushed for topical use and mixed with sweet wine, Decoction | TJ-03-A-042 |
| Si Kuai Wa/Si Da Tian Wang | *Lysimachia paridiformis* Franch. | Primulaceae | Rhizome |  | Dried or fresh | Trauma, Rheumatic pain, Childhood nutritional disorders | Alcohol infusion for internal and external use, Decoction | TJ-03-A-043 |
| Man Tian Xing | *Lysimachia congestiflora* Hemsl. | Primulaceae | Whole plant | Herbaceous | Dried | Urolithiasis, Hernia, Diarrhea, Ulcers, Burns, Snake bites | Decoction, Crushed for topical use | TJ-03-A-009 |
| Zhu Er Duo/Che Qian Cao | *Plantago asiatica* L. | Plantaginaceae | Whole plant | Herbaceous | Dried or fresh | Urinary disorders, Diarrhea, Gynecological disorders | Decoction, Fresh juice with rice water for ingestion | TJ-03-A-035 |
| Yi Mu Cao | *Leonurus japonicus* Houtt. | Lamiaceae | Whole plant | Herbaceous | Dried | Gynecological disorders/Infertility | Deco and cotion | TJ-03-A-142 |
| Shan Bo He/Chou Bo He | *Mentha canadensis* L. | Lamiaceae | Aerial parts | Herbaceous | Dried | Measles, Nasal congestion | Decoction, Powder for nasal application | TJ-03-A-141 |
| Deng Long Cao/Feng Lun Cao | *Trifolium polycephalum* Ser. | Lamiaceae | Whole plant | Herbaceous | Dried or fresh | Diarrhea, Trauma | Decoction, Fresh crushed for topical use | TJ-03-A-147 |
| Mao Yan Cao/Dao Du San | *Euphorbia helioscopia var. ceretana* Sennen | Euphorbiaceae | Whole plant | Herbaceous | Dried or fresh | Ulcers, Scabies, Coughing and asthma | Milky juice for topical use, Decoction | TJ-03-A-137 |
| Suan Pan Zi | *Glochidion puberum* (L.) Hutch. | Euphorbiaceae | Root | Herbaceous | Dried | Amenorrhea | Cooked with chicken eggs | TJ-03-A-139 |
| Han Xiu Cao | *Mimosa pudica f. glabrior* Benth. | Fabaceae | Whole plant | Herbaceous | Dried | Rheumatoid arthritis | Decoction | TJ-03-A-138 |
| Yun Shi | *Caesalpinia vesicaria* Lam. | Fabaceae | Seeds | Tree | Dried | Cold, Toothache, Snake bites | Decoction, Powder mixed with crushed cactus for topical use | TJ-03-A-140 |
| Huang Ge Teng | *Pueraria montana var. lobata* (Willd.) Maesen & S.M.Almeida ex Sanjappa & Predeep | Fabaceae | Root | Woody vine | Dried | Heatstroke, Measles, Diarrhea, Fever | Decoction | TJ-03-A-008 |
| Ye Wan Dou | *Lotus corniculatus* L. | Fabaceae | Whole plant | Root | Dried | Asthma and cough | Decoction | TJ-03-A-136 |
| Xue Teng | *Sargentodoxa cuneata* (Oliv.) Rehder & E.H.Wilson | Sargentodoxaceae | Stem | Vine | Sliced and dried | Lumbar and leg pain, Trauma | Decoction, Alcohol infusion | TJ-03-A-007 |
| Bai Yao/Qian Jin Teng | *Stephania herbacea* Gagnep. | Menispermaceae | Tuber and leaf | Herbaceous | Dried or fresh | Snake bites, Rheumatic pain, Abdominal pain, Ulcers | Fresh crushed for topical use, Decoction, Powdered for ingestion | TJ-03-A-030、4 |
| Di Ku Dan | *Tinospora sagittata* (Oliv.) Gagnep. | Menispermaceae | Tuber | Vine | Dried | Throat pain, Thrush, Abdominal pain, Diarrhea | Powdered and mixed with water | TJ-03-A-012 |
| Shan Wu Gui | *Stephania epigaea* H.S.Lo | Menispermaceae | Tuber | Vine | Sliced and dried, boiled and peeled | Gastrointestinal disorders | Powdered and taken with ginger tea | TJ-03-A-045 |
| Hai Jin Sha | *Lygodium japonicum* (Thunb.) Sw. | Lygodiaceae | Whole plant | Fern | Dried | Urolithiasis | Decoction | TJ-03-A-135 |
| Xiang Ya Cao | *Festuca brauniana* (Nees) Walp. | Poaceae | Stem and leaves | Herbaceous | Dried | Cold, Cough, Asthma, Diarrhea | Decoction | TJ-03-A-015 |
| Yan Mai | *Avena fatua subsp. nuda* (L.) Thell. | Poaceae | Whole plant | Herbaceous | Dried | Night sweats | Decoction | TJ-03-A-134 |
| Ma You | *Sesamum indicum* L. | Pedaliaceae | Seeds | Herbaceous | Oil extracted | Constipation, Lower back pain, Tinnitus | Consumed with honey | TJ-03-A-148 |
| Ku Gua | *Momordica charantia var. abbreviata* Ser. | Cucurbitaceae | Fruit | Herbaceous | Sliced and dried | High blood pressure | Used as tea | TJ-03-A-006 |
| Bai Wei Lian/Jin Gui Lian | *Schizocarpum guatemalense* Cogn. ex Donn.Sm./*Hemsleya chinensis var. longivillosa* (C.Y.Wu & Z.L.Chen) D.Z.Li | Cucurbitaceae | Tuber | Vine | Dried | Stomach pain, Coughing blood, Spitting blood, Ulcers | Decoction, Powder mixed with vinegar for topical use | TJ-03-A-005 |
| Ye Xi Gua/Dong Gu Zi | *Trichosanthes kirilowii* Maxim. | Cucurbitaceae | Fruit, Seeds | Vine | Dried | Cough, Coughing blood | Decoction | TJ-03-A-013 |
| Pa Di Hong Mao | *Chrysosplenium pilosum* Maxim. | Saxifragaceae | Whole plant | Herbaceous | Dried or fresh | Trauma | Alcohol infusion or crushed for topical use | TJ-03-A-133 |
| Yan Bai Cai | *Bergenia purpurascens* (Hook.f. & Thomson) Engl. | Saxifragaceae | Whole plant | Herbaceous | Dried or fresh | Pulmonary diseases | Decoction | TJ-03-A-130 |
| Sheng Jiang | *Zingiber officinale f. rubens* (Makino) M.Hiroe | Zingiberaceae | Rhizome | Herbaceous | Fresh | Cold, Skin diseases (hair loss), Motion sickness | Decoction, Sliced for topical use or rubbing | TJ-03-A-132 |
| Shan Jiang | *Alpinia japonica* (Thunb.) Miq. | Zingiberaceae | Rhizome | Herbaceous | Dried or fresh | Abdominal pain, Diarrhea, Cold, Stomach pain | Decoction, Powdered for ingestion | TJ-03-A-046 |
| Jin Si Cao | *Polytrichum formosum* Hedw. | Polytrichaceae | Whole plant | Moss | Dried or fresh | Trauma, Bleeding injuries | Powdered and mixed with wine, Alcohol infusion, Crushed for topical use | TJ-03-A-131 |
| Tian Ji Huang/Di Er Cao | *Hypericum japonicum* Thunb. | Hypericaceae | Whole plant | Herbaceous | Dried or fresh | Snake bites | Decoction, Fresh crushed for topical use | TJ-03-A-004 |
| Xiao Niu Xi/Dui Ye Qi | *Chloranthus serratus* (Thunb.) Roem. & Schult. | Chloranthaceae | Root or whole plant | Shrub | Dried or fresh | Trauma, Joint pain, Snake bites | Decoction or alcohol infusion, Crushed for topical use | TJ-03-A-129 |
| Si Ye Xi Xin/Si Kuai Wa | *Chloranthus serratus* (Thunb.) Roem. & Schult. | Chloranthaceae | Whole plant | Shrub | Dried or fresh | Childhood convulsions, Lumbar pain | Decoction, Alcohol infusion | TJ-03-A-128 |
| Si Kuai Wa | *Chloranthus spicatus* (Thunb.) Makino/*Chloranthus henryi* Hemsl. | Chloranthaceae | Whole plant | Shrub | Dried or fresh | Diarrhea, Rheumatic pain, Epigastric pain | Decoction | TJ-03-A-033 |
| Zi Hua Di Ding | *Viola diamantiaca* Nakai/*Viola betonicifolia var. cordifolia* Hara | Violaceae | Whole plant | Herbaceous | Dried or fresh | Ulcers, Snake bites, Bleeding injuries | Decoction, Fresh crushed for topical use | TJ-03-A-003 |
| Luan Zi Cao/Yan Song | *Sedum elatinoides* Franch./S*edum lineare* Thunb. | Crassulaceae | Whole plant | Herbaceous | Dried or fresh | Diarrhea, Burns | Decoction, Fresh crushed for topical use | TJ-03-A-127 |
| La Jiao Cao | *Sedum oligospermum* Maire | Crassulaceae | Whole plant | Herbaceous | Dried or fresh | Trauma, Headache, Ulcers | Decoction, Fresh crushed for topical use | TJ-03-A-149 |
| Wa Song/Wa Lian Hua | *Cotyledon elizae* A.Berger ex Raym.-Hamet | Crassulaceae | Whole plant | Herbaceous | Dried or fresh | Diarrhea, Bloody stool, Hemorrhoids, Ulcers | Decoction, Powder for topical use | TJ-03-A-047 |
| Ba Bao Cao/Huo Yan Cao | *Hylotelephium erythrostictum* (Miq.) H.Ohba | Crassulaceae | Whole plant | Herbaceous | Dried or fresh | Sore throat, Shingles, Trauma, Burns, Menstrual disorders, Eye diseases | Decoction or fresh crushed for topical use, Fresh juice for eye drops | TJ-03-A-016 |
| Da Bu Si/Luo Di Sheng Gen | *Bryophyllum pinnatum* (Lam.) Oken | Crassulaceae | Whole plant or root | Herbaceous | Fresh | Bleeding injuries, Otitis, Ulcers | Crushed for topical use, Juice for ear drops | TJ-03-A-126 |
| Dou Ban Cai | *Sedum majus* (Hemsl.) Migo | Crassulaceae | Whole plant | Herbaceous | Dried or fresh | Ulcers, Trauma, Burns, Menstrual disorders | Crushed for topical use, Decoction | TJ-03-A-032 |
| Da Bu Si | *Sedum chauveaudii* Raym.-Hamet | Crassulaceae | Whole plant | Herbaceous | Mostly fresh | Insect and snake bites, Ulcers | Crushed for topical use | TJ-03-A-150 |
| Fei Cai | *Sedum oligospermum* Maire | Crassulaceae | Whole plant | Herbaceous | Mostly fresh | Trauma, Burns, Insect and snake bites | Crushed for topical use | TJ-03-A-125 |
| Tu San Qi | *Phedimus aizoon* (L.) 't Hart | Crassulaceae | Rhizome | Herbaceous | Dried | Various bleeding conditions, Trauma | Decoction, Alcohol infusion |  |
| Tu Dang Shen | *Campanumoea javanica* Blume | Campanulaceae | Root | Herbaceous | Dried or fresh | Indigestion, Food retention, Nutritive | Decoction, Used in stew with pork feet | TJ-03-A-048 |
| Ye Dang Shen | *Codonopsis javanica subsp. japonica* (Maxim. ex Makino) Lammers | Campanulaceae | Root | Herbaceous | Dried | Nutritive | Used in stew | TJ-03-A-002 |
| Ai Cao | *Artemisia lavandulifolia* Salisb./*Artemisia indica* Willd./*Artemisia argyi* H.Lév. & Vaniot | Asteraceae | Stem and leaves | Herbaceous | Dried, leaves rubbed into fluff | Sterilization, Expels wind and dampness, Treats stomach ailments | Moxibustion, Decoction, Bath infusion | TJ-03-A-001 |
| Jiu Li Guang | *Senecio scandens* Buch.-Ham. ex D.Don | Asteraceae | Aerial parts | Herbaceous | Fresh or dried | Throat pain, Skin diseases, Snake bites | Decoction, Decoction for bathing or topical application | TJ-03-A-122 |
| Cang Er Zi/Zhan E Zi/Cang Er Zi | *Xanthium strumarium* L. | Asteraceae | Fruit | Herbaceous | Dried | Headache, Leg pain, Diarrhea, Nasal congestion, Skin itching | Decoction, Powder for nasal application, Decoction for washing or topical application | TJ-03-A-124 |
| Shan Luo Bo 、 Ye Luo Bo | *Parasenecio forrestii* W.W.Sm. & J.Small | Asteraceae | Tuber | Herbaceous | Dried | Abdominal bloating, Cough | Decoction | TJ-03-A-123 |
| Hong Chai Hu 、 Yi Zhi Huang Hua | *Solidago decurrens* Lour. | Asteraceae | Whole plant | Herbaceous | Dried | Throat pain, Ulcers, Palmoplantar pustulosis | Decoction, Fresh crushed for topical use, Decoction for bathing | TJ-03-A-031 |
| Da Huo Cao/Qing Ming Cao | *Psychrophyton buchanani* (Kirk) Anderb. | Asteraceae | Whole plant | Herbaceous | Dried or fresh | Throat pain, Burns, Snake bites | Rinse with rice water after soaking, Powder mixed with vegetable oil for topical use, Fresh crushed for topical use | TJ-03-A-049 |
| Ye Yan | *Carpesium abrotanoides* L. | Asteraceae | Whole plant | Herbaceous | Dried | Various bleeding conditions, Insect bites, Ulcers | Decoction, Alcohol infusion for topical use | TJ-03-A-121 |
| Dong Feng Cai | *Aster scaber* Thunb. | Asteraceae | Whole plant | Herbaceous | Mostly fresh | Insect and snake bites, Trauma | Crushed for topical use | TJ-03-A-151 |
| Tu San Qi | *Gynura japonica* (Thunb.) Juel | Asteraceae | Root, stem leaves | Herbaceous | Dried or fresh | Trauma, Bleeding, Dysmenorrhea | Powdered and mixed with wine, Crushed for topical use, Decoction | TJ-03-A-120 |
| Shen Jiao Cao/Cui Yun Cao | *Selaginella uncinata* (Desv.) Spring | Selaginellaceae | Whole plant | Fern | Dried | Jaundice, Pulmonary hemorrhage, Gonorrhea, Cramps, Rheumatism | Decoction, Alcohol infusion | TJ-03-A-152 |
| Shi Shang Bai/Yan Juan Bai | *Selaginella involvens* (Sw.) Spring | Selaginellaceae | Whole plant | Fern | Dried | Jaundice, Diarrhea, Bleeding, Cancer | Decoction or mixed with alcohol, Crushed or powdered for topical use | TJ-03-A-119 |
| Jiu Tou Shi Zi | *Peristrophe japonica* (Thunb.) Bremek. | Acanthaceae | Whole plant | Herbaceous | Dried or fresh | Trauma, Snake bites, Childhood convulsions, Cold, Fever, Menstrual disorders | Decoction, Crushed for topical use | TJ-03-A-153 |
| Xiao Qing Cao/Gan Ji Cao | *Justicia procumbens* Blume | Acanthaceae | Whole plant | Herbaceous | Dried | Childhood nutritional disorders, Liver disease, Edema | Decoction | TJ-03-A-154 |
| Yan Pi Pa/Yan Ju Hua | *Briggsia latisepala* Chun ex K.Y.Pan | Gesneriaceae | Whole plant | Herbaceous | Dried or fresh | Dysentery, Asthma, Bleeding injuries | Decoction, Powdered for topical use or fresh crushed for topical use | TJ-03-A-050 |
| Shi Diao Lan | *Lysionotus pauciflorus* Maxim. | Gesneriaceae | Whole plant | Herbaceous | Dried | Trauma, Burns, Pain | Alcohol infusion, Powder mixed with essential oil for topical application | TJ-03-A-118 |
| Jia Tian Ma | *Corallorhiza trifida var. trifida* | Orchidaceae | Whole plant | Herbaceous | Boiled and dried | Rheumatic pain, Fever, Dizziness | Decoction | TJ-03-A-156 |
| Shan Ci Gu/Ye Bai Ji/Bai Mao Gu | *Cremastra appendiculata* (D.Don) Makino | Orchidaceae | Pseudobulb | Herbaceous | Dried or fresh | Ulcers, Trauma, Tumors | Crushed for topical use, Decoction | TJ-03-A-155 |
| Huo Xue Zhu/Mao Suan Gu | *Pleione bulbocodioides* (Franch.) Rolfe | Orchidaceae | Pseudobulb | Herbaceous | Boiled thoroughly and dried | Ulcers, Snake bites, Centipede bites | Crushed mixed with vinegar for topical use | TJ-03-A-117 |
| Xiang Long Cao | *Pogonia japonica* Rchb.f. | Orchidaceae | Whole plant | Herbaceous | Mostly fresh | Snake bites | Crushed for topical use | TJ-03-A-051 |
| Fei Xing Cao | *Goodyera schlechtendaliana f. similis* (Blume) Makino | Orchidaceae | Whole plant | Herbaceous | Dried or fresh | Pulmonary tuberculosis, Bronchitis | Decoction | TJ-03-A-157 |
| Shuang Shen Cao | *Habenaria davidii* Franch. | Orchidaceae | Tuber | Herbaceous | Steamed and dried | Hernia, Impotence, Urolithiasis | Decoction | TJ-03-A-017 |
| Shuang Shen Can | *Habenaria dentata* (Sw.) Schltr. | Orchidaceae | Tuber | Herbaceous | Dried or fresh | Hernia, Ulcers | Cooked with pork testicles, Crushed for topical use | TJ-03-A-115 |
| Yan Shi Hu | *Bulbophyllum nutans* (Thouars) Thouars | Orchidaceae | Whole plant | Herbaceous | Dried or fresh | Pulmonary diseases, Trauma | Decoction, Crushed for topical use | TJ-03-A-158 |
| Lian Huan Cao | *Calanthe discolor var. discolor* | Orchidaceae | Whole plant | Herbaceous | Dried | Hemorrhoids | Powder mixed with vegetable oil for topical use | TJ-03-A-116 |
| Hong Hua Liao | *Polygonum runcinatum* Buch.-Ham. | Polygonaceae | Whole plant, leaves, root | Herbaceous | Dried or fresh | Lower back pain, Mastitis, Burns, Gunshot wounds | Alcohol infusion, Crushed mixed with lees for topical use, Powder mixed with tung oil for topical use, Fresh crushed for topical use | TJ-03-A-052 |
| Zhu Sha Qi | *Pleuropterus ciliinervis* Nakai | Polygonaceae | Tuber | Herbaceous | Sliced and dried or fresh | Diarrhea, Abdominal pain, Burns, Trauma | Powder for ingestion, Powder mixed with vegetable oil for topical application, Powder or fresh crushed for topical use | TJ-03-A-114 |
| Niu Dai Huang/Jin Da Huan | *Rumex crispus subsp. littoreus* (Hardy) Akeroyd | Polygonaceae | Root or leaf | Herbaceous | Dried or fresh | Constipation, Menstrual disorders, Coughing blood, Childhood impetigo, Burns | Decoction mixed with alcohol, Cooked in meat or soup, Fresh crushed for topical use, Powder mixed with sesame oil for topical use | TJ-03-A-053 |
| She Dao Tui | *Polygonum perfoliatum* L. | Polygonaceae | Whole plant | Herbaceous | Dried or fresh | Snake bites, Ulcers | Crushed for topical use or rinsed with rice water, Powder mixed with borneol and sesame oil for topical application | TJ-03-A-056 |
| Hou Er Qi | *Bistorta vivipara* (L.) Gray | Polygonaceae | Rhizome | Herbaceous | Dried | External bleeding, Abdominal pain, Diarrhea, Joint pain | Decoction, Alcohol infusion | TJ-03-A-055 |
| Xue Li Mei/Qing Yu Dan | *Gentiana rhodantha* Franch. | Gentianaceae | Whole plant | Herbaceous | Dried or fresh | Jaundice, Ulcers, Snake bites, Coughing blood | Rice water decoction, Decoction, Fresh crushed for topical use, Powder mixed with sweet wine for ingestion | TJ-03-A-112 |
| Xiao Long Dan/Tu Long Dan | *Halenia corniculata* (L.) Cornaz | Gentianaceae | Root or whole plant | Herbaceous | Dried | Heatstroke, Dizziness, Abdominal pain | Root cooked with meat, Whole plant decoction | TJ-03-A-111 |
| Yan Long Dan | *Gentiana squarrosa* Ledeb. | Gentianaceae | Whole plant | Herbaceous | Dried or fresh | Ulcers, Gastroenteritis | Powder mixed with oil for topical use, Decoction | TJ-03-A-113 |
| Dui Yue Cao/Zhu Ye Qi | *Vincetoxicum pycnostelma* Kitag. | Asclepiadaceae | Whole plant, root, and rhizome | Herbaceous | Dried or fresh | Trauma, Rheumatic pain, Vomiting, Snake bites | Decoction, Alcohol infusion, Fresh crushed for topical use | TJ-03-A-054 |
| Hei Gu Teng/Hei Hu Teng | ­­—— | Asclepiadaceae | Stem | Vine | Dried or fresh | Trauma, Rheumatic pain, Snake bites | Decoction, Alcohol infusion, Crushed for topical use | TJ-03-A-110 |
| Nai Jiang Teng | *Cynanchum rostellatum* (Turcz.) Liede & Khanum | Asclepiadaceae | Whole plant | Vine | Dried or fresh | Insect and snake bites, External bleeding, Childhood nutritional disorders | Crushed for topical use, Powder mixed with warm water and sugar for ingestion | TJ-03-A-030 |
| Da Feng Cao | *Ranunculus bungei* Steud. | Verbenaceae | Whole plant | Herbaceous | Dried | Leucorrhea, Insecticidal | Decoction | TJ-03-A-159 |
| Tu Ren Shen | *Talinum paniculatum* (Jacq.) Gaertn. | Portulacaceae | Root | Herbaceous | Dried or fresh | Nutritive | Cooked with meat | TJ-03-A-108 |
| Si Liang Ma | *Asarum sieboldii* Miq. | Aristolochiaceae | Whole plant | Herbaceous | Dried or fresh | Abdominal pain, Snake bites, Common cold with headache | Mixed with rice water for topical application, Decoction | TJ-03-A-057 |
| Ma Ti Xiang/Pen Cao | *Saruma henryi* Oliv. | Aristolochiaceae | Whole plant | Vine | Dried | Abdominal pain, Ulcers | Powder for ingestion, Crushed for topical use | TJ-03-A-109 |
| Ma Ti Xiang/Ma Xi Xin | *Asarum macranthum* Hook.f. | Aristolochiaceae | Whole plant | Herbaceous | Dried | Common cold, Abdominal pain | Decoction | TJ-03-A-018 |
| She Shen | *Aristolochia tubiflora* Dunn | Aristolochiaceae | Root, whole plant | Vine | Mostly fresh | Insect and snake bites, Diarrhea, Ulcers, High blood pressure | Decoction, Crushed for topical use | TJ-03-A-060 |
| Ma Sang | *Coriaria nepalensis* Wall. | Coriariaceae | Bark | Shrub | Dried or fresh | Toothache, Fractures | Decoction, Crushed and wrapped around the affected area | TJ-03-A-059 |
| Po Xue Zi/Lao Guan Cao | *Geranium wilfordii* Maxim. | Geraniaceae | Whole plant | Herbaceous | Dried | External bleeding, Rheumatic pain, Urticaria | Powder for topical hemostasis, Decoction | TJ-03-A-062 |
| Niu Jiao Qi/Tie Po Luo/Guai Zi Qi | *Beesia calthifolia* Ulbr. | Ranunculaceae | Rhizome | Herbaceous | Dried or fresh | Thrush, Throat pain, Joint pain, Trauma, Snake bites | Decoction, Fresh crushed for topical use | TJ-03-A-058 |
| Sui Gu Cao/Ye Wu Tou | *Aconitum sinomontanum* Nakai | Ranunculaceae | Root | Herbaceous | Dried | Rheumatic pain, Trauma, Snake bites, Abdominal pain | Decoction or alcohol infusion | TJ-03-A-061 |
| Lao Hu Xiang/Shi Huang Cao | T*halictrum aquilegiifolium var. sibiricum* Regel & Tiling/*Thalictrum microgynum* Lecoyer ex Oliv. | Ranunculaceae | Whole plant | Herbaceous | Dried | Jaundice, Diarrhea | Decoction | TJ-03-A-029 |
| Tian Qu Zi/Qian Nian Lao Shu Shi | *Semiaquilegia adoxoides* Makino | Ranunculaceae | Whole plant, root | Herbaceous | Dried or fresh | Snake bites, Pulmonary diseases | Fresh crushed for topical use, Root cooked with pork stomach | TJ-03-A-107 |
| Ye Mian Hua/Da Po Wan Wan Hua | *Anemone vitifolia* Buch.-Ham. ex DC. | Ranunculaceae | Root and leaves | Herbaceous | Dried | Scabies, Parasitic infections | Powder mixed with vinegar for topical application, Decoction | TJ-03-A-019 |
| Leng Fan Tuan/Xiang Xue Teng | *Kadsura heteroclita* (Roxb.) Craib | Schisandraceae | Vine stem, root | Vine | Sliced and dried | Abdominal pain, Flatus, Lower back pain, Dysmenorrhea | Decoction, Alcohol infusion | TJ-03-A-160 |
| Da Hui | *Illicium henryi* Diels | Magnoliaceae | Fruit, root, root bark | Tree | Dried | Joint and lower back pain | Decoction | TJ-03-A-028 |
| Leng Fan Tuan/Xiao Xiang Teng | *Schisandra* Michx. | Magnoliaceae | Root, stem leaves | Vine | Dried or fresh | Trauma, Fractures | Decoction, Crushed for topical use | TJ-03-A-063 |
| Ba Yue Zha/Ba Yue Gua | *Akebia quinata* (Thunb. ex Houtt.) Decne./*Akebia trifoliata* (Thunb.) Koidz. | Lardizabalaceae | Fruit | Vine | Boiled then dried | Kidney stones, Diarrhea | Decoction | TJ-03-A-106 |
| Yi Zhi Jian | *Ophioglossum vulgatum var. reticulatum* (L.) D.C.Eaton | Ophioglossaceae | Whole plant | Herbaceous | Dried or fresh | Snake bites | Chewed for topical application, Decoction | TJ-03-A-104 |
| Jian Zhong Xiao/Mu Zhu Teng | *Ampelopsis delavayana* Planch. ex Franch./*Ampelopsis delavayana var. setulosa* (Diels & Gilg) C.L.Li | Vitaceae | Root, root bark | Vine | Dried, fresh | Trauma, Fractures, Rheumatic pain | Decoction | TJ-03-A-064 |
| Shui Chang Shan/Yi Zhu Xiang | *Anotis urophylla* (Wall. ex Wight & Arn.) Hook.f. | Rubiaceae | Aerial parts | Herbaceous | Dried or fresh | Eye inflammation, Skin diseases | Decoction for eye wash, Fresh crushed for topical use | TJ-03-A-103 |
| Ci Li | *Rosa roxburghii* Tratt. | Rosaceae | Fruit | Woody vine | Dried, crushed | Sexual dysfunction, Infertility | Alcohol infusion, Decoction | TJ-03-A-105 |
| Lu Bian Huang/Ma Bian Cao | *Geum aleppicum* auct. | Rosaceae | Aerial parts | Herbaceous | Dried, cut | Fever reduction, Toothache, Inflammation | Decoction | TJ-03-A-027 |
| Di Feng Zi | *Potentilla freyniana var. sinica* Migo | Rosaceae | Rhizome |  | Dried or fresh | Diarrhea, Uterine bleeding, External bleeding | Decoction, Powder for ingestion, Topical application | TJ-03-A-065 |
| Hong Zi | *Pyracantha pyracantha* (L.) Voss | Rosaceae | Fruit, leaves, root | Shrub | Dried | Diarrhea, Ulcers | Fruit decoction, Fresh leaves crushed for topical use | TJ-03-A-101 |
| Shi Jie Mei | *Rosa saturata* Baker | Rosaceae | Root | Shrub | Dried | Menstrual disorders | Decoction | TJ-03-A-161 |
| Wu Pao | *Rubus setchuenensis* Bureau & Franch. | Rosaceae | Root | Shrub | Dried | Hemoptysis, Menstrual disorders | Decoction | TJ-03-A-102 |
| Zai Yang Pao | *Rubus corchorifolius* L.f. | Rosaceae | Fruit, Root | Shrub | Dried or fresh | Nocturnal emissions, Burns | Decoction, Crushed for topical use | TJ-03-A-162 |
| Qiu Hai Tang | *Begonia grandis* Dryand. | Begoniaceae | Rhizome | Herbaceous | Dried | Hematochezia, Metrorrhagia, Hernia, Rheumatic pain | Decoction | TJ-03-A-163 |
| Jin Yin Hua | *Urtica japonica* Thunb. | Caprifoliaceae | Flowers, Stem | Vine | Dried | Inflammation | Tea infusion | TJ-03-A-100 |
| Jie Gu Cao | *Sambucus javanica* Reinw. ex Blume | Caprifoliaceae | Root or whole plant | Herbaceous | Dried or fresh | Trauma, Ulcers | Crushed for topical use, Decoction, Mixed with egg white for topical application | TJ-03-A-020 |
| Zhe Er Gen/Yu Xing Cao | *Houttuynia cordata* Thunb. | Saururaceae | Whole plant | Herbaceous | Mostly fresh | Insect and snake bites, Diarrhea | Crushed for topical use, Decoction | TJ-03-A-164 |
| Xiang Cai | *Eryngium foetidum* L. | Apiaceae | Whole plant/Seeds | Herbaceous | Fresh or dried | Common cold, Skin itching | Decoction, Bathing or topical application | TJ-03-A-026 |
| Tu Dang Gui/Ye Dang Gui | *Angelica decursiva* (Miq.) Franch. & Sav. | Apiaceae | Root | Herbaceous | Dried, fresh | Cough, Cold, Skin diseases | Decoction, Fresh crushed for topical use | TJ-03-A-066 |
| Du Huo | *Aralia cordata* Thunb. | Apiaceae | Root and rhizome | Herbaceous | Dried | Wind-cold common cold, Rheumatic pain | Decoction | TJ-03-A-165 |
| E Jiao Ban | *Pimpinella diversifolia* DC. | Apiaceae | Whole plant | Herbaceous | Dried or fresh | Snake bites | Crushed juice for ingestion and topical application | TJ-03-A-099 |
| Jia Zi Cao/Zhan Shen Cao | *Torilis scabra* DC. | Apiaceae | Fruit and root | Herbaceous | Dried | Parasitic diseases, Food poisoning | Decoction | TJ-03-A-167 |
| tu dang gui | *Angelica biserrata* (R.H.Shan & Yuan) C.Q.Yuan & R.H.Shan | Apiaceae | Root | Herbaceous | Dried or steamed and dried | Nutritive | Decoction | TJ-03-A-166 |
| E Can | *Anthriscus sylvestris* (L.) Hoffm. | Apiaceae | Root | Herbaceous | Dried or fresh | Nutritive, Various pains, Thrush | Decoction, Powder for oral suspension, Crushed juice for topical application | TJ-03-A-067 |
| Yi Zhi Bi/Guan Yin Lian | *Balanophora involucrata var. gracilis* Hook.f. | Balanophoraceae | Whole plant | Fungus | Dried | Bleeding, Hemorrhoids | Cooked with meat, Powder for topical application | TJ-03-A-098 |
| Bai Hua Cai/Sui Mi Cai | *Cardamine leucantha* (Tausch) O.E.Schulz | Brassicaceae | Root | Herbaceous | Dried | Chronic cough, Pertussis | Decoction | TJ-03-A-097 |
| Shi Song/Qing Si Long | *Lycopodium japonicum* Thunb. | Lycopodiaceae | Whole plant | Herbaceous | Dried | Headache, Diarrhea, Fever | Decoction | TJ-03-A-025 |
| Shi Suan/Lao Ya Suan | *Lycoris aurea* (L'Hér.) Herb./*Lycoris radiata* (L'Hér.) Herb. | Amaryllidaceae | Bulb | Herbaceous | Dried, fresh | Furunculosis, Burns, Eczema | Fresh slices for topical application, Crushed with egg white for topical use | TJ-03-A-096 |
| Huang Yao Zi | *Dioscorea bulbifera* L. | Dioscoreaceae | Tuber | Vine | Dried or fresh | Hemoptysis, Pertussis, Thyroid enlargement, Snake bites, Hernia | Decoction, Crushed with wine for topical use | TJ-03-A-070 |
| Zhu Sha Lian | *Dioscorea cirrhosa* Lour. | Dioscoreaceae | Tuber | Vine | Dried | Various bleeding conditions, Snake bites, Diarrhea | Decoction, Powder for internal or topical use | TJ-03-A-095 |
| Jiu Gen Suo | *Lepisorus miyoshianus* (Makino) Fraser-Jenk. & Subh.Chandra | Polypodiaceae | Whole plant | Fern | Dried | Trauma, Lower back pain, Childhood convulsions | Decoction, Sugar mixed decoction for oral intake | TJ-03-A-069 |
| Wo Long Cao | *Pyrrosia angustissima* (Giesenh. ex Diels) Tagawa & K.Iwats. | Polypodiaceae | Whole plant | Fern | Dried or fresh | Edema, Gonorrhea, Gynecological disorders, Trauma | Decoction, Crushed for topical use | TJ-03-A-094 |
| Long Xu Cao/Xian Ren Tou Fa | *Usnea longissima* Ach. | Usneaceae | Whole organism | Lichen | Dried | Tuberculosis, Rheumatic pain, Burns, Fractures | Decoction mixed with alcohol, Decoction, Powder mixed with oil for topical application or crushed for topical use | TJ-03-A-068 |
| Yan Shua Zi/Shua Zhu Cao | *Psilotum nudum* (L.) P.Beauv. | Psilotaceae | Whole plant | Fern | Dried | Trauma, External bleeding, Rheumatic pain | Alcohol infusion, Crushed for topical use | TJ-03-A-090 |
| Hong Nan Xing/Gou Zhao Nan Xing | *Arisaema fargesii* Buchet | Araceae | Tuber | Herbaceous | Dried | Stroke, Childhood convulsions, Rheumatic pain, Snake bites | Powder for oral intake mixed with sugar, Decoction, Topical application | TJ-03-A-091 |
| Shi Chang Pu/Shui Chang Pu | *Acorus calamus* L. | Araceae | Rhizome | Herbaceous | Dried or fresh | Various pains, Snake bites, Asthma, Coma | Decoction, Crushed and heated for topical use | TJ-03-A-089 |
| Ban Jie Lan/Xue Li Jian | *Arisaema decipiens* Schott | Araceae | Tuber | Herbaceous | Dried or fresh | Various pains, Ulcers | Alcohol infusion, Crushed for topical use | TJ-03-A-088 |
| San Bu Tiao/Ma Yu Zi | *Pinellia ternata* (Thunb.) Makino | Araceae | Tuber | Herbaceous | Dried or fresh | Cough with phlegm, Headache, Snake bites | Decoction, Crushed for topical use | TJ-03-A-024 |
| Tie Si Cao/Tie Xian Cao | *Adiantum capillus-veneris* L. | Pteridaceae | Whole plant | Fern | Dried | Difficulty urinating, Burns, Snake bites, Trauma | Decoction, Powder mixed with oil for topical application | TJ-03-A-092 |
| Yan Feng Teng/Shang Shu Wu Gong | *Handroanthus serratifolius* (Vahl) S.O.Grose | Araliaceae | Vine stem | Vine | Dried or fresh | Rheumatic pain, Eczema, Snake bites | Decoction or alcohol bath or topical application, Fresh crushed for topical use | TJ-03-A-086 |
| Ci Lao Bao/Que Er Bu Zhan | *Aralia elata* (Miq.) Seem. | Araliaceae | Bark, young shoots | Shrub | Mostly fresh | Fractures, Trauma, Edema | Crushed for topical use, Cooked with meat | TJ-03-A-093 |
| Zhu Jie Ren Shen | *Panax pseudoginseng subsp. himalaicus* H.Hara | Araliaceae | Rhizome | Shrub | Dried | Bleeding, Hemorrhoids, Ulcers | Decoction, Powder for topical use | TJ-03-A-087 |
| Kou Zi Qi | *Panax bipinnatifidus* Seem. | Araliaceae | Rhizome | Herbaceous | Dried | Trauma, Various bleeding conditions | Decoction | TJ-03-A-071 |
| Shan He Ye/Yi Wan Shui | *Diphylleia sinensis* H.L.Li | Berberidaceae | Rhizome | Herbaceous | Dried or fresh | Trauma, Rheumatic pain, Ulcers, Abdominal pain | Alcohol infusion for internal and external use, Fresh crushed for topical use, Powder for ingestion | TJ-03-A-083 |
| Ci Huang Lian/San Ke Zhen | *Berberis julianae* C.K.Schneid. | Berberidaceae | Stem, root | Shrub | Sliced and dried | Eye inflammation, Abdominal pain, Diarrhea | Decoction | TJ-03-A-082 |
| He Ye Lian | *Dysosma versipellis* (Hance) M.Cheng ex T.S.Ying | Berberidaceae | Rhizome | Herbaceous | Dried or fresh | Trauma, Snake bites, Ulcers, Shingles | Fresh crushed for topical use, Powder mixed with vinegar for topical use | TJ-03-A-081 |
| Ji Wei Cao | *Pedicularis chinensis* Maxim. | Scrophulariaceae | Whole plant | Herbaceous | Dried | Diarrhea, Rheumatic joint pain, Ulcers | Powder for ingestion, Alcohol infusion, Topical application | TJ-03-A-168 |
| Tian Guan Zi/Chao Tian Guan | *Osbeckia crinita* Benth. ex Naudin | Melastomataceae | Root, whole plant | Shrub | Dried | Metrorrhagia, Diarrhea, Thrush | Decoction | TJ-03-A-084 |
| She Bu Jian/Tai Jue | *Sceptridium ternatum* (Thunb.) Lyon | Ophioglossaceae | Whole plant | Fern | Dried | Eye inflammation, Cough, Ulcers, Snake bites, Fever in children | Decoction, Fresh crushed for topical use, Powder for ingestion, Steamed with egg | TJ-03-A-023 |
| Yi Duo Yun | *Botrypus virginianus*  (L.) Holub | Ophioglossaceae | Whole plant | Fern | Dried or fresh | Cough, Ulcers, Snake bites | Decoction, Fresh crushed for topical use | TJ-03-A-072 |
| Tu Huang Lian/Duan Chang Cao | *Corydalis aurea* Willd. | Papaveraceae | Whole plant | Herbaceous | Dried | Diarrhea, Gonorrhea | Decoction | TJ-03-A-080 |
| Shui Huang Lian/Yi Dian Xue | *Eomecon chionantha* Hance | Papaveraceae | Root rhizome | Herbaceous | Dried | Snake bites, Lower back pain, Gonorrhea, Ulcers | Crushed for topical use, Alcohol infusion, Powder mixed with oil for topical application | TJ-03-A-075 |
| Shui Hu Lu | *Eichhornia crassipes* (Mart.) Solms | Pontederiaceae | Whole plant or root | Aquatic herbaceous | Dried or fresh | Kidney inflammation, Ulcers | Decoction, Fresh crushed for topical use | TJ-03-A-079 |
| Yuan Wei | *Iris tectorum* Maxim. | Iridaceae | Rhizome | Herbaceous | Dried or fresh | Rabies, Rheumatism, Trauma, Parasitic infections | Decoction, Bath infusion, Crushed for topical use | TJ-03-A-085 |
| Bian Zhu Gen | *Iris japonica* Thunb. | Iridaceae | Rhizome | Herbaceous | Dried or fresh | Trauma, Snake bites, Toothache | Decoction (mixed with children's urine), Crushed for topical use, Boiled egg infusion | TJ-03-A-074 |
| Jian Xue Fei/San Bai Bang | *Toddalia asiatica* (L.) Lam. | Rutaceae | Root, root bark | Tree | Dried | Various pains, External bleeding | Decoction, Alcohol infusion, Powder for topical use | TJ-03-A-073 |
| Xiang Ye Zi/Wu Yao | —— | Lauraceae | Root | Tree | Dried or fried with bran | Pain due to intestinal gas, Abdominal pain, Incontinence | Decoction | TJ-03-A-078 |
| Ba Zhao Jin Long/Kai Hou Jian | *Ardisia crenata* Sims/*Ardisia crispa* (Thunb.) A.DC. | Myrsinaceae | Root bark | Shrub | Dried | Inflammatory pain, Snake bites, Trauma | Decoction, Alcohol infusion, Chewed fresh | TJ-03-A-022 |
| Ai Di Cha/Ai Jiao Cha/Ai Cha Feng | *Ardisia japonica* (Thunb.) Blume | Myrsinaceae | Whole plant | Small shrub | Dried | Pulmonary bleeding, Sore throat, Trauma, Bronchitis, Menstrual disorders, Kidney ailments, Gastrointestinal diseases | Decoction, Powder for topical application | TJ-03-A-077 |
| Zong Shu Gen | *Trachycarpus fortunei* (Hook.) H.Wendl. | Arecaceae | Root | Tree | Dried | Threatened miscarriage | Decoction | TJ-03-A-076 |
| San Kuai Wa | *Oxalis griffithii* Edgew. & Hook.f. | Oxalidaceae | Whole plant or root | Herbaceous | Dried or fresh | Menstrual disorders, Ulcers, Diarrhea, Trauma, Rheumatic pain | Decoction, Fresh crushed for topical use | TJ-03-A-021 |
