# Supplementary material for: Medicinal plant use by the Tujia people in northeastern Guizhou, China: an ethnobotanical study
Source: Front Pharmacol. 2025 Mar 28;16:1522456. doi: 10.3389/fphar.2025.1522456 (PMC11985523; doi:10.3389/fphar.2025.1522456)
Supplement: Supplementary file 3 [file Table3.docx]

**Supplementary Table 3** **Application of other Ethnic and Modern Pharmacological Research on Traditional Herbal Medicine to the Tujia Ethnics in Tongren**

| Common Name | **Scientific Name** | Representative Chemical Components | Biological Activities and Medicinal Parts | Ethnic Groups Using the Herb | **References** |
| --- | --- | --- | --- | --- | --- |
| Cong Bai/Huo Cong | *Allium fistulosum* L. | Amino acids, Carbohydrates, Flavonoids | Roots have Anticancer, Antipyretic, Expectorant, Antimicrobial, Antiviral | Buyei, Dai, Dong, Daur, Ewenki, Gelao, Hani, Jino, Maonan, Mongolian, Qiang, Tujia, Wa, Yi, Tibetan | (Chen and Zhang, 2023) |
| Gan Shan Bian/Pan Long Qi | *Peliosanthes macrostegia* Hance | Flavonoids, Coumarins, Anthraquinones | Roots and Stems Anti-inflammatory, Antidepressant, Antitumor, Anti-aging, Anticoagulant | —— | (Feng et al., 2014) |
| Ma Gan Qi/Lu Yao | *Maianthemum japonicum* (A.Gray) LaFrankie | Steroids, Flavonoids, Proteins | Roots and Stems Antimicrobial, Antitumor, Antioxidant | Miao, Naxi, Pumi, Tujia | (Guan et al., 2024) |
| Ye Cong/Xiao Cong | *Allium macrostemon* Bunge | Proteins, Carbohydrates, Amino acids, Flavonoids | Roots and Leaves have Antioxidant, Antitumor | Korean, Hui, Mongolian, Naxi, Nu, Shui, Tujia, Yao, Tibetan | (Bing et al., 2007; Chen et al., 2016) |
| Bai Zao Xiu/Deng Tai Qi | *Paris lancifolia* Hayata/P*orella japonica var. calcicola* M. Hara | Steroids, Flavonoids, Terpenes, Essential oils | Roots and Stems have Antitumor, Antimicrobial, Anti-inflammatory, Sedative, Analgesic | Buyei, Korean, Dai, Dong, De'ang, Derung, Ewenki, Gelao, Jino, Jingpo, Lisu, Li, Maonan, Miao, Qiang, She, Shui, Tujia, Wa, Yao, Yi, Zhuang | (Dan et al., 2022) |
| Di Zhu/Tou Ding Zhu | *Trillium tschonoskii* Maxim. | Steroid saponins, Flavonoids, Fatty acids | Leaves and Stems have Anti-inflammatory, Analgesic, Antitumor, Anti-aging, Hepatoprotective | Tujia, Miao | (Chen et al., 2011) |
| Wan Nian Qing | *Rohdea japonica* (Thunb.) Roth | Saponins, Essential oils, Polysaccharides, Terpenes, Flavonoids | Whole grass have Antitumor, Analgesic, Anti-inflammatory, Antioxidant, Immunomodulatory | Bai, Buyei, De'ang, Dong, Jingpo, Lahu, Miao, Shui, Tujia | (Ma et al., 2023) |
| Huang Jing Can/Lao Hu Jiang | *Polygonatum* Mill. | Alkaloids, Polysaccharides, Steroid saponins | Roots and Stems have Antimicrobial, Anti-aging, Immune modulation | Tibetan, Tujia, Miao, Maonan, Gelao, Lisu, Bai, Dong, Naxi, Yao | (Chen, 1988) |
| Yi Wo Qu/Tian Dong | *Asparagus* *cochinchinensis* (Lour.) Merr. | Steroids, Polysaccharides, Amino acids | Roots have Antioxidant, Antitumor, Immunomodulation, Anti-inflammatory, Antimicrobial | Buyei, Korean, De'ang, Dong, Hani, Jino, Jingpo, Lahu, Li, Maonan, Miao, Tujia, Tibetan | (Lv et al., 2024) |
| Zhu Gen Qi | *Disporopsis fuscopicta* Hance | Steroid saponins, Flavonoids, Cardiac glycosides | Whole grass have Analgesic, Anti-inflammatory, Antitumor, Antimicrobial, Antioxidant | Buyei, Miao, Shui, Tujia, Yao | (Li et al., 2024) |
| Bai He | *Lilium brownii var. viridulum* Baker/*Cardiocrinum giganteum var. yunnanense* (Elwes) Stearn | Steroid saponins, Alkaloids, Polysaccharides, Phenolics | Whole grass have Antimicrobial, Antitumor, Antioxidant | Dong, Gelao, Lahu, Maonan, Miao, Naxi, Tujia, Yao, Mongolian, She, Wa, Yi, Zhuang | (Wu and Fan, 2024) |
| Ji Xiang Cao | *Reineckea carnea* (Andrews) Kunth | Steroid saponins, Flavonoids, Lignans, Terpenes | Whole grass have Hemolytic prevention, Cough suppressant, Expectorant, Anti-inflammatory, Analgesic, Blood sugar reduction | Bai, Buyei, Dong, Derung, Gelao, Hani, Lisu, Maonan, Miao, Naxi, Nu, Pumi, Shui, Tujia, Wa, Yao, Yi, Zhuang | (Wang and Chen, 2024) |
| Ma Ti Xiang/Zhi Zhu Xiang | *Valeriana jatamansi* Jones | Phenolics, Flavonoids, Lignans, Alkaloids | Roots and Stems have Antimicrobial, Antitumor, Anti-inflammatory, Antipyretic, Analgesic | Achang, Blang, Buyei, Dai, De'ang, Dong, Gelao, Hani, Jingpo, Lahu, Lisu, Maonan, Miao, Yi, Naxi, Nu, Shui, Tujia, Uighur, Wa, Yao, Zhuang | (Wang et al., 2018) |
| Ban Bian Lian | *Lobelia chinensis* Lour. | Alkaloids, Flavonoids, Terpenes, Coumarins | Whole grass have Antitumor, Anti-inflammatory, Analgesic, Antimyocardial ischemia‒reperfusion | Tujia, Achang, De'ang, Dong, Gelao, Jingpo, Li, Miao, She, Yao, Zhuang, Maonan | (Wang, 2020) |
| Si Kuai Wa/Si Da Tian Wang | *Lysimachia paridiformis* Franch. | Triterpene saponins, Steroid saponins | Whole grass have Antibacterial, Antifungal, Antiviral, Hepatoprotective, Antioxidant | Tujia, De'ang, Jingpo, Miao | (Liu et al., 2015) |
| Man Tian Xing | *Lysimachia congestiflora* Hemsl. | Flavonoids, Triterpene saponins, Steroids | Whole grass have Activating blood circulation, Reducing swelling, Pain relief, Expelling cold, Detoxifying | Tujia, Achang, De'ang, Hani, Jingpo | (Chen et al., 2016) |
| Zhu Er Duo/Che Qian Cao | *Plantago asiatica* L. | Flavonoids, Terpenes | Whole grass have Anti-inflammatory, Antibacterial, Anti-ulcerative, Antioxidant | Tujia, Bai, Buyei, Blang, Dong, Zhuang, Yi | (Peng et al., 2023) |
| Yi Mu Cao | *Leonurus japonicus* Houtt. | Alkaloids, Flavonoids, Diterpenes, Phenylethanol glycosides | Whole grass have Anti-atherosclerosis, Antioxidant, Anti-apoptotic | Bai, Buyei, Korean, Dai, Dong, Derung, Gelao, Hani, Lahu, Lisu, Maonan, Mongolian, Miao, Naxi, Qiang, She, Tujia, Yi, Tibetan, Zhuang | (Xiong et al., 2024) |
| Shan Bo He/Chou Bo He | *Mentha canadensis* L. | Essential oils, Flavonoids, Terpenes, Phenolic acids | Leaves and Stems have Antibacterial, Antiviral, Anti-inflammatory, Antioxidant, Antitumor, Anti-fertility | Buyei, Korean, Dai, Dong, Hani, Kazakh, Jing, Lisu, Maonan, Mongolian, Miao, Naxi, She, Tujia, Wa, Uighur, Yao, Yi, Yugur, Tibetan, Zhuang, Taiwanese minorities | (Huang et al., 2007) |
| Deng Long Cao/Feng Lun Cao | *Trifolium polycephalum* Ser. | Terpenes, Flavonoids, Saponins | Roots have Common cold, Anti-inflammatory, Hepatoprotective | Dai, Hani, Miao, She, Shui, Tujia, Yao | (Miao et al., 2024) |
| Mao Yan Cao/Dao Du San | *Euphorbia helioscopia var. ceretana* Sennen | Terpenes, Flavonoids, Phenylpropanoids, Sterols | Leaves and Stems have Antitumor, Antioxidant, Antibacterial | Korean, Miao, Tujia, Tibetan | (Cui et al., 2024) |
| Suan Pan Zi | *Glochidion puberum* (L.) Hutch. | Terpenes, Flavonoids, Coumarins, Steroid compounds | Fruit、Roots and Leaves have Antitumor, Analgesic | Achang, Buyei, De'ang, Dong, Gelao, Hani, Jingpo, Li, Maonan, Miao, She, Shui, Tujia, Yao, Zhuang | (Tan et al., 2023) |
| Han Xiu Cao | *Mimosa pudica f. glabrior* Benth. | Flavonoids, Phenolics, Organic acids | Whole grass and Roots have Anti-aging, Anticancer, Cardiovascular disease prevention, Anti-inflammatory, Analgesic | Achang, Dai, De'ang, Hani, Jingpo, Lisu, Maonan, Naxi, Tujia, Wa, Zhuang | (Li et al., 2023) |
| Yun Shi | *Caesalpinia vesicaria* Lam. | Flavonoids, Terpenes, Phenolics | Roots and Stems have Anti-inflammatory, Antioxidant, Antimalarial | Tujia, Achang, Buyei, Dai, De'ang, Dong, Gelao, Hui, Jingpo, Maonan, Miao, Wa, Yao, Yi, Zhuang | (Xie and Wang, 2024) |
| Huang Ge Teng | *Pueraria montana var. lobata* (Willd.) Maesen & S.M.Almeida ex Sanjappa & Predeep | Puerarin, Soyasaponins | Roots and Stems have Anti-osteoporotic | Achang, Buyei, Korean, De'ang, Dong, Jino, Lisu, Mongolian, Miao, Naxi, Nu, She, Shui, Tujia, Yao, Yi | (Tang et al., 2022) |
| Ye Wan Dou | *Lotus corniculatus* L. | Terpenes, Flavonoids, Amino acids | Whole grass have Antioxidant, Antimicrobial, Anticancer, Diabetes management, Hepatoprotective | Tujia | (Xu et al., 2015) |
| Xue Teng | *Sargentodoxa cuneata* (Oliv.) Rehder & E.H.Wilson | Phenolic acids, Lignans, Triterpenes, Essential oils | Roots and Stems have Anti-inflammatory, Antiviral, Antiallergic, Antioxidant | Buyei, Dong, Miao, Yao, Shui, Zhuang, Yi, Tujia | (Yin et al., 2024) |
| Bai Yao/Qian Jin Teng | *Stephania herbacea* Gagnep. | Alkaloids, Lignans, Sterols, Flavonoids, Phenolics | Roots and Stems have Antitumor, Antifibrotic, Antibacterial, Anti-inflammatory | She, Tujia, Taiwanese minorities | (Zheng and Wang, 2023) |
| Di Ku Dan | *Tinospora sagittata* (Oliv.) Gagnep. | Palmatine | Roots have Anti-inflammatory, Antiswelling, Analgesic | Achang, Dai, De'ang, Dong, Jingpo, Maonan, Miao, Mulam, Naxi, Shui, Wa, Yao, Tibetan, Zhuang | (Liu, 2020) |
| Shan Wu Gui | *Stephania epigaea* H.S.Lo | Alkaloids | Roots have Anti-inflammatory, Analgesic | Bai, Dai, Jino, Lahu, Miao, Wa, Yi, Tibetan | (Chi et al., 2008) |
| Hai Jin Sha | *Lygodium japonicum* (Thunb.) Sw. | Flavonoids, Phenolic acids and their glycosides, Triterpenes | Dried fruits have Cholagogic, Antilithic, Antioxidant, Antibacterial | Achang, Bai, Buyei, Korean, Dai, De'ang, Dong, Gelao, Hani, Jino, Jingpo, Lahu, Lisu, Maonan, Mongolian, Miao, Mulam, Nu, She, Tujia, Yao, Yi, Tibetan, Zhuang, Taiwanese minorities | (Ni et al., 2020) |
| Xiang Ya Cao | *Festuca brauniana* (Nees) Walp. | Polyphenols, Pentacyclic triterpenes, Essential oils | Antioxidant, Antibacterial | Achang, Dai, De'ang, Hani, Jingpo, Miao, Naxi, Yi, Zhuang | (Wang et al., 2022) |
| Yan Mai | *Avena fatua subsp. nuda* (L.) Thell. | Proteins, Amino acids, Unsaturated fatty acids, Saponins | Whole grass have Lipid-lowering, Vasoconstrictive, Antioxidant | Mongolian, Yi, Tibetan | (Wang et al., 2024) |
| Ma You | *Sesamum indicum* L. | Fatty acids, Trace elements, Vitamins | Mature seeds have Blood lipid regulation, Cholesterol reduction, Antioxidant, Antitumor | Achang, Korean, Dai, De'ang, Dong, Tujia, Uighur, Yao, Yi, Hani, Mongolian, Jingpo, Lahu | (Zhi et al., 2023) |
| Ku Gua | *Momordica charantia var. abbreviata* Ser. | Alkaloids, Saponins, Peptides, Vitamins, Minerals | Dried fruits have Glycemic control, Lipid-lowering, Antioxidant, Anticancer | Dai, Dong, Jino, She, Tujia, Wa, Yi, Zhuang | (Zhang et al., 2024) |
| Bai Wei Lian/Jin Gui Lian | *Schizocarpum guatemalense* Cogn. ex Donn.Sm./*Hemsleya chinensis var. longivillosa* (C.Y.Wu & Z.L.Chen) D.Z.Li | Triterpene saponins | Roots and Stems have Antitumor, Antioxidant, Anti-inflammatory | Buyei, Dong, Gelao, Maonan, Miao, Tujia | (Tong et al., 2023) |
| Ye Xi Gua/Dong Gu Zi | *Trichosanthes kirilowii* Maxim. | Triterpenes, Flavonoids, Fatty acids, Plant sterols | Dried fruits have Antioxidant, Anti-inflammatory, Antimicrobial, Antithrombotic | Korean, Dong, Mongolian, Miao, Naxi, She, Tujia, Uighur, Yi, Zhuang | (Yang et al., 2024) |
| Pa Di Hong Mao | *Chrysosplenium pilosum* Maxim. | Flavonoids, Pentacyclic triterpenes, Essential oils | Whole grass and Roots have Antitumor, Antiviral, Cytotoxic | Tibetan | (Mu et al., 2019) |
| Yan Bai Cai | *Bergenia purpurascens* (Hook.f. & Thomson) Engl. | Polyphenols, Flavonoids | Leaves and Stems have Anti-inflammatory, Antitumor, Antioxidant, Anti-arrhythmic | Bai, Dai, Dong, Lisu, Mongolian, Miao, Naxi, Pumi, Wa, Yao, Yi, Tibetan, Zhuang | (Pan et al., 1998) |
| Sheng Jiang | *Zingiber officinale f. rubens* (Makino) M.Hiroe | Essential oils, Gingerols | Roots and Stems have Anti-ulcerative, Antibacterial, Anti-inflammatory, Antioxidant | Achang, Buyei, Korean, Dai, Dong, Dongxiang, Gelao, Hani, Jino, Lisu, Mongolian, Miao, Naxi, Nu, She, Tujia, Wa, Uighur, Yao, Yi, Tibetan, Zhuang, Taiwanese minorities | (Hu and Dang, 2024) |
| Shan Jiang | *Alpinia japonica* (Thunb.) Miq. | Essential oils, Flavonoids, Steroids, Terpenes | Roots and Stems have Antioxidant, Blood pressure reduction, Antispasmodic, Analgesic | Dai, De'ang, Dong, Miao, She, Tujia, Yao, Zhuang | (Jiang et al., 2024) |
| Jin Si Cao | *Polytrichum formosum* Hedw. | Ketones, Flavonoids | Whole grass have Antitumor, Cytotoxic, Anti-inflammatory | She, Yao | (Yuan et al., 2018) |
| Tian Ji Huang/Di Er Cao | *Hypericum japonicum* Thunb. | Flavonoids, Koumine, Phenolic compounds | Leaves、flowers and Stems have Liver protection, Jaundice treatment, Antimicrobial, Antiviral | Buyei, Dai, Dong, Gelao, Hani, Jino, Lahu, Lisu, Maonan, Miao, She, Tujia, Wa, Yao, Yi, Zhuang | (Zhou et al., 2024) |
| Xiao Niu Xi/Dui Ye Qi | *Chloranthus serratus* (Thunb.) Roem. & Schult. | Saponins, Steroids, Polysaccharides | Roots have Antioxidant, Anti-inflammatory | Dong, Lahu, Miao, Tujia, Yao | (Liu et al., 2024) |
| Si Ye Xi Xin/Si Kuai Wa | *Chloranthus serratus* (Thunb.) Roem. & Schult. | Bicyclic terpenes, Flavonoids | Roots have Antioxidant, Anti-inflammatory | Buyei, Hani, Miao, Tujia, Dong | (Zhang et al., 2023) |
| Si Kuai Wa | *Chloranthus spicatus* (Thunb.) Makino/*Chloranthus henryi* Hemsl. | Terpenes | Whole grass have Anti-inflammatory, Antitumor, Antifungal, Antiviral, Neuroprotective | Buyei, Dong, Gelao, Hani, Maonan, Miao, Tujia, Yao, Bai, Dai, Yi, Zhuang, Wa, Lahu, Jino | (Li et al., 2023) |
| Zi Hua Di Ding | *Viola diamantiaca* Nakai/*Viola betonicifolia var. cordifolia* Hara | Coumarins, Flavonoids, Glycosides | Whole grass have Antibacterial, Anti-inflammatory, Antioxidant, Antiviral, Immunomodulatory, Anticancer | Buyei, Korean, Dong, Gelao, Maonan, Miao, Pumi, Mongolian, She, Hani, Lisu, Naxi, Tujia, Yao, Wa, Taiwanese minorities | (Qiu et al., 2024) |
| Luan Zi Cao/Yan Song | *Sedum elatinoides* Franch./S*edum lineare* Thunb. | Flavonoids, Sterols, Pentacyclic triterpenes, Phenylpropanoids | Leaves and Stems have Antitumor, Anti-inflammatory, Analgesic, Antioxidant | Miao, She, Tujia, Yao, Yi, Zhuang | (Chen and Yu, 2023) |
| La Jiao Cao | *Sedum oligospermum* Maire | Alkaloids, Flavonoids, Active polysaccharides | Whole grass have Anti-inflammatory, Antioxidant, Neuroprotective | Dong, Tujia | (Huang et al., 2017) |
| Wa Song/Wa Lian Hua | *Cotyledon elizae* A.Berger ex Raym.-Hamet | Flavonoids, Organic acids, Triterpenes, Cardiac glycosides | The dry part of the ground have Anti-inflammatory, Analgesic, Antibacterial, Cardiac support | Achang, De'ang, Evenki, Jingpo, Mongolian, Tujia, Yao, Tibetan | (Fan et al., 2020) |
| Ba Bao Cao/Huo Yan Cao | *Hylotelephium erythrostictum* (Miq.) H.Ohba | Flavonoids, Glycosides, Organic acids | Leaves and Stems have Antibacterial, Anti-inflammatory, Antioxidant, Antitumor | Tujia | (Chen and You, 2010) |
| Da Bu Si/Luo Di Sheng Gen | *Bryophyllum pinnatum* (Lam.) Oken | Flavonoids, Alkaloids, Steroids, Terpenes | Roots or Whole grass have Anti-inflammatory, Analgesic, Immunosuppressive, Immunomodulatory, Antitumor | Bai, Dai, De'ang, Hani, Jino, Jingpo, Lahu, Lisu, Maonan, Wa, Taiwanese minorities | (Duan et al., 2017) |
| Dou Ban Cai | *Sedum majus* (Hemsl.) Migo | Flavonoids, Alkaloids, Steroids, Terpenes | Leaves and Stems have Anti-ulcer, Antibacterial, Antioxidant, Neuroprotective | Tujia | (Li et al., 2022) |
| Da Bu Si | *Sedum chauveaudii* Raym.-Hamet | Flavonoids, Alkaloids | Anti-ulcer, Antibacterial, Antioxidant | —— | (Qi and Niu, 2011) |
| Fei Cai | *Sedum oligospermum* Maire | Flavonoids, Unsaturated fatty acids, Glycosides, Sterols | Antioxidant, Antibacterial | Korean | (Zhang et al., 2024) |
| Tu San Qi | *Phedimus aizoon* (L.) 't Hart | Glycosides, Coumarins, Organic acids, Flavonoids | Roots have Antioxidant, Antibacterial, Anticancer | Korean, Dong, Evenki, Manchu, Miao, Pumi, Qiang, Tujia, Tibetan | (Tang et al., 2024) |
| Tu Dang Shen | *Campanumoea javanica* Blume | Phenylpropanoids, Sugars and glycosides, Acetylenes, Flavonoids and flavonoid glycosides | Roots have Antitumor, Immunomodulatory, Antioxidant | Yi, Miao, Hani, Tujia, Lahu | (Lu et al., 2023) |
| Ye Dang Shen | *Codonopsis javanica subsp. japonica* (Maxim. ex Makino) Lammers | Alkene glycosides, Organic acids, Glycosides, Adenosine | Roots have Antioxidant, Immunomodulatory, Anti-aging | Dai, De'ang, Dong, Hani, Jino, Lahu, Lisu, Maonan, Miao, Naxi, Shui, Tujia, Yao, Yi, Tibetan, Zhuang | (Zhan et al., 2024) |
| Ai Cao | *Artemisia lavandulifolia* Salisb./*Artemisia indica* Willd./*Artemisia argyi* H.Lév. & Vaniot | Plant fibers, Polysaccharides, Flavonoids, Essential oils | Whole grass have Antibacterial, Antiviral, Hemostatic, Immunomodulatory | Dai, De'ang, Dong, Hani, Jino, Lahu, Lisu, Maonan, Miao, Naxi, Shui, Tujia, Yao, Yi, Tibetan, Zhuang | (Li et al., 2024) |
| Jiu Li Guang | *Senecio scandens* Buch.-Ham. ex D.Don | Alkaloids, Flavonoids, Essential oils, Terpenes | Roots have Antibacterial, Anti-inflammatory, Antiviral, Antitumor, Antioxidant | Bai, Buyei, De'ang, Dong, Derung, Gelao, Hani, Jingpo, Lahu, Lisu, Maonan, Miao, Naxi, Nu, Qiang, She, Tujia, Wa, Yao, Yi, Tibetan, Zhuang, Taiwanese minorities | (Chen and Xie, 2015) |
| Cang Er Zi/Zhan E Zi/Cang Er Zi | *Xanthium strumarium* L. | Glycosides, Phenolic acids and derivatives, Alkaloids, Flavonoids | Dried fruits have Antimicrobial, Antitumor, Anti-inflammatory, Analgesic, Antioxidant, Antiallergic | Achang, Bai, Korean, Dai, Dongxiang, Evenki, Gelao, Hani, Kazakh, Lisu, Maonan, Mongolian, Miao, Mulam, Naxi, She, Tujia, Uighur, Yao, Yi, Tibetan, Zhuang, Taiwanese minorities | (Dong et al., 2024) |
| Shan Luo Bo/Ye Luo Bo | *Parasenecio forrestii* W.W.Sm. & J.Small | Terpenes and derivatives, Brassins, Organic acids, Alkaloids | Leaves、flowers and Stems have Antibacterial, Throat discomfort relief, Antioxidant, Blood pressure reduction | Tibetan, Yi | (Wang et al., 2016) |
| Hong Chai Hu/Yi Zhi Huang Hua | *Solidago decurrens* Lour. | Terpenes, Flavonoids, Phenylpropanoids | Roots have Antitumor, Antioxidant, Blood sugar reduction, Antimicrobial | Buyei, De'ang, Dong, Gelao, Kazakh, Jingpo, Maonan, Miao, She, Tujia, Yao, Zhuang | (He et al., 2024) |
| Da Huo Cao/Qing Ming Cao | *Psychrophyton buchanani* (Kirk) Anderb. | Flavonoids, Diterpenes | Roots have Anti-inflammatory, Antioxidant, Antibacterial | Bai, Dong, Derung, Hani, Lisu, Miao, Naxi, Shui, Tujia, Wa, Yi, Tibetan | (Gao et al., 2019) |
| Ye Yan | *Carpesium abrotanoides* L. | Alkaloids, Glycosides, Phenolics | Roots have Anti-inflammatory, Antibacterial | Bai, Lisu, Miao, Tujia, Yi, Tibetan | / |
| Dong Feng Cai | *Aster scaber* Thunb. | Flavonoids, Phenolic acids | Whole grass have Antiviral, Antibacterial, Antitumor, Anti-inflammatory, Antioxidant | Tujia | (Xie et al., 2023) |
| San Qi | *Gynura japonica* (Thunb.) Juel | Phenolic acids, Flavonoids, Glycosides | Roots have Antibacterial, Anti-inflammatory, Anti-HIV | Achang, Bai, Buyei, Dong, Hani, Lahu, Miao, Naxi, Pumi, Qiang, She, Tujia, Yao, Yi, Zhuang | (Liu et al., 2024) |
| Shen Jiao Cao/Cui Yun Cao | *Selaginella uncinata* (Desv.) Spring | Flavonoids, Phenolic acids, Anthraquinones, Terpenes | Roots have Anti-inflammatory, Antioxidant, Immunomodulatory | Hani, Jino, Li, Miao, She, Tujia, Yao, Yi, Zhuang | (Zhang et al., 2023) |
| Shi Shang Bai/Yan Juan Bai | *Selaginella involvens* (Sw.) Spring | Alkaloids, Saponins, Sterols | Whole grass have Anti-inflammatory, Antitumor, Antioxidant | Hani, Lisu, Miao, Nu, She, Tujia | (Chen et al., 2023) |
| Jiu Tou Shi Zi | *Peristrophe japonica* (Thunb.) Bremek. | Alkanes, Sterols | Roots and Stems have Anti-inflammatory, Antitumor, Lipid regulation | Buyei, Dong, Miao, Shui, Tujia, Yao, Zhuang, Taiwanese minorities | (Gao et al., 2020) |
| Xiao Qing Cao/Gan Ji Cao | *Justicia procumbens* Blume | Lignans, Flavonoids, Steroids | Antioxidant, Anti-aging, Anti-inflammatory | Dong, Hani, Lisu, Miao, She, Tujia, Yao, Zhuang | / |
| Yan Pi Pa/Yan Ju Hua | *Briggsia latisepala* Chun ex K.Y.Pan | Flavonoids, Sesquiterpenes | Roots and Stems have Antibacterial, Antiviral |  | (Pan et al., 1998) |
| Shi Diao Lan | *Lysionotus pauciflorus* Maxim. | Flavonoids, Essential oils, Phenylethanol glycosides | Whole grass have Antibacterial, Anti-inflammatory, Hepatoprotective, Antitussive | Buyei, Dai, Dong, Gelao, Maonan, Miao, Qiang, She, Shui, Tujia, Yao, Zhuang | (Zhou et al., 2021) |
| Jia Tian Ma | *Corallorhiza trifida var. trifida* | Phenolic compounds and glycosides | Dried Tubers hace Antiepileptic, Anticonvulsant, Sleep improvement |  | (Liang et al., 2024) |
| Shan Ci Gu/Ye Bai Ji/Bai Mao Gu | *Cremastra appendiculata* (D.Don) Makino | Mannose-bound glucans | Dried phosphorus stem have Antitumor, Sedative, Hypnotic, Antitussive | Korean, Dong, Miao, Tujia | (Zhang et al., 2023) |
| Huo Xue Zhu/Mao Suan Gu | *Pleione bulbocodioides* (Franch.) Rolfe | Phenylpropanoids, Lignan derivatives, Glycosides | Stems have Antitumor, Antioxidant, Anti-inflammatory | Korean, Mongolian, Miao, Tujia | (Mu et al., 2008) |
| Xiang Long Cao | *Pogonia japonica* Rchb.f. | Triterpenes, Anthraquinones | Whole grass have Antibacterial, Anti-inflammatory | Buyei | (Qiu et al., 2014) |
| Fei Xing Cao | *Goodyera schlechtendaliana f. similis* (Blume) Makino | Xanthones, Cycloether terpenes, Flavonoids | Whole grass have Anticancer, Immunomodulatory, Antimicrobial | Tujia | (Liu et al., 2019) |
| Shuang Shen Cao | *Habenaria davidii* Franch. | Terpenoids, Polysaccharides, Alkanes | Roots have Antibacterial, Anti-inflammatory, Antitumor, Antioxidant, Anti-aging, Immunomodulatory | Tujia, Bai | (Tan et al., 2024) |
| Shuang Shen Can | *Habenaria dentata* (Sw.) Schltr. | Triterpene glycosides, Saponins | Roots and Stems have Anti-inflammatory, Immunomodulatory | Dai, Dong, Hani, Lahu, Lisu, Miao, Tujia, Yi, Taiwanese minorities | (Ma et al., 2020) |
| Yan Shi Hu | *Bulbophyllum nutans* (Thouars) Thouars | Polysaccharides, Amino acids, Polyphenols | Roots have Anti-inflammatory, Antioxidant, Immunomodulatory | Miao, Tujia | (Chen, 2016) |
| Lian Huan Cao | *Calanthe discolor var. discolor* | Monoterpenes, Cycloether terpenes, Flavonoids | Dried phosphorus stem have Anti-inflammatory, Antitumor, Antioxidant, Antibacterial | Dong, Tujia, Miao | (Wan et al., 2010) |
| Hong Hua Liao | *Polygonum runcinatum* Buch.-Ham. | Flavonoids, Sterols | Flowers have Antioxidant, Anti-inflammatory, Antibacterial | Bai, Dong, Tujia, Yao, Yi | (Xie et al., 2024) |
| Zhu Sha Qi | *Pleuropterus ciliinervis* Nakai | Anthraquinones, Emodin | Roots have Antitumor, Anti-aging, Anti-inflammatory, Antibacterial, Antiviral | Miao, Tujia, Qiang, Yi | (Wang et al., 2022) |
| Niu Dai Huang/Jin Da Huan | *Rumex crispus subsp. littoreus* (Hardy) Akeroyd | Bile acids, Sterols, Bilirubin | Roots and Stems have Anti-inflammatory, Antibacterial | Korean, Dong, Evenki, Kazakh, Mongolian, She, Tujia, Uighur, Tibetan | (Guo et al., 2024) |
| She Dao Tui | *Polygonum perfoliatum* L. | Glycosides, Phenolics | Stems have Anti-inflammatory, Antioxidant, Antibacterial, Antiviral | Buyei, Korean, Dai, Dong, Gelao, Hani, Miao, Tibetan, Zhuang, Tujia, Yi | (Yao et al., 2023) |
| Hou Er Qi | *Bistorta vivipara* (L.) Gray | Saponins, β-sitosterol | Whole grass havAnti-inflammatory | Korean, Kazakh, Lisu, Naxi, Qiang, Yi, Tibetan | (Liu et al., 2023) |
| Xue Li Mei/Qing Yu Dan | *Gentiana rhodantha* Franch. | Bile acids | Roots and Stems have Immune enhancement, Anti-fatigue, Sleep promotion, Blood pressure reduction | Bai, Buyei, Dong, Gelao, Hani, Lisu, Maonan, Miao, Naxi, Nu, Tujia, Yao, Tibetan | (Yang et al., 2024) |
| Xiao Long Dan/Tu Long Dan | *Halenia corniculata* (L.) Cornaz | Bitter glycosides, Sterols, Esters, Alkanes | Roots and Stems have Anti-inflammatory, Analgesic, Antitumor, Antioxidant | Tujia, Lisu, Mongolian, Naxi, Pumi, Qiang, Tibetan | (Cao et al., 2024) |
| Yan Long Dan | *Gentiana squarrosa* Ledeb. | Cycloether terpenes, Triterpenes, Xanthones, Flavonoids | Roots and Stems have Anti-inflammatory, Analgesic, Hepatoprotective, Antitumor | Mongolian, Tibetan | (Jiao et al., 2024) |
| Dui Yue Cao/Zhu Ye Qi | *Vincetoxicum pycnostelma* Kitag. | Hypericin | Leaves have Anti-inflammatory, Sedative, Antioxidant, Antibacterial, Antispasmodic | Buyei, Korean, Dong, Gelao, Hani, Maonan, Mongolian, Miao, She, Tujia, Yao, Yi, Zhuang | (Wu et al., 2024) |
| Hei Gu Teng/Hei Hu Teng | ­­—— | Flavonoids, Alkaloids, Polysaccharides, Organic acids | Whole grass havAnti-inflammatory, Wound healing promotion, Antitumor, Cardiotonic | —— | (Shu et al., 2023) |
| Nai Jiang Teng | *Cynanchum rostellatum* (Turcz.) Liede & Khanum | Bitter steroid glycosides, Flavonoids, Polysaccharides, Oils | Whole grass have Anti-inflammatory, Antioxidant | Mongolian | (Shen, 2014) |
| Da Feng Cao | *Ranunculus bungei* Steud. | Magnesium lactate, Succinic acid, Anisic acid, Vanillic acid | Whole grass have Antitumor, Anti-inflammatory, Analgesic, Antibacterial | Achang, Bai, Blang, Buyei, Dai, De'ang, Dong, Hani, Jino, Jingpo, Jing, Lahu, Lisu, Maonan, Miao, Naxi, She, Tujia, Wa, Yao, Yi, Zhuang | (Jiao et al., 2024) |
| Tu Ren Shen | *Talinum paniculatum* (Jacq.) Gaertn. | Anthraquinones, Sugars, Amino acids, Organic acids | Roots have Anti-inflammatory, Antibacterial, Antioxidant, Sun protection | Bai, Dong, Lisu, Maonan, Miao, Mulam, She, Tujia, Yao, Yi, Zhuang | (Chang et al., 2023) |
| Si Liang Ma | *Asarum sieboldii* Miq. | Lignans, Essential oils, Acids | Whole grass with roots Anti-inflammatory, Analgesic, Antitussive, Antimicrobial, Antiviral | Korean, Hui, Mongolian, Miao, Naxi, Tujia, Uighur | (Tan et al., 2024) |
| Ma Ti Xiang/Pen Cao | *Saruma henryi* Oliv. | Aristolochic acid amides, Aristolochic acids | Dried fruits have Antitumor, Antibacterial, Antiviral | Tujia, She | (Zhang et al., 2020) |
| Ma Ti Xiang/Ma Xi Xin | *Asarum macranthum* Hook.f. | Essential oils | Dried fruits have Fever reduction, Anti-inflammatory, Analgesic, Sedative | Dong, Tujia, Yao | (Wang et al., 2019) |
| She Shen | *Aristolochia tubiflora* Dunn | Alkaloids | Roots have Antitumor, Antibacterial, Blood pressure reduction, Anti-fertility | Miao, Tujia, Yao | (Qiao et al., 2006) |
| Ma Sang | *Coriaria nepalensis* Wall. | Flavonoids, Terpenes | Roots and leaves have Antibacterial | Buyei, Dong, Lisu, Miao, Nu, Qiang, Shui, Tujia, Yao, Yi, Zhuang | (Chen et al., 2022) |
| Po Xue Zi/Lao Guan Cao | *Geranium wilfordii* Maxim. | Flavonoids, Catechins, Tannins | Whole grass have Antiviral, Antibacterial, Anti-inflammatory | Mongolian, Miao, Tujia, Yi, Zhuang, Tibetan | (Kong et al., 2023) |
| Niu Jiao Qi/Tie Po Luo/Guai Zi Qi | *Beesia calthifolia* Ulbr. | Terpenes, Glycosides | Roots and Stems have Antitumor, Anti-inflammatory, Antiviral | Bai, Lisu, Naxi, Tujia | (Yang et al., 1995) |
| Sui Gu Cao/Ye Wu Tou | *Aconitum sinomontanum* Nakai | Diterpene alkaloids, Guaiane sesquiterpenes | Roots have Analgesic, Local anesthetic, Anti-arrhythmic | Miao, Tujia | (Chen et al., 2024) |
| Lao Hu Xiang/Shi Huang Cao | T*halictrum aquilegiifolium var. sibiricum* Regel & Tiling/*Thalictrum microgynum* Lecoyer ex Oliv. | Alkaloids, Flavonoids, Triterpenes | Whole grass have Antitumor, Antiparasitic, Antiviral | Korean, Lisu, Mongolian, Tibetan | (Lv et al., 2024) |
| Tian Qu Zi/Qian Nian Lao Shu Shi | *Semiaquilegia adoxoides* Makino | Alkaloids, Lactones, Cyanogenic nitro compounds, Phenolics | Roots have Antibacterial, Anti-inflammatory activity, Cytotoxic activity, Antioxidant activity | Dong, Gelao, Maonan, Miao, She, Tujia, Yao, | (Wu et al., 2023) |
| Ye Mian Hua/Da Po Wan Wan Hua | *Anemone vitifolia* Buch.-Ham. ex DC. | Saponins, Flavonoids, Coumarins | Leaves and Stems have Antitumor, Anti-inflammatory, Antibacterial, Analgesic, Sedative | Buyei, Dong, Hani, Maonan, Miao, She, Tujia, Yao, Yi, Zhuang | (Li et al., 2017) |
| Leng Fan Tuan/Xiang Xue Teng | *Kadsura heteroclita* (Roxb.) Craib | Saponins, Tannins, Flavonoids | Roots and Stems have Hepatoprotective, Antitumor, Neuroprotective, Cardioprotective | Dai, Hani, Jino, Li, Maonan, Miao, Mulam, Tujia, Wa, Yao, Zhuang | (Xiong et al., 2004) |
| Da Hui | *Illicium henryi* Diels | Python toxins | Dried fruits have Analgesic, Platelet aggregation inhibition | Tujia | (Li and Dai, 2023) |
| Leng Fan Tuan/Xiao Xiang Teng | *Schisandra* Michx. | Lignans, Terpenes, Steroids | Whole grass have Anti-fibrotic, Antioxidant, Antiplatelet | Bai, Dong, Hani, Lisu, Miao, Tujia, Yi | (Lia et al., 1999) |
| Ba Yue Zha/Ba Yue Gua | *Akebia quinata* (Thunb. ex Houtt.) Decne./*Akebia trifoliata* (Thunb.) Koidz. | Alkaloids, Flavonoids, Polysaccharides | Friuts have Immunomodulatory, Antitumor | Korean, Dong, Mongolian, Miao, Tujia, Yao, Yi | (Zhang et al., 2024) |
| Yi Zhi Jian | *Ophioglossum vulgatum var. reticulatum* (L.) D.C.Eaton | Flavonoids, Essential oils, Steroids | Whole grass with roots have Hepatoprotective, Detoxifying, Anti-ulcer | Dai, Dong, Hani, Lahu, Miao, Naxi, Qiang, Tujia, Yao, Yi, Tibetan, Zhuang | (Zeng et al., 2021) |
| Jian Zhong Xiao/Mu Zhu Teng | *Ampelopsis delavayana* Planch. ex Franch./*Ampelopsis delavayana var. setulosa* (Diels & Gilg) C.L.Li | Terpenes, Tannins | Leavea and Stems have Anti-inflammatory, Antiviral, Hepatoprotective | Buyei, Dai, Dong, Hani, Lahu, Miao, Tujia, Wa, Yao, Yi | (Meng, 1994) |
| Shui Chang Shan/Yi Zhu Xiang | *Anotis urophylla* (Wall. ex Wight & Arn.) Hook.f. | Mannitol, Flavonoid glycosides | Whole grass have Antitussive | / | (Tu et al., 2021) |
| Ci Li | *Rosa roxburghii* Tratt. | Pentacyclic triterpenes, Flavonoids | Friuts have Anti-inflammatory, Antibacterial, Antitumor, Metabolic regulation | Buyei, Dong, Gelao, Maonan, Miao, Shui, Tujia, Yi | (Yang et al., 2024) |
| Lu Bian Huang/Ma Bian Cao | *Geum aleppicum* auct. | Triterpenes and their glycosides, Tannins | Dry ground have Immune enhancement, Blood nourishment, Vision protection | Bai, Buyei, Dong, Oroqen, Kazakh, Miao, Tujia, Tibetan, Zhuang, Yi, Yao, Shui, She, Naxi, Korean, Lisu | (Liu et al., 2021) |
| Di Feng Zi | *Potentilla freyniana var. sinica* Migo | Terpenes | Whole grass have Antibacterial, Anti-inflammatory, Antitumor | Buyei, Dong, Miao, Shui, Tujia, Yao, Zhuang | (Chen et al., 2015) |
| Hong Zi | *Pyracantha pyracantha* (L.) Voss | Benzamide alkaloids | Roots have Antibacterial, Antioxidant, Antitumor | Bai, Dong, Miao, Tujia, Yi | (Chen, 2016) |
| Shi Jie Mei | *Rosa saturata* Baker | Cyanogenic glycosides, Flavonoids, Triterpenes | Whole grass have Antitumor, Antioxidant, Antitussive | Bai, Dong, Miao, Tujia, Yi | (Guo et al., 2023) |
| Wu Pao | *Rubus setchuenensis* Bureau & Franch. | Terpenes, Flavonoids, Phenolic acids | Whole grass have Antibacterial, Anti-inflammatory, Antitumor | Miao, Tujia | (Xu, 2006) |
| Zai Yang Pao | *Rubus corchorifolius* L.f. | Flavonoids, Tannins, Coumarins | Roots and Leaves have Anti-diarrheal, Anti-inflammatory, Antibacterial | Dong, Oroqen, Miao, She, Tujia, Tibetan | (Wang et al., 2014) |
| Qiu Hai Tang | *Begonia grandis* Dryand. | Cardiac glycosides, Flavonoids, Saponins | Friuts and stems have Anticoagulant, Anti-inflammatory, Antibacterial | Bai, De'ang, Dong, Jingpo, Lahu, Lisu, Tujia, Yao, Zhuang | (Fan et al., 2011) |
| Jin Yin Hua | *Urtica japonica* Thunb. | Organic acids, Essential oils, Flavonoids | Whole grass have Antimicrobial, Anti-inflammatory, Antipyretic | Achang, Bai, Buyei, Korean, Dai, De'ang, Dong, Gelao, Hani, Jingpo, Lisu, Jing, Mongolian, Miao, Mulam, Naxi, Nu, She, Tujia, Wa, Yao, Zhuang | (Luo et al., 2024) |
| Jie Gu Cao | *Sambucus javanica* Reinw. ex Blume | Flavonoids, Phenolics, Tannins | Whole grass have Dispels wind-dampness, Promotes blood circulation, Hemostatic | Buyei, Dai, Dong, Lahu, Lisu, Maonan, Miao, Mulam, She, Tujia, Yao, Yi, Tibetan, Zhuang, Taiwanese minorities | (Wang et al., 2023) |
| Zhe Er Gen/Yu Xing Cao | *Houttuynia cordata* Thunb. | Essential oils and Flavonoids | Whole grass have Antibacterial, Antipyretic, Anti-inflammatory, Antitumor | Achang, Bai, Blang, Buyei, Korean, Dai, De'ang, Dong, Derung, Gelao, Hani, Li, Maonan, Miao, Naxi, Nu, Pumi, She, Tujia, Tibetan, Zhuang, etc. | (Xiao et al., 2022) |
| Xiang Cai | *Eryngium foetidum* L. | Polyphenols, Rutin, Essential oils, Chlorogenic acid | Whole grass have Antioxidant, Antibacterial, Antitumor, Anti-anxiety | Dai, Hani, Jino, Lisu, Li, Wa, Yao | (Xiao et al., 2022) |
| Tu Dang Gui/Ye Dang Gui | *Angelica decursiva* (Miq.) Franch. & Sav. | Organic acids, Essential oils, Polysaccharides | Roots have Antibacterial, Anti-inflammatory, Antitumor | She, Tujia, Yao | (Xi et al., 2009) |
| Du Huo | *Aralia cordata* Thunb. | Coumarins, Flavonoids, Essential oils | Roots have Antibacterial, Antioxidant, Anti-inflammatory | Qiang, Tujia, Miao | (Yang et al., 2024) |
| E Jiao Ban | *Pimpinella diversifolia* DC. | Essential oils | Roots have Anti-inflammatory, Anti-allergic, Antimicrobial | Dong, Lisu, Miao, Nu, She, Tujia, Yao | (Dong et al., 2021) |
| Jia Zi Cao/Zhan Shen Cao | *Torilis scabra* DC. | Amino acids, Vitamins, Polyphenols | Whole grass have Blood circulation enhancement, Anti-inflammatory, Analgesic | Naxi | (Gong, 2001) |
| tu dang gui | *Angelica biserrata* (R.H.Shan & Yuan) C.Q.Yuan & R.H.Shan | Organic acids, Essential oils, Polysaccharides | Roots have Anti-inflammatory, Anti-allergic, Antimicrobial | Mongolian, Miao, Tujia | (Liu et al., 2022) |
| E Can | *Anthriscus sylvestris* (L.) Hoffm. | Steroids, Lignans, Phenylpropanoids | Roots have Anti-inflammatory, Antitumor | Mongolian, Tujia, Tibetan | (Tan et al., 2017) |
| Yi Zhi Bi/Guan Yin Lian | *Balanophora involucrata var. gracilis* Hook.f. | Proteins, Dietary fiber, Vitamins | Roots and Leaves have Antibacterial, Anti-inflammatory, Antioxidant | Bai, Lisu, Naxi, Qiang, Tujia, Yi | (Wei et al., 2020) |
| Bai Hua Cai/Sui Mi Cai | *Cardamine leucantha* (Tausch) O.E.Schulz | Essential oils, Triterpenes, Flavonoids | Roots and Leaves have Anthelmintic, Arthritis treatment, Vasodilation | Korean, Miao, Qiang, She | (Lv and He, 2014) |
| Shi Song/Qing Si Long | *Lycopodium japonicum* Thunb. | Alkaloids, Terpenes | Friuts and stems have Antibacterial, Anti-inflammatory, Anti-fatigue | Buyei, Dai, Dong, Gelao, Jino, Lisu, Maonan, Miao, Nu, Qiang, She, Tujia, Yi, Tibetan, Zhuang | (Yang, 1990) |
| Shi Suan/Lao Ya Suan | *Lycoris aurea* (L'Hér.) Herb./*Lycoris radiata* (L'Hér.) Herb. | Alkaloids, Sugars | Friuts and stems have Antiviral, Antitumor, Central nervous system effects | (Laughing Buddha) Hani, Lisu, Tujia, Tibetan; (Stone Garlic) Dong, Lahu, Miao, She, Tujia, Wa, Yao, Yi | (Ji et al., 2016) |
| Huang Yao Zi | *Dioscorea bulbifera* L. | Steroids, Terpenes, Flavonoids, Phenolics | Dried Tubers have Antibacterial, Anti-inflammatory, Anti-fatigue | Achang, Dai, De'ang, Dong, Hani, Jino, Jingpo, Lahu, Lisu, Maonan, Mongolian, Miao, Mulam, Naxi, Nu, She, Tujia, Wa, Yao, Yi | (Zhu et al., 2020) |
| Zhu Sha Lian | *Dioscorea cirrhosa* Lour. | Phenolic acids, Flavonoids, Lignans | Roots have Anticancer, Alzheimer's disease, Anti-inflammatory | Buyei, Dai, De'ang, Dong, Gelao, Hani, Jingpo, Lahu, Maonan, Miao, She, Tujia, Wa, Yao, Zhuang | (Guo et al., 2021) |
| Jiu Gen Suo | *Lepisorus miyoshianus* (Makino) Fraser-Jenk. & Subh.Chandra | Polysaccharides, Flavonoids, Triterpenes | Whole grass have Anti-inflammatory, Diuretic | —— | (Fu et al., 2024) |
| Wo Long Cao | *Pyrrosia angustissima* (Giesenh. ex Diels) Tagawa & K.Iwats. | Polysaccharides, Flavonoids, Triterpenes | Whole grass have Anti-inflammatory, Antioxidant, Diuretic | —— | (Zhang et al., 2024) |
| Long Xu Cao/Xian Ren Tou Fa | *Usnea longissima* Ach. | Ketones, Terpenes | Dry ground have Antispasmodic, Antitumor, Antibacterial, Antitussive | Tibetan | (Ou et al., 2022) |
| Yan Shua Zi/Shua Zhu Cao | *Psilotum nudum* (L.) P.Beauv. | Flavonoids, Triterpenes, Phenolic acids | Anti-inflammatory, Antitumor | Miao, Tujia | (Tian et al., 1995) |
| Hong Nan Xing/Gou Zhao Nan Xing | *Arisaema fargesii* Buchet | Amino acids, Alkaloids | Roots and Stems have Anti-inflammatory, Antitumor | Tujia | (Song et al., 2024) |
| Shi Chang Pu/Shui Chang Pu | *Acorus calamus* L. | Phenolics, Ethers, Alkaloids | Roots have Antibacterial, Anti-inflammatory, Anti-fatigue | Buyei, Dong, Gelao, Korean, Dai, Hani, Jino, Li, Maonan, Mongolian, Miao, Tujia, Yi, Tibetan, Zhuang | (Cao et al., 2024) |
| Ban Jie Lan/Xue Li Jian | *Arisaema decipiens* Schott | Alcohols, Ethers, Glycosides | Roots and Stems have Antibacterial, Anti-inflammatory, Antioxidant | Lisu | (Ran et al., 2015) |
| San Bu Tiao/Ma Yu Zi | *Pinellia ternata* (Thunb.) Makino | Essential oils, Alcohols | Stems have Anti-inflammatory, Anti-allergy, Antimicrobial | Buyei, Korean, Dong, Hani, Mongolian, Miao, Naxi, She, Tujia, Wa, Yao, Tibetan, Zhuang | (Tang et al., 2021) |
| Tie Si Cao/Tie Xian Cao | *Adiantum capillus-veneris* L. | Proteins, Dietary fiber, Alcohols | Roots、Leaves and Stems have Antibacterial, Anti-inflammatory, Antioxidant | Dai, De'ang, Dong, Hani, Jingpo, Miao, Naxi, Tujia, Yi, Zhuang, Taiwanese minorities | (Yang et al., 2023) |
| Yan Feng Teng/Shang Shu Wu Gong | *Handroanthus serratifolius* (Vahl) S.O.Grose | Total saponins, Essential oils | Roots have Hemolytic, Antibacterial, Antiviral | Buyei, Dong, Lisu, Miao, Naxi, Nu, She, Tujia, Wa, Yao, Yi, Tibetan | (Qin et al., 2010) |
| Ci Lao Bao/Que Er Bu Zhan | *Aralia elata* (Miq.) Seem. | Triterpene saponins | Roots have Anti-inflammatory, Antitumor, Blood sugar lowering | Yao, Korean, Tujia, Zhuang, Yao, Maonan, Qiang, She, Dong | (Sun et al., 2022) |
| Zhu Jie Ren Shen | *Panax pseudoginseng subsp. himalaicus* H.Hara | Amino acids, Saponins | Dry roots and stems have Sedative, Analgesic, Anticonvulsant | Bai, Achang, De'ang, Derung, Jingpo, Lahu, Lisu, Miao, Qiang, Tujia, Yi, Tibetan, Zhuang | (Wang et al., 2021) |
| Kou Zi Qi | *Panax bipinnatifidus* Seem. | Triterpene saponins | Dry roots and stems have Analgesic, Antitumor | Achang, De'ang, Lisu, Miao, Naxi, Qiang, Tujia, Yi, Tibetan | (Wang et al., 2020) |
| Shan He Ye/Yi Wan Shui | *Diphylleia sinensis* H.L.Li | Lignans, Flavonoids, Phenolics | Roots have Antitumor, Antiviral | Miao, Tujia | (Huang et al., 2018) |
| Ci Huang Lian/San Ke Zhen | *Berberis julianae* C.K.Schneid. | Alkaloids | Roots and Stems have Antibacterial, Anti-inflammatory, Hypotensive, Detoxifying | (Porcupine spikes) Buyei, Dong, Gelao, Maonan, Miao, Tujia, Yi, Zhuang; (Mimic porcupine spikes) Miao | (Li et al., 2023) |
| He Ye Lian | *Dysosma versipellis* (Hance) M.Cheng ex T.S.Ying | Aristolochic toxins | Whole grass have Antitumor, Antibacterial, Antiviral | Buyei, Korean, Dong, Gelao, Hani, Miao, Mulam, Tujia, Yao, Yi, Zhuang | (Jiang et al., 2024) |
| Ji Wei Cao | *Pedicularis chinensis* Maxim. | Cycloether terpenes, Phenylpropanoid glycosides, Triterpene saponins | Whole grass have Antipyretic, Anti-inflammatory, Antioxidant | Tibetan | (Li and Tang, 2019) |
| Tian Guan Zi/Chao Tian Guan | *Osbeckia crinita* Benth. ex Naudin | Flavonoids, Organic acids, Steroids | Roots have Antibacterial, Anti-inflammatory | Miao, She, Yao | (Zhang et al., 2022) |
| She Bu Jian/Tai Jue | *Sceptridium ternatum* (Thunb.) Lyon | Total flavonoids, Polysaccharides, Saponins | Roots have Antibacterial, Antioxidant, Antitumor | Achang, Bai, Buyei, Dong, Gelao, Miao, Tujia, Tibetan, Yao | (Ma et al., 2020) |
| Yi Duo Yun | *Botrypus virginianus*  (L.) Holub | Flavonoids, Amino acids, Essential oils | Roots and Stems have Anti-inflammatory, Fever reduction | Miao, Tujia | (Li et al., 2022) |
| Tu Huang Lian/Duan Chang Cao | *Corydalis aurea* Willd. | Alkaloids, Rutin, Quercitrin, Cardiac glycosides, High vitamin C | Whole grass have Antiviral, Anti-inflammatory, Diuretic | Tujia, Zhuang | (Wang et al., 2022) |
| Shui Huang Lian/Yi Dian Xue | *Eomecon chionantha* Hance | Alkaloids | Whole grass have Anti-inflammatory, Antibacterial, Blood sugar reduction | Dong, Miao, She, Tujia, Yao, Zhuang | (Zhang et al., 2009) |
| Shui Hu Lu | *Eichhornia crassipes* (Mart.) Solms | Phenolics, Tannins, Flavonoids | Fruit、Roots and Leaves have Antioxidant | Dai, Hani, Mulam, Tujia, Yao | (Chen et al., 2018) |
| Yuan Wei | *Iris tectorum* Maxim. | Flavonoids, Triterpenes, Quinones | Roots and Stems have Antitumor, Anticancer, Antimicrobial | Buyei, Dai, Dong, Gelao, Kazakh, Lisu, Maonan, Miao, Mulam, Tujia, Yao, Yi, Tibetan, Zhuang | (Tian et al., 2024) |
| Bian Zhu Gen | *Iris japonica* Thunb. | Vitamin C | Roots and Stems have Analgesic, Insecticidal, Laxative | Dong, Tujia, Yao, Yi | (Qin et al., 2003) |
| Jian Xue Fei/San Bai Bang | *Toddalia asiatica* (L.) Lam. | Terpenes, Flavonoids, Steroids | Roots have Antitumor, Anti-inflammatory, Antioxidant | Buyei, Dai, De'ang, Dong, Hani, Jino, Jingpo, Lahu, Lisu, Maonan, Miao, Mulam, She, Tujia, Wa, Yao, Yi, Zhuang | (Li et al., 2023) |
| Xiang Ye Zi/Wu Yao | —— | Sesquiterpenes, Alkaloids, Essential oils | Leaves Anti-inflammatory, Hepatoprotective, Lipid-lowering | (Wu Yao) Dong, Mongolian, Miao, She, Tujia, Yao, Zhuang; (Xiang Ye Zi) Yao | (Cheng et al., 2024) |
| Ba Zhao Jin Long/Kai Hou Jian | *Ardisia crenata* Sims/*Ardisia crispa* (Thunb.) A.DC. | Triterpene glycosides, Flavonoids, Coumarin derivatives | Roots and Leaves have Analgesic, Anti-inflammatory, Anticancer | Achang, Dai, De'ang, Dong, Hani, Jino, Jingpo, Lahu, Lisu, Jing, Lahu, Lisu, Miao, Mulam, She, Tujia, Wa, Yao, Yi, Zhuang, Taiwanese minorities | (He and Xiong, 2014) |
| Ai Di Cha/Ai Jiao Cha/Ai Cha Feng | *Ardisia japonica* (Thunb.) Blume | Coumarins, Flavonoids | Whole grass have Expectorant, Anticoagulant, Blood circulation improvement | Dai, Dong, Jingpo, Lahu, Lisu, Maonan, Miao, She, Tujia, Yao, Zhuang | (Xie et al., 2024) |
| Zong Shu Gen | *Trachycarpus fortunei* (Hook.) H.Wendl. | Saponins | Roots have Hemostatic, Anti-inflammatory, Antibacterial, Analgesic | Achang, Bai, Buyei, Dai, Dong, Gelao, Hani, Lisu, Miao, Naxi, Nu, She, Tujia, Wa, Yao, Yi, Zhuang, Maonan | (Luo and Chen, 1992) |
| San Kuai Wa | *Oxalis griffithii* Edgew. & Hook.f. | Alkaloids, Flavonoids, Sugars | Whole grass or Roors have Anti-inflammatory, Diuretic, Antioxidant | Achang, Bai, Buyei, Dai, De'ang, Dong, Gelao, Hani, Jingpo, Lisu, Maonan, Miao, Naxi, Nu, Qiang, She, Tujia, Wa, Yao, Yi, Zhuang, Tibetan | (Deng, 1985) |

**References**

Bing F., Chang Q., Hao J., Zhang J., 2007. Effect of Chinese scallion preparation on anti-lipid peroxidation injury and cytokine injury in rats with fatty liver. Journal of Chinese Medicine. 446–449. <https://doi.org/10.13288/j.11-2166/r.2007.05.039>.

Cao N., Ye Z., Mo B., Wang N., Chen H., 2024. To explore the potential mechanism of acorus Chuanxiong in the treatment of Alzheimer’s disease based on network pharmacology and molecular docking technology. Journal of Chinese Medicine Library and Information. 48, 81–87.

Chen B., Xie L., 2015. Treating 300 cases of eczema with Jiuliguang supplementing. Our Health (Academic Edition). 9, 29.

Chen H., Yan B., Qin Z., Wu H., Yang Y., 2015. Study on the chemical constituents of the fruit of ground wasp. Chinese patent medicine. 37, 2674–2677.

Chen J., Yu H., 2023. Progress in the research of the seedling medicine Vermilion. Chinese Journal of Ethnic Medicine. 29, 53–57. <https://doi.org/10.16041/j.cnki.cn15-1175.2023.02.021>.

Cheng J., Pan X., Tao J., Li B., Ma L., Li J., 2024. Analysis of nutrient composition fat and fatty acid in leaves and stems of rosemary. Food Safety guide. 45–47. https://doi.org/10.16043/j.cnki.cfs.2024.21.041.

Chen L., Gao J., Liang H., Lei S., Wang D., 2016. Analysis of nutrient composition and mineral element content of wild onion. Modern Agricultural Technology. 283.

Chen L., Chen Z., Sun J., Liu J., Wang X., 2024. Effects of harvesting time, growing years and processing methods on the content of chloroformin in different parts of Aconitum japonica. Wild plant resources in China. 43, 44–49.

Chen L., Yan P., Xie G., Luo G., Zhang C., Wu W., 2018. Study on the extraction of total flavonoids from the roots, stems and leaves of water hyacinth and its analgesic and anti-inflammatory effects. Guangdong Chemical Industry. 45, 44–46.

Chen M., You Y., 2010. Revision and supplement of biological characteristics of Flamewort macrophyllum. Resource development and marketing. 26, 102–103.

Chen M., Kang S., Cheng H., Wan D., Yang X., Lu R., 2023. Study on chemical constituents of Cypress Ishigami. Proprietary Chinese medicine. 45, 1156–1160.

Chen Q., 2016. Study on antioxidant activity of seed oil of Roxanthus Roxanthus, Cherry blossom and red seed. Guizhou medicine. 40, 587–588.

Chen Q., 1988. Identification of white medicine seed and tiger ginger. Traditional Chinese medicine. 24–25. https://doi.org/10.13863/j.issn1001-4454.1988.06.015.

Chen R., Zhang Q., 2023. Experimental study on the intervention of lycopolysaccharide-induced cardiomyocyte apoptosis and oxidative stress damage induced by extract of scallion white. Journal of Integrated Traditional Chinese and Western Medicine Cardio-cerebral Vascular Disease. 21, 1615–1619.

Chen T., Tang F., Fang L., Tang J., 2011. Study on the extraction technology of herbicidal active substances from bamboo leaves. Journal of Anhui Agricultural University. 38, 81–86. <https://doi.org/10.13610/j.cnki.1672-352x.2011.01.022>.

Chang T., Huang R., Yang L., Luo Y., 2023. Research progress on chemical constituents and pharmacological activities of local ginseng. Research and development of natural products. 35, 693–704. <https://doi.org/10.16333/j.1001-6880.2023.4.016>.

Chen T., Wu H., Huang S., Hu C., 2022. A preliminary study on the quality standard of Mulberry leaves. Science in Guizhou. 40, 46–49.

Cui X., Wu K., Meng W., Wang Y., Li W., Feng C., Zhan Z., 2024. A textual research on the herbal medicine of the classical famous herbs. Journal of Chinese Experimental Formulae. 1–21. https://doi.org/10.13422/j.cnki.syfjx.20241268.

Chen Y., Lu C., Lv S., Wei J., Lin X., Huang Q., 2016. Effects of isoorientin Polygonatum on NF-κB signaling pathway in CCl4 induced liver fibrosis rats. Traditional Chinese medicine. 39, 2318–2323. <https://doi.org/10.13863/j.issn1001-4454.2016.10.040>.

Chi Y., Tian T., Li Xi., He F., 2008. Research progress of medicinal value and tissue culture of mountain turtle. Chinese National Folk Medicine. 17, 13–15.

Deng C., 1985. Bite from a three-piece wager snake. Sichuan traditional Chinese medicine. 53.

Dong F., Ren L., Xu K., Guo H., Li P., 2024. Research progress on chemical constituents and toxicity of Xanthium fructus. Straits Pharmacy. 36, 21–24.

Dan L., Gao T., Zhang D., Huang W., Zhang H., Song X., Wang W., Li Y., 2022. Study and analysis of chemical constituents and pharmacological effects of Lambidium japonica. Journal of Shaanxi University of Chinese Medicine. 45, 19–27. <https://doi.org/10.13424/j.cnki.jsctcm.2022.06.004>.

Dong Q., Li J., Liu Y., Yang G., Hu Y., Jiang Y., Liu Q., 2021. Study on the medicinal effect of Anise heterophyllum extract. Chinese practical medicine. 16, 197–200. https://doi.org/10.14163/j.cnki.11-5547/r.2021.30.078.

Duan X., Liang X., Ge Y., Ou S., Zhong T., 2017. cDNA cloning and expression analysis of KdFBX gene in rhizoma macrophylla. Journal of Agricultural Biotechnology. 25, 1961–1969.

Fan W., Gao A., Gong J., Ni S., Li N., Zhang X., Zheng H., Lu F., Wu Y., 2011. Global pharmaceutical research of begonia. Journal of Liaoning University of Chinese Medicine. 13, 59–61. https://doi.org/10.13194/j.jlunivtcm.2011.02.61.fanwj.078.

Feng Y., Guo Y., Li M., Wei C., 2014. Pharmaceutical research on Coiling dragon and Qisheng. Journal of Northwest University (Natural Science Edition). 44, 600–602. <https://doi.org/10.16152/j.cnki.xdxbzr.2014.04.066>.

Fan Z., Yin X., Jin Lili, Wang S., Jin Lihua, 2020. Research review of late red varson. Journal of Yanbian University (Natural Science Edition). 46, 182–188. <https://doi.org/10.16379/j.cnki.issn.1004-4353.2020.02.016>.

Fu Z., Xiao Y., Xu H., Zou J., Fen C., Shi X., Cao G., 2024. Interspecies relationship, ecological niche and community stability of main woody plants of Masson pine forest in Ruyuan Grand Canyon, Guangdong Province. Journal of ecology. 1–10.

Gao F., Luo F., Gao J., He L., 2019. Research status and development prospect of wild flower fire grass. Temperate Forestry Research. 2, 54–58.

Cao H., Yang C., Zhang Z., Li B., Zhong M., Zhang K., 2024. Total gentioside regulates the SREBP-1C/ACC1/FASN signaling pathway to alleviate alcoholic liver disease. Pharmacology and clinic of Chinese medicine. 1–14. https://doi.org/10.13412/j.cnki.zyyl.20240724.001.

Gao L., Zhang G., Ma W., Wen D., 2020. Progress in the research of nine-headed lion grass. Yunnan Chemical Industry. 47, 3–5.

Guo N., Zhao Y., Sun Y., Wang A., Xu L., Liang A., 2021. Qualitative and quantitative analysis of Aristolochic acids in Cinnabardia by UPLC-QTOF-MS/MS. Journal of Chinese Experimental Formulae. 27, 162–170. https://doi.org/10.13422/j.cnki.syfjx.20210499.

Guo Q., Wang Y., Xu S., Gao J., Cheng S., Yan Y., Zhang G., Li Y., 2024. The textual research of Rhubarbcy. Central South Pharmacy. 1–7.

Gong S., 2001. Pharmacognosy study of Pseudocomfrey Xinjiangensis from India. Foreign Medicine (Chinese Medicine Branch). 235.

Guan X., Shi L., Wang X., Chen D., 2024. Research progress on chemical constituents and pharmacological effects of Deerweed. Research on Drug evaluation. 47, 650–656.

Guan X., Shi L., Wang X., Chen D., 2024. Research progress on chemical constituents and pharmacological effects of Deerweed. Research on Drug evaluation. 47, 650–656.

Guo Y., Li M., Song P., Gao H., Fu X., 2023. Study on the ancient and modern application of Marine Chinese medicine. Journal of Shandong University of Chinese Medicine. 47, 689–699. <https://doi.org/10.16294/j.cnki.1007-659x.2023.06.001>.

He G., Xiong P., 2014. Study on alkaline extraction technology of petrobrassin from Pulchaeocarpus chinensis. Journal of Guiyang University (Natural Science Edition). 9, 64–68. https://doi.org/10.16856/j.cnki.52-1142/n.2014.02.018.

He M., Yu J., Wang Y., Zhou J., Hou K., 2024. Research progress on new extraction technology of active ingredients from bupleurum. Yunnan Chemical Industry. 51, 14-16+25.

Huang C., Ma H., Jiang J., Wang F., Xiong X., Cheng L., Huang W., 2018. Study on the chemical constituents of flavonoids in Lotus leaves. Central south pharmacy. 16, 589–592.

Huang S., Li X., Zhou J., Li J., Hu X., He Y., A T., Zhang H., Ma D., 2017. Allelopathic effects of water extract from Capsilla sinensis on growth and photosynthetic characteristics of broad bean seedlings. Southwest Agricultural journal. 30, 542–546. https://doi.org/10.16213/j.cnki.scjas.2017.3.010.

Huang X., Pan H., He W., Xiong X., 2007. Effect of water extract of Mentholium montanum on immune function of mice. Tissue Engineering Research and Clinical Rehabilitation in China. 6808–6810.

Hu Y., Dang S., 2024. Study on extraction technology and biological activity of gingerol from ginger. Chinese seasoning. 49, 196–199.

Jiang C., Yang B., Tian T., Li X., 2024. Study on the effect of Nrf2 pathway regulated by Zingerin on abnormal proliferation of colon cells NCM460 induced by high glucose. Hebei Pharmaceutical. 46, 2085–2090.

Jiang J., Xu Qi., Che L., Lai Z., Tian W., Chen H., 2024. Study on alkaloids in Lotus leaf and their antilipidemic activities. Chinese journal of traditional Chinese Medicine. 1–7. https://doi.org/10.19540/j.cnki.cjcmm.20240626.201.

Jiao W., Li F., Nan L., Yang X., Jin Z., 2024. Research progress of gentian medicinal resources. Journal of Gansu University of Chinese Medicine. 41, 80–83. <https://doi.org/10.16841/j.issn1003-8450.2024.03.15>.

Ji Y., Xin G., Qu Z., Zou Xi., Yu M., 2016. Research progress on chemical constituents and pharmacological effects of alkaloids in Lycoris L. Chinese herbal medicine. 47, 157–164.

Kong C., Liu Z., Ye D., Zhang Y., Liu Y., 2023. Study on chemical constituents and antitumor activity of geranium geranium. Modern Chinese traditional medicine. 25, 1187–1193. https://doi.org/10.13313/j.issn.1673-4890.20230307005.

Li B., Sun Y., Shi C., Zhang W., Kang Y., Wang Q., Kuang H., 2023. Research progress of Mimosa. Traditional Chinese medicinal materials. 46, 3153–3162. <https://doi.org/10.13863/j.issn1001-4454.2023.12.041>.

Lv B., He Y., 2014. Research progress of cauliflower. Modern agricultural science and technology. 74-75+81.

Liu H., Zhou X., Yan D., Gao X., Tang L., Wang F., Li B., Liu Hongdong, 2024. Research progress on chemical constituents, pharmacological actions and quality markers of Achyranthes sinensis. Jiangxi traditional Chinese medicine. 55, 70–77. <https://doi.org/10.20141/j.0411-9584.2024.08.20>.

Li J., Wang J., Li Jun, 2022. Research progress on the extraction, isolation, identification and antioxidant activity of flavonoids from Polystichum anthoides. Modern medicine and hygiene. 38, 1334–1337.

Liang G., Shi D., Shi X., 2024. Visualized literature analysis of Gastrodia Guteng drink based on CiteSpace. Journal of Chinese Medicine Library and Information. 48, 99–104.

Liu G., Yang Y., Yang J., Chen L., 2024. Progress in research and application of Notoginseng residue. Yunnan Chemical Industry. 51, 28–32.

Li L., Dai M., 2023. Literature research on star anise. Traditional Chinese medicinal materials. 46, 773–779. <https://doi.org/10.13863/j.issn1001-4454.2023.03.041>.

Tian M., Zhao J., Liu J., 2024. Effect of irisin regulating JAK2/STAT3 signaling pathway on periodontal tissue injury in rats with periodontitis. Chinese aesthetic medicine. 33, 22–26. https://doi.org/10.15909/j.cnki.cn61-1347/r.006449.

Li Q., Tang S., 2019. Growing methods of wool , ryegrass and chicken foot grass. Rural new technology. 11–13.

Li S., Huang X., Peng J., Zhu H., Liu Y., Li M., Zhong L., Ji Q., Kuang J., Ke W., 2022. Determination and evaluation of main nutrients and heavy metals in germplasm resources of watercress. Chinese Agricultural Science Bulletin. 38, 56–61.

Lu T., Hu Z., Sun X., Lu J., 2023. Qualitative study on chemical constituents of Codonopsis sinensis. Guangzhou Chemical. 51, 69-70+95.

Li X., Liu C., Liu L., Zhang D., Jiang Y., Zhang H., Song X., Li Y., Wang W., 2024. Research progress on chemical constituents and pharmacological effects of Rhizoma japonicum. Proprietary Chinese medicine. 46, 1600–1609.

Li X, Deng X., Xu’ Y., Meng H., Tang F., 2023. Comparative study on different extraction methods of effective components from different products of three needles. Chinese Journal of Ethnic Medicine. 29, 55–57. https://doi.org/10.16041/j.cnki.cn15-1175.2023.08.028.

Lia Y., Guo J., Xu L., Yang S., 1999. Chemical composition of black wind vine. Acta Pharmacologica Sinica. 48–50. https://doi.org/10.16438/j.0513-4870.1999.03.012.

Li Y., Mao Y., Liang Z., Yang C., Wu J., Yuan Z., Yuan L., 2023. Research progress on pharmacological action of Fructus purpurea. Chinese animal husbandry and Veterinary medicine. 50, 2998–3006. <https://doi.org/10.16431/j.cnki.1671-7236.2023.07.039>.

Li Y., Wei L., Zhang D., Chen W., 2024. Research status, application and prospect of wormwood. Seed Industry in China. 21–27. https://doi.org/10.19462/j.cnki.zgzy.20240430003.

Liu X., Han H., Qin B., Wang J., 2019. Study on chemical constituents of lungform grass. Journal of Central South University for Nationalities (Natural Science Edition). 38, 215–218.

Li X, Li Xiaoyan, Xu Y., Sun X., Liu J., Yan R., Tian M., 2017. Comparison of polysaccharide content in different parts of seedling and medicinal wild cotton. Chinese Journal of Ethnic Medicine. 23, 36–37. https://doi.org/10.16041/j.cnki.cn15-1175.2017.10.029.

Liu Y., 2020. Tinospora sagittata (Oliv.) Gagnep. Chinese National Folk Medicine. 29, 127.

Liu Y., Zhang Q., Peng Y., Wu Z., Lin G., Xu Y., Luo Y., 2015. Pharmacognosy study of four primitive tiles of four kinds. Chinese journal of traditional Chinese Medicine. 40, 4177–4181.

Liu Y., Huang S., Chen H., Xu K., Xu Y., Shao A., 2023. Effect of Huoxuedan extract on contraction of heart, duodenum and gastrocnemius muscle of bullfrog. Chinese Journal of Veterinary Medicine. 59, 141–145.

Liu Y., Miao J., Tian S., Zhang M., Xu E., 2021. Research progress on chemical constituents and pharmacological effects of Verbena. Henan traditional Chinese medicine. 41, 294–299. <https://doi.org/10.16367/j.issn.1003-5028.2021.02.0067>.

Luo X., Wang W., Hu M., Zhang X., Shen X., 2024. Research progress on antiviral chemical constituents and effects of Flos lonicerae. Journal of Shaanxi University of Chinese Medicine. 47, 138–143. <https://doi.org/10.13424/j.cnki.jsctcm.2024.02.027>.

Luo Y., Chen D., 1992. Study on chemical constituents of palm root. Chinese Herbal Medicine. 23, 400.

Lv J, Ma Y., Xin S., Yang H., Mo H., Yang T., Liu Q., 2024. Origin processing, quality standard and product development strategy of Neijiang Aspartame. Journal of Traditional Chinese Veterinary Medicine. 43, 32–38. <https://doi.org/10.13823/j.cnki.jtcvm.2024.057>.

Lv Jing, Tian X., Xiang Z., Chen C., 2024. Research progress on chemical constituents and pharmacological activities of Herba chinensis. Journal of Liaoning University (Natural Science Edition). 51, 121–129. https://doi.org/10.16197/j.cnki.lnunse.2024.02.004.

Ma B., Tao Z., Zhou R., Wang X., Lv Q., Sun S., Wang H., Gao J., Zhang C., Chen F., 2023. Optimization of extraction conditions and study on activity of functional components of Dieffensia floricolor. Zhejiang Agricultural Journal. 35, 383–393.

Ma J., Lin Z., Liu Y., Liu X., Chen X., 2020. Extraction and antioxidant activity of polyphenols from Mallow alba. Journal of tropical crops. 41, 1450–1458.

Mu D., Su R., Na S., Bu R., Bai W., Qu B., 2019. Study on quality standard of Mongolian golden kidney. Global Chinese Medicine. 12, 1012–1017.

Mu H., Ye W., Chen Y., Zhu J., Cao H., 2008. Effects of different levels of phosphorus nutrition on photosynthesis and growth of cinnabar root and Sanshuei Dan. Botanical research in Wuhan. 514–519.

Meng L., 1994. Discrimination of Ampelopsis japonica rattan. Chinese forest side product. 15–16.

Miao T., Tao M., 周 X., Liu Y., Zhang Y., Meng D., 2024. Separation and identification of chemical constituents of Nepenthes officinalis. Journal of Shenyang Pharmaceutical University. 41, 417-423+492. <https://doi.org/10.14066/j.cnki.cn21-1349/r.2023.0645>.

Ni J., Zhang X., Lin N., Wang H., Luo X., 2020. Progress in clinical application and quality control of Haijinsha. Journal of Chinese Medicine. 38, 17–20. <https://doi.org/10.13193/j.issn.1673-7717.2020.09.005>.

Ou Q., Li Z., He L., Hu M., Lu W., 2022. Study on the bacteriostatic effect and stability of total flavones in the roots of Baleen grass. Modern food. 28, 192–196. https://doi.org/10.16736/j.cnki.cn41-1434/ts.2022.01.051.

Peng R., Pu T., Yang W., Xu B., Li K., Huang G., 2023. Study on extraction technology and bacteriostatic effect of total alkaloids from Plantain. Shandong chemical industry. 52, 82-84+93. <https://doi.org/10.19319/j.cnki.issn.1008-021x.2023.23.018>.

Pan S., Wang Q., Liu Z., Zhang Q., Wei S., Shuai G., Zhang M., Huang Y., Hu X., n.d. Effect of lactic acid bacteria fermentation on hypoglycemic and hypolipidemic activity of loquat juice in vitro. Food industry science and technology. 1–13. https://doi.org/10.13386/j.issn1002-0306.2024050345.

Pan Y., Wang T., Ma G., Guo L., 1998. Determination of petroglycin in Gandujing granules by RP-HPLC. New Chinese medicine and clinical pharmacology. 44-46+64. <https://doi.org/10.19378/j.issn.1003-9783.1998.03.016>.

Qiu B., Xu L., Wei X., Kang M., Lin L., 2014. Chemical constituents of anthraquinones in Herba chinensis. Journal of Tropical and Subtropical Botany. 22, 507–510.

Qi L., Niu X., 2011. Characteristics and microscopic identification of Sedum rotunda. Traditional Chinese medicinal materials. 34, 1867–1868. <https://doi.org/10.13863/j.issn1001-4454.2011.12.026>.

Qiao L., Zhang X., Ding Y., Zhang Y., 2006. Preparation and clinical curative effect of snake ginseng antipruritic lotion. When Zhen Chinese medicine. 1733–1734.

Qin X., Guo H., Ren X., 2010. Identification of Morinda officinalis, Caryophyllum, Sichuan tiger thorn. Anhui medicine. 14, 159–160.

Qiu Y., Li M., Ji Y., 2024. Active ingredients, pharmacological action and application prospect of Zizhidium in livestock and poultry production. Chinese Journal of Veterinary Medicine. 58, 89–94.

Qin J., Chen T., Lv Q., Tian Y., 2003. Determination of volatile oil components in bamboo root. Journal of Guizhou University of Technology (Natural Science Edition). 31-32+45.

Ran H., Zhao H., Du J., Wu S., 2015. Preliminary study on liver and kidney toxicity of mice with half-broken seedling medicine. Laboratory animal science. 32, 27-30+4.

Sun J., Yan M., Zhai J., Ding L., Sun Y., Pei L., 2022. To explore the mechanism of treating sarcopenia by pricking aging buds based on network pharmacology. Journal of China Medical University. 51, 980–986.

Song M., Zhang X., Li J., Li H., Xu D., 2024. Study on chemical constituents of Arisaema heterophyllum. Ginseng research. 36, 11–15. https://doi.org/10.19403/j.cnki.1671-1521.2024.01.003.

Shu Y., Guo J., Xu W., Zhang Y., Sun K., Shi X., Zeng L., 2023. Objective: To investigate the mechanism of the treatment of Alzheimer’s disease by using network pharmacology and molecular docking. Chinese Journal of Gerontology. 43, 5484–5492.

Shen Y., 2014. Development status and countermeasures of Ginkgo biloba in Caomiao Town, Dafeng, Jiangsu Province. Chinese Horticultural Abstract. 30, 67–68.

Tu D., Liang M., Lu D., Chenxian X., Liang Q., Li X., Wang L., 2021. Rubiaceae Yao drug species and standard arrangement. Information Journal of Chinese Medicine. 28, 6–12. <https://doi.org/10.19879/j.cnki.1005-5304.202004202>.

Tan H., Ma C., Geng Y., 2017. Research progress on the chemical constituents and pharmacology of A. coli in recent 5 years. Journal of Chinese Medicine. 35, 1194–1196. https://doi.org/10.13193/j.issn.1673-7717.2017.05.038.

Tong J., Liu H., Liu Y., Zhang F., Sun L., Chen Y., 2023. Basic study on hypoglycemic active substances of Golden osmanthus flower based on HPLC-ESI-QTOF-MS technique. Acta Pharmacologica Sinica. 58, 750–759. <https://doi.org/10.16438/j.0513-4870.2022-1071>.

Tian J., Jin G., Ma Y., Sun S., 1995. Study on extraction of tobacco extract from tobacco (tobacco dust) with CO2 solvent. Chinese Journal of Tobacco. 75–79.

Tang T., Zhu J., Zhou M., Huang F., Huang H., Cheng H., 2022. Analysis of volatile oil from Kudzu stem and extraction technology of total flavonoids from Kudzu root. Chinese Seasoning. 47, 182–187.

Tang J., Liu Y., Zhang H., Jiang Y., Zhang D., Wang W., Song X., Li Y., 2024. Research progress on chemical constituents, pharmacological and toxicological effects of Radix Notoginseng. Journal of Shaanxi University of Chinese Medicine. 1–8.

Tan X., Wang L., Huang Y., Yuan J., Liu B., 2023. Experimental study on chemical composition and cell proliferation of round fruit plate. Traditional Chinese medicinal materials. 46, 384–389. <https://doi.org/10.13863/j.issn1001-4454.2023.02.020>.

Tan Y., Fu Y., Huang R., Liu L., Li X., Luo Y., Gao M., 2024. Analytical study on the quality and the change of characteristic flavor substances in the fermentation process of Hubei Wufeng Gastrodia. Food and fermentation industry. 1–10. <https://doi.org/10.13995/j.cnki.11-1802/ts.040168>.

Tang Y., Wang H., Pan Z., Zhang J., Li K., 2021. Pinellia new variety Hema taro 1. Chinese seed industry. 114–115. https://doi.org/10.19462/j.cnki.1671-895x.2021.10.041.

Wang F., Dan L., Zhou W., Li Y., Huang W., Zhang H., Song X., Zhang D., Wang W., 2022. Research progress on chemical constituents, pharmacological action and clinical application of cinnabar. Journal of Shaanxi University of Chinese Medicine. 45, 10–15. <https://doi.org/10.13424/j.cnki.jsctcm.2022.05.003>.

Wu G., Lin Y., Liu H., Yang X., Liu A., Wei K., Liao J., 2023. Non-targeted metabolomics reveals the differential metabolites and their main metabolic pathways in different parts of the sunflower. Modern Chinese traditional medicine. 25, 1582–1591. https://doi.org/10.13313/j.issn.1673-4890.20221208001.

Wang H., Yang S., Bai S., 2018. Research progress on chemical constituents and biological activities of Chinese horseshoe herb. Food and Medicine. 20, 157–160.

Wang J., Zheng J., Ma Y., Li Y., Zhu Y., Gui Y., 2024. Research progress on nutritional characteristics of oat and quality improvement of oat noodles. Grain Processing. 49, 83–86. <https://doi.org/10.20170/j.cnki.lsjg.1007-6395.20240418>.

Wang K., Zhan Z., Liao T., Xu J., Wan J., Wei Y., Wen C., Ou Y., 2021. Research progress on chemical constituents and pharmacological activities of Phyllodendron japonicum. Wild plant resources in China. 40, 48–59.

Wan P., Xu Q., Kang W., 2010. Study on the chemical constituents of the nine-seed catenaria. Proprietary Chinese medicine. 32, 2017–2019.

Wu Q., Fan M., 2024. Research progress on Lily. Chinese food industry. 132–134.

Wu Q., Zhang F., Zhang J., Li D., Ji T., 2024. Analysis on the use of bamboo leaves in Clinical Guide Medical Records. Global Chinese Medicine. 1–6.

Wang T., Li X., Ma Q., Zhang L., 2022. Identification and analysis of a toxic plant called guillotine. Gansu medicine. 41, 70–72. https://doi.org/10.15975/j.cnki.gsyy.2022.01.028.

Wang W., Chen H., SHo G., LIao H., Zhao J., Li Q., 2022. The nectary structure and its biological significance of inbred and outbred Codonopsis. Plant research. 42, 364–372.

Wang X., 2020. Study on chemical constituents of Lobelia lobelia. Proprietary Chinese medicine. 42, 3208–3210.

Wang X., Chen J., 2024. Research progress on components and pharmacological effects of the Miao medicine of Guizhou. Traditional Asian and Pacific Medicine. 1–4.

Wang X., Yu L., Na H., Li D., Zhou X., 2016. Determination of hypericin in different medicinal parts of Mongolian herb chervil by HPLC. Chinese Journal of Ethnic Medicine. 22, 53–54. <https://doi.org/10.16041/j.cnki.cn15-1175.2016.09.033>.

Wang X., Xie Q., Liu Y., Lu S., Daniyal M., Li B., Duan S., Gong L., Liang N., Mao Y., Yu Y., Wang W., 2020. Study on triterpenoid saponins of Tujia drug Clasp Seven and their antitumor activity. Chinese herbal medicine. 51, 1831–1838.

Wang Y., Wang X., Chen Q., Zhang L., Liu Y., Tang H., 2014. Study on in situ hybridization and systematic classification of elliptic rubus, rubus rubus and seedling bubble. Journal of horticulture. 41, 841–850. <https://doi.org/10.16420/j.issn.0513-353x.2014.05.009>.

Wei Y., Yu H., Liu X., Gao qi, Yin X., Yin H., Wu J., 2020. Research progress on main chemical constituents and pharmacological effects of turmeric. Journal of Xinxiang Medical College. 37, 990–995.

Wang Z., Han L., Hao J., Hu C., Wang T., Zhang P., 2023. Research progress on chemical constituents and pharmacological activities of Elderberry. Chinese patent medicine. 45, 1936–1943.

Xiong C., Wu G., Li Y., Zeng D., Wang Y., Cheng J., Xie H., Zhang H., 2004. Differentiation of Schisandra Schisandridae varieties of the seedling medicine “blood rattan.” Chinese Journal of Ethnic Medicine. 140–145.

Xie H., Wang L., 2024. Advances in the study of chemical constituents in the plants of Cymbidium. Journal of Yunnan Minzu University (Natural Science). 1–11.

Xiong J., Ma R., Yu H., Mu L., Mo X., 2024. Research progress on the role of Leonurine in inflammation-related diseases. Fudan Journal (Medical Edition). 51, 614–619.

Xiao J., Xiang A., Zhang N., 2022. Research progress on chemical constituents and pharmacological effects of Houttuynia houttuynia. Journal of Modern Chinese and Western Integrative Medicine. 31, 1563–1567.

Xie J., Mo X., Chen X., Li Y., Huang Q., Liang P., Luo W., Pan X., 2023. Study on chemical constituents of Dendropterygium brevis. Chinese medicine Guide. 29, 38–42. <https://doi.org/10.13862/j.cn43-1446/r.2023.09.008>.

Xu S., 2006. Study on the application of ten kinds of ethnic medicine in Guizhou. World science and technology. 73–78.

Xi W., Shi D., Liu P., Jiang C., 2009. Identification of crude drug of native Codonopsis from Guizhou and its mixed products. Chinese national folk medicine. 18, 9–11.

Xu X., Li A., Kang Z., Zheng K., 2015. Research progress on chemical constituents and pharmacological activities of Vetch. Chinese Agricultural Science Bulletin. 31, 74–80.]

Xie Y, Dong Z., Bai Y., Li Y., Yan G., 2024. Study on pharmacognosy and pharmacotoxicological effects of Oleander. Asia Pacific Traditional Medicine. 1–7.

Xie Y, Yu C., Fu J., Ye K., Li B., Wei X., Hou X., 2024. Study on chemical constituents of dwarf tea. Journal of Guangxi University of Chinese Medicine. 27, 47–50.

Yao C., Hong M., Lin X., Fang Y., Zhao S., Qu J., Yang X., Zheng X., Tao F., 2023. Screening and identification of antitumor active sites of snake retrogression based on LC-MS. Chinese Traditional Medicine Science and Technology. 30, 232–235.

Yang C., Zhang W., Zhao K., Han K., Li J., Zhao Q., Jiang Z., 2024. Research progress on the chemical constituents and pharmacological effects of Leucochromis alba. Medical guide. 1–16.

Yang C., 1990. Overview of pharmacologic effects of lycopodium plants and their components. Chinese herbal medicine. 21, 41–43.

Yang J., Sun W., Suo X., Zhang H., Wei T., Xie M., 2024. Herbal research on the sex effect of winter melon seed. Journal of Liaoning University of Chinese Medicine. 26, 115–119. <https://doi.org/10.13194/j.issn.1673-842x.2024.05.021>.

Yang L., Luo W., Cui C., Gao H., He D., Do C., 2023. Study on deproteinization process and biological activity of polysaccharide from Maidenia graminis by response surface optimization. Northern horticulture. 77–85.

Yang Q., Gu Z., Sun H., 1995. Karyotype and phylogenetic significance of Tiebreaking gong with angular leaf. Plant Taxonomies. 225-229+316.

Yuan X., Li H., Yin Z., Wang H., Zhao G., 2018. Chemical constituents and in vitro anti-HBV activity of Chrysosoma chinensis. Chinese Patent Medicine. 40, 363–368.

Yang Y., Jin H., Yang B., Wang X., Ma W., Jin P., Yu L., 2024. The textual research of Gentiana Dianensis. Chinese ethnic folk medicine. 33, 55-61+83.

Yang Z., Li Q., Li Z., Chen G., Zhang C., 2024. Extraction, purification and structure analysis of polysaccharide from Roxburghius yunnanensis. Chinese seasoning. 49, 192-199+207.

Yin Z., Xu H., Yang L., Wang J., Che C., Li Y., Wang W., Song X., Zhang D., 2024. Research progress on chemical constituents, pharmacological action and clinical application of Rattan japonica. Journal of Shaanxi University of Chinese Medicine. 1–10.

Zeng B., Liu L., Shi Z., 2021. Research progress on chemical constituents and pharmacological activities of an arrow. Drug Evaluation. 18, 1275–1280. <https://doi.org/10.19939/j.cnki.1672-2809.2021.20.17>.

Zhu D., Xu L., Xu X., 2020. Research progress on chemical constituents of Fructus xanthoides. Journalof Jiangxi University of Chinese Medicine. 32, 117–121.

Zhou K., Xie J., Li Y., Wang Y., Zhang X., Liu C., 2021. Research progress on chemical constituents and pharmacological effects of the spider plant and prediction of its quality markers. Chinese Pharmacy. 32, 1391–1396.

Zhang L., Chen Y., Lu H., Shi W., Guo L., Jiang X., 2022. The regulatory effect of total phenol on lipopolysaccharide-induced inflammation in mouse macrophages. Journal of Guangxi Medical University. 39, 370–374. https://doi.org/10.16190/j.cnki.45-1211/r.2022.03.005.

Zhang M., Wang C., Liu Y., Zhao Y., Zhu J., Li Y., 2024. Inhibitory effect of salidroside on canine parvovirus replication in vitro. Journal of Animal Husbandry and Veterinary science. 1–14.

Zhi G., Huang L., Lan X., 2023. Research progress on pharmacological activity of sesame. Guangdong Chemical Industry. 50, 72-73+82.

Zhang X., Jiao X., Cheng D., Zhang L., Ding H., 2023. Research progress of chemical constituents and pharmacological effects of Tetraphora japonica and predictive analysis of quality markers. Wild plant resources in China. 42, 72–80.

Zhang X., Shu X., Gao Y., Wang Y., Lv T., Zhang Y., Huang X., Song C., Yang K., Luo G., 2020. Study on the immunomodulatory effects of the polysaccharide of Marsupia sinensis and the flavone complex of Breviscapus breviscapus. Journal of Chinese Veterinary Medicine. 39, 17–22. https://doi.org/10.13823/j.cnki.jtcvm.2020.06.004.

Zhan Y., Zhao Y., Yan B., Li S., Ren J., Ren X., Qin S., Yang M., Wang X., 2024. Study on preparation technology of codonopsis formula granules. Ginseng research. 36, 39–42. https://doi.org/10.19403/j.cnki.1671-1521.2024.04.007.

Zhang Y, Jin Z., Feng S., Hu G., Xu J., Zhao Y., 2024. Research progress on chemical constituents and pharmacological effects of Momordica sinensis. Chinese herbal medicine. 55, 1034–1045.

Zhang Y., Li J., Fan X., 2023. Comparative study on the identification of crude drugs between Cuiyuncao and Xiaocuiyuncao. Journal of Hubei University of Chinese Medicine. 25, 40–44.

Zhang Y., Zhuang Y., Lu Y., Chen Yanning, Chen Yichong, Xu S., Zhan Z., Yang C., 2024. The textual research of the classic name of corrugator. Journal of Chinese Experimental Formulae. 1–14. <https://doi.org/10.13422/j.cnki.syfjx.20240462>.

Zhang Y., Chen G., Tian H., Teng J., Tian L., 2009. Pharmacognosy of Tujia medicine Coptis. Chinese Journal of Ethnic Medicine. 15, 47–49. https://doi.org/10.16041/j.cnki.cn15-1175.2009.04.024.

Zhou Z., Hu Q., Xiao M., 2024. Optimization of extraction process, evaluation of hypoglycemic activity and composition analysis of total flavonoids from Radix japonicum. Chinese Pharmacy. 35, 1972–1978.

Zheng Z., Wang G., 2023. Research progress on antitumor pharmacological mechanism of stephanine. Wild plant resources in China. 42, 68–72.

Zhang Z., Wei Y., Yang Y., Zhang X., 2023. Improvement mechanism of Shanci mushroom polysaccharide on symptoms of hepatocellular carcinoma mice with ascites. Northwest Journal of Pharmacy. 38, 74–80.

Zhang Z., Yang F., Liu H., Xu G., Zhu Q., Yu L., Liao S., 2024. Chemical composition and α-glucosidase inhibitory activity of melon seeds. Modern food science and technology. 1–9. https://doi.org/10.13982/j.mfst.1673-9078.2024.11.1252.
